# Electrostatic changes enabled the diversification of an exocyst subunit via protein complex escape

**DOI:** 10.1038/s41477-025-02135-1

**Published:** 2025-10-31

**Authors:** Juan Carlos De la Concepcion, Héloïse Duverge, Yoonwoo Kim, Jose Julian, Haonan D. Xu, Matthew N. Watt, Sara Ait Ikene, Anita Bianchi, Nenad Grujic, Ranjith K. Papareddy, Irina Grishkovskaya, David Haselbach, David H. Murray, Marion Clavel, Nicholas A. T. Irwin, Yasin Dagdas

**Affiliations:** 1 Gregor Mendel Institute, Austrian Academy of Sciences, Vienna BioCenter, Vienna, Austria; 2 Department of Applied Genetics and Cell Biology, Institute of Molecular Plant Biology, Boku University, Vienna, Austria; 3 Division of Molecular Cell and Developmental Biology, School of Life Sciences, University of Dundee, Dow Street, Dundee, UK; 4 Heidelberg University, Centre for Organismal Studies, Heidelberg, Germany; 5 Research Institute of Molecular Pathology, Vienna BioCenter, Vienna, Austria; 6 Max-Planck-Institut für Molekulare Pflanzenphysiologie, Potsdam-Golm, Germany

**Keywords:** Plant cell biology, Phylogenetics

## Abstract

Protein neofunctionalization is a key driver of cellular complexity. However, subunits of multimeric protein complexes are often thought to be evolutionarily constrained, limiting their capacity for functional divergence. This presents a paradox in plants, where the Exo70 subunit of the exocyst—an octameric complex essential for exocytosis—has undergone striking expansion and diversification. Here we show that electrostatic changes in the N-terminal helix of Exo70 facilitated its physical and functional dissociation from the exocyst, relieving constraints imposed by complex integration. Using *Marchantia polymorpha* and *Arabidopsis thaliana*, we demonstrate that this ‘complex escape’ enables Exo70 paralogues to acquire distinct localizations, interactomes and functions independent of canonical exocytosis. Ancestral reconstructions across land plants reveal that this electrostatic shift predates the extensive radiation of the plant Exo70 protein family, with some lineages later reassociating with the complex. Our findings reveal a reversible mechanism that enabled Exo70 to circumvent the evolutionary and biophysical constraints imposed by complex integration and diversify—a mechanism that could represent a generalizable route to protein neofunctionalization and cellular innovation.

## Abstract (web summary)

The evolutionary diversification of an exocyst subunit was enabled by electrostatic shifts leading to its dissociation from the ancestral complex.

## Main

The emergence of protein complexes is a key process in cellular evolution, often driven by the duplication and specialization of existing proteins^1–4^. Multimeric protein assemblies typically evolve through the accumulation of stabilizing mutations which establish protein–protein interaction interfaces, enabling the emergence of higher-order structures^5–9^. This evolutionary trajectory can be influenced by both adaptive and non-adaptive processes, such as constructive neutral evolution^6,7,10–14^. Yet, although the emergence of multimeric complexes increases cellular diversity, it simultaneously imposes evolutionary constraints on the subunits they comprise, limiting their capacity to diversify independently.

Integration into a protein complex can limit evolutionary plasticity. Dependency on interacting partners can lead to subfunctionalization, reducing autonomy and the potential for diversification. In addition, hydrophobic entrenchment, the neutral accumulation of hydrophobic residues at interaction interfaces, hinders subunit dissociation, reinforcing these constraints^6,7,9,10^. Limitations to functional independence are exacerbated by the risk of paralogue interference where subunit duplication can disrupt protein complex stoichiometry and assembly^15–18^. This conflict can be mitigated through the spatiotemporal segregation of paralogues, achieved via differential gene expression or altered subcellular localization^17,19^. However, the lack of functional independence, the ubiquitous expression of many conserved protein complexes, and restrictions imposed by hydrophobic entrenchment often limit opportunities for paralogue isolation^20–25^. Although subunit duplication is observed in homo-multimeric complexes^4,12,26,27^, it is unclear whether the subunits of heteromeric complexes can overcome these constraints and diversify.

The exocyst offers a unique system for studying the evolution of heteromeric protein assemblies. It is a highly conserved hetero-oligomeric complex that plays a critical role in exocytosis across eukaryotes by mediating the tethering and membrane fusion of secretory vesicles^28–34^. Composed of eight subunits—SEC3, SEC5, SEC6, SEC8, SEC10, SEC15, Exo84 and Exo70^35^—exocyst proteins are organized through the coiling of their N-terminal α-helices into a helical bundle, known as CorEx^33,36–38^. While most eukaryotes encode single copies of these subunits, an exception is found in plants, where Exo70, the subunit responsible for targeting the exocyst to the plasma membrane, has undergone an extensive radiation. Indeed, plant Exo70s have diversified into dozens of paralogues, grouped into three families: Exo70I, Exo70II and Exo70III^39–42^ (Supplementary Fig. 1a).

The marked expansion of Exo70 proteins in plants raises the question of how a subunit from a conserved multimeric complex escaped its evolutionary constraints and diversified. Despite variation in expression profiles, multiple Exo70 paralogues are co-expressed within individual cells^43,44^ where they contribute to both canonical and non-canonical exocytic functions^39,45^. Paralogues of the Exo70I family, which is relatively conserved and consists primarily of Exo70 clade A, are predominantly associated with canonical exocytosis^46–49^. By contrast, paralogues from the highly diversified Exo70II family, encompassing clades B, C, D, E, F and H, participate in a broad array of distinct cellular processes. For instance, Exo70s from clades B and D are implicated in autophagy^50–52^, with *Arabidopsis thaliana* paralogues AtExo70D1, D2 and D3 functioning as selective autophagy receptors^52^—a role that may not require interaction with the exocyst complex. In monocots, clade F has been extensively expanded and plays a crucial role in pathogen recognition^53,54^, even becoming integrated into plant immune receptors through gene fusion^41,54–56^. Some degree of functional specialization also appears to result from variation in membrane targeting^39,45^. Despite this, the mechanisms liberating Exo70 from the evolutionary constraints imposed by complex integration, and the extent to which the exocyst contributes to its functional diversity, remain unknown.

To better understand subunit evolution and the diversification of Exo70, we examined Exo70 paralogues in the liverwort *Marchantia polymorpha* (hereafter Marchantia)^57^. Marchantia encodes a single copy of each of the other core exocyst subunits and only three Exo70 paralogues, each representing one of the major plant Exo70 families: I, II and III (Supplementary Fig. 1b). This reduced genetic redundancy makes Marchantia an ideal model for dissecting Exo70 evolution, especially compared with species like *A. thaliana*, which possesses 23 Exo70 paralogues^40^.

## Only a subset of Marchantia Exo70 paralogues maintain exocyst association

**Fig. 1:**
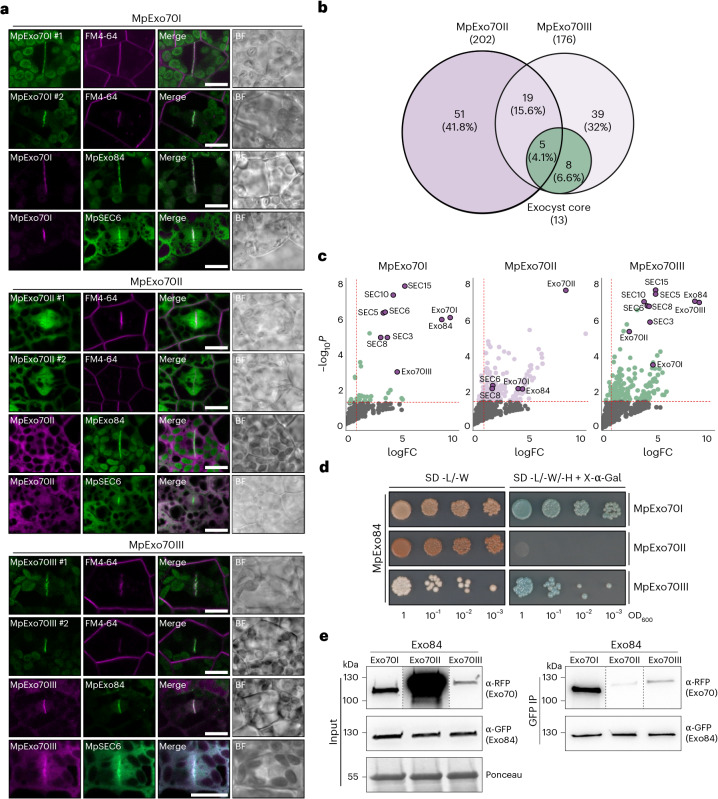
Marchantia Exo70 proteins differentially interact with the exocyst complex. **a**, Confocal micrographs of Marchantia cells from two independent lines stably expressing MpExo70:Clover (green) and stained with FM4-64 (magenta) or lines stably co-expressing MpExo70:mScarlet (magenta) with MpSEC6:Clover or MpExo84:Clover (green). The cell plate is indicated by the accumulation of FM4-64 stain or the core exocyst components MpSEC6 or MpExo84. Scale bar, 10 µm. BF, bright field. For each line, a gallery of images was collected to document variability and is presented in Supplementary Figs. 2 and 5 for FM4-64 treatment and colocalization, respectively. Each experiment was repeated at least three times with similar results. **b**, Venn diagram of three overlapping pairwise comparisons for AP-MS conducted in Marchantia: MpExo70I:Clover versus GFP control (green circle), MpExo70II:Clover versus GFP control (purple circle) and MpExo70III:Clover versus GFP control (light-purple circle). Total number of interactors for each pairwise comparison is indicated in brackets under the protein name. Number and percentage of shared interactors between pairwise comparisons are indicated in each overlapping area. Results represented are the mean from three independent replicates. **c**, Enrichment of exocyst protein copurified with MpExo70I, MpExo70II or MpExo70III compared with a control expressing free GFP and represented by a volcano plot. The horizontal dashed line indicates the threshold above which proteins are enriched (*P* value <0.05, quasi-likelihood negative binomial generalized log-linear model), and the vertical dashed line indicates the threshold where the proteins’ log_2_FC is greater than 1. For each plot, members of Marchantia exocyst complex are depicted by a purple dot with the corresponding name. **d**, Y2H assay of MpExo70 interaction with MpExo84. For each combination, 5 µl of yeast at the indicated OD_600_ was spotted and incubated on double dropout plates (lacking leucine (L) and tryptophan (W)) for yeast growth control (left), and on triple dropout media (lacking L, W and histidine (H)) supplemented with X-α-gal (right). Growth and development of blue colouration on the right indicates protein–protein interactions. MpExo70s were fused to the GAL4 DNA-binding domain, while MpExo84 was fused to the GAL4 activator domain. Accumulation of each protein in yeast cells was tested by western blot and is presented in Supplementary Fig. 11. Each experiment was repeated at least three times with similar results. **e**, Co-IP of MpExo70 proteins with MpExo84. C-terminally mScarlet-tagged MpExo70 proteins were stably co-expressed with C-terminally Clover-tagged MpExo84 in Marchantia. Immunoprecipitates (IPs) were obtained with anti-GFP magnetic beads, and total protein extracts were probed with anti-RFP or anti-GFP antibodies for MpExo70 and MpExo84 proteins, respectively. Dashed lines represent a crop and assembled image from the same blot. Each experiment was repeated at least three times with similar results. Source data

**Extended Data Fig. 1:**
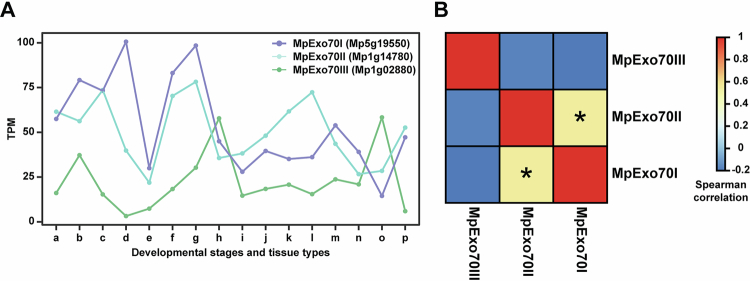
Expression levels of Exo70 paralogs in Marchantia polymorpha across developmental stages and tissue types. **(A)** Data was obtained from MarpolBase (Accessed July 2024). TPM in the Y-axis represents transcripts per million. Different developmental stages and tissue types in the X-axis are depicted with letters. a: Tak-1 thallus 9-day male Higo et al., b: Tak-1 thallus 14-day male Karaaslan et al., c: Tak-1 antheridiophore mature male Higo et al., d: Tak-1 antheridium male Higo et al., e: Tak-1 sperm cell Julca et al., f: Tak-2 archegoniophore mature female Higo et al., g: Tak-2 archegonia female Hisanaga et al., h: Cam1 x Cam2 Spores 0 h Bowman et al., i: Cam1 x Cam2 Spores 24 h Bowman et al., j: Cam1 x Cam2 Spores 48 h Bowman et al., k: Cam1 x Cam2 Spores 72 h Bowman et al., l: Cam1 x Cam2 Spores 96 h Bowman et al., m: Tak-1 gemma cup 21 day male Ishizaki et al., n: Tak-1 midrib 21 day male Ishizaki et al., o: BC3 x Tak-1 gametophytic apical cell 9-day Frank et al., p: BC3 x Tak-1 young sporophyte 13-day Frank et al. **(B)** Spearman correlation between expression data for MpExo70I, MpExo70II and MpExo70III. Correlation is 0.532 for MpExo70I vs MpExo70II, -0.203 for MpExo70I vs MpExo70III and -0.175 for MpExo70II vs MpExo70III. Asterisk depicts a significant p value of 0.03382 for MpExo70I vs MpExo70II. The statistical test is a two-sided t-test (df = 14).

To investigate the evolutionary trajectory of Exo70, we first investigated its functional diversification in Marchantia. To assess the spatiotemporal distribution of the three Marchantia Exo70 paralogues (MpExo70I, II and III), we analysed published gene expression data derived from different developmental stages and tissues^58^. This revealed that MpExo70I and MpExo70II are highly expressed and positively correlated, whereas MpExo70III is weakly expressed and transcriptionally isolated (Extended Data Fig. 1). This suggests that differential expression may segregate MpExo70III, while MpExo70I and MpExo70II co-occur. To determine whether co-expressed paralogues also colocalize within cells, we compared their subcellular localizations using fluorescent protein fusions. Constitutively expressed MpExo70I and MpExo70III both localized to the cell plate in postmitotic cells, a localization consistent with exocyst activity, as the exocyst is known to accumulate at the cell plate in *A. thaliana*^46^ (Fig. 1a and Supplementary Fig. 2). In agreement, Marchantia core exocyst subunits, MpSec6 and MpExo84, also accumulated at the cell plate, colocalizing with MpExo70I and MpExo70III, and highlighting the cell plate as a proxy for canonical exocyst function (Fig. 1a and Supplementary Figs. 3 and 4). By contrast, MpExo70II exhibited a diffuse localization, distinct from the cell plate, MpSec6, MpExo84 and the other Exo70 paralogues (Fig. 1a and Supplementary Figs. 2, 5 and 6). These results suggest that MpExo70I and MpExo70III are associated with exocytosis but separated by differential gene expression. Conversely, MpExo70I and MpExo70II may be co-expressed, but are spatially segregated, with MpExo70II apparently lacking a spatial association with the exocyst.

**Extended Data Fig. 2:**
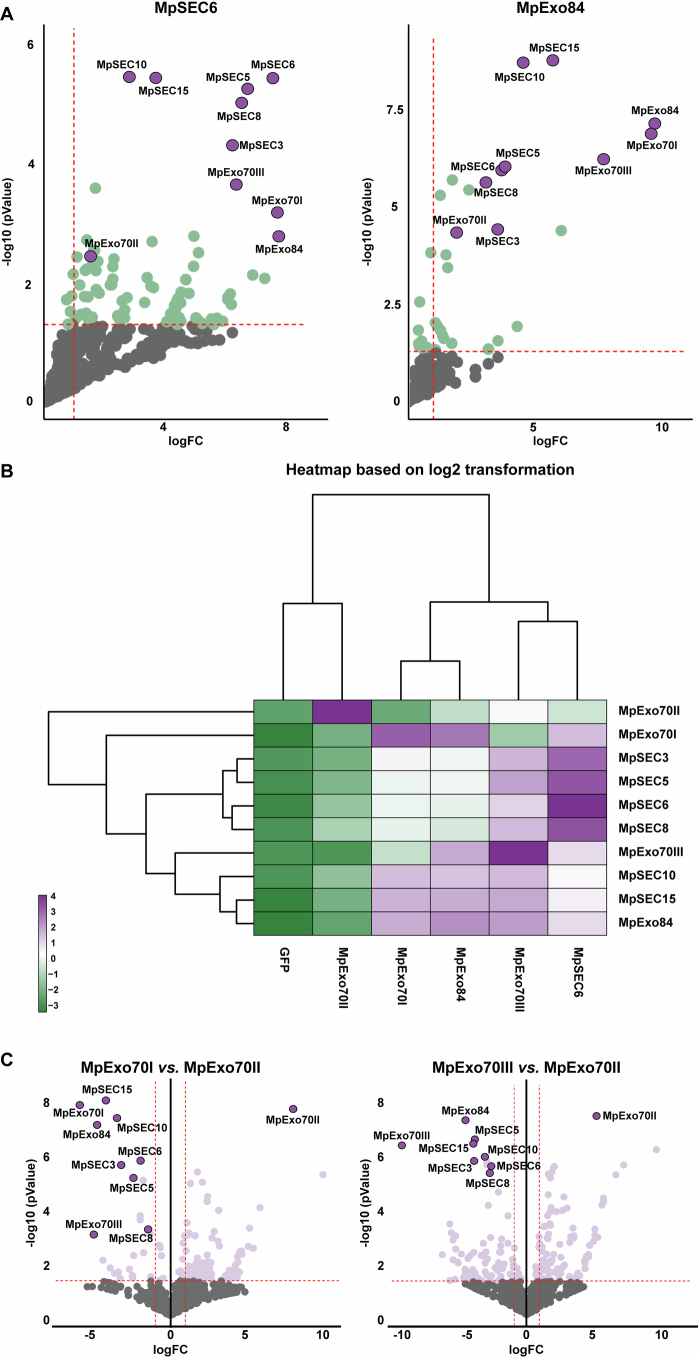
MpExo70II does not associate with the exocyst core components. **(A)** Enrichment of proteins co-purified with MpSEC6 and MpExo84 compared to a control expressing free GFP and represented by a volcano plot. The horizontal dashed line indicates the threshold above which proteins are enriched (p value < 0.05, quasi-likelihood negative binomial generalized log-linear model) and the vertical dashed line the threshold for which proteins log2 fold change (logFC) is above 1. For each plot, members of Marchantia exocyst complex are depicted by a purple dot with the corresponding name. **(B)** Protein abundance pattern represented by a heatmap (Log2(PSM + 1) – meanPSM per protein, with values clipped between -3.5 and 4) for the exocyst components in each IP-MS dataset. Results represented are the mean from three independent replicates. **(C)** Enrichment of proteins co-purified with MpExo70I and MpExo70III compared to MpExo70II and represented by a volcano plot. The horizontal dashed line indicates the threshold above which proteins are enriched (p value < 0.05, quasi-likelihood negative binomial generalized log-linear model) and the vertical dashed line the threshold for which proteins log2 fold (logFC) change is above 1. For each plot, members of Marchantia exocyst complex are depicted by a purple dot with the corresponding name.

To assess whether their divergent localizations reflect functional specialization, we compared the protein interactomes of Exo70 paralogues using immunoprecipitation coupled to mass spectrometry (IP–MS). IP–MS analysis of the three Exo70s, along with MpSec6 and MpExo84, revealed that MpExo70I specifically associates with core exocyst proteins, similar to MpSec6 and MpExo84. By contrast, MpExo70II and MpExo70III had broader interactomes with limited overlap (Fig. 1b, Supplementary Figs. 7–9 and Supplementary Datasets 1 and 2), indicating that the Exo70 paralogues may have functionally diversified through altered protein–protein interactions. Notably, whereas MpExo70I and MpExo70III are strongly associated with core exocyst subunits, these proteins were largely absent in the MpExo70II interactome (Fig. 1c and Extended Data Fig. 2). These data indicate that Exo70 paralogues not only have divergent interactomes but also suggest that MpExo70II may no longer associate with the exocyst at all.

**Extended Data Fig. 3:**
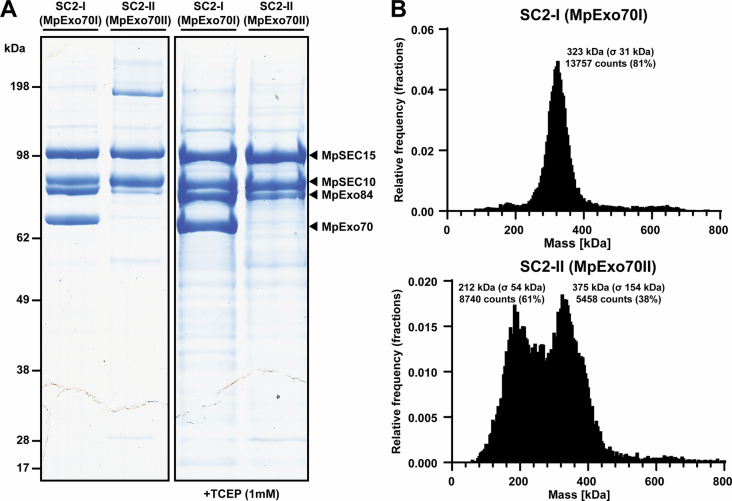
MpExo70II does not associate with exocyst proteins in purified subcomplexes. **(A)** SDS-PAGE analysis of purified Marchantia exocyst subcomplex IIa (MpSEC15:MpSEC10:MpExo84:MpExo70I) and exocyst subcomplex IIb (MpSEC15:MpSEC10:MpExo84:MpExo70II) produced in insect cells. The bands corresponding to MpSEC15:GFP:6XHis (MW: 118,7 kDa), MpSEC10 (92,2 kDa), MpExo84 (86,4 kDa) and MpExo70 (~73 kDa) are indicated by arrows **(B)** Mass distribution of purified exocyst subcomplex IIa (MW: 370 kDa) and exocyst subcomplex IIb (MW: 374,3 kDa) estimated by mass photometry. For each peak, an estimated mass, standard deviation and number of counts is indicated.

To further assess the dissociation between MpExo70II and the exocyst complex, we tested the interaction between each MpExo70 paralogue and MpExo84, the core component that hetero-dimerizes with Exo70 at the exocyst CorEx^37,38^. Indeed, a genome-wide yeast-two-hybrid (Y2H) screen confirmed MpExo84 as the primary interactor of MpExo70I (Supplementary Fig. 10). Similarly, pairwise Y2H analysis showed that Exo84 interacts with MpExo70I and MpExo70III, but not with MpExo70II. (Fig. 1d and Supplementary Fig. 11). Consistent with our Y2H and IP–MS results, in planta co-IP assays demonstrated that MpExo70II association with MpExo84 is reduced compared with MpExo70I and MpExo70III (Fig. 1e). To further test whether MpExo70II can integrate into the exocyst at all, we expressed and purified Marchantia exocyst subcomplex 2 containing MpSEC10, MpSEC15, MpExo84 and either MpExo70I (SC2-I) or MpExo70II (SC2-II) from insect cells^28,37,38^. Purified SC2-I had a homogeneous distribution containing all four subunits in stoichiometric amounts, whereas purified SC2-II seemed to lack MpExo70II, resulting in a heterogeneous protein population (Extended Data Fig. 3). Altogether, these results demonstrate that MpExo70II has subfunctionalized and no longer interacts with MpExo84 and the exocyst itself, indicating that this key exocyst subunit has dissociated from the ancestral complex.

## Exo70 paralogues have functionally diversified in Marchantia

**Fig. 2:**
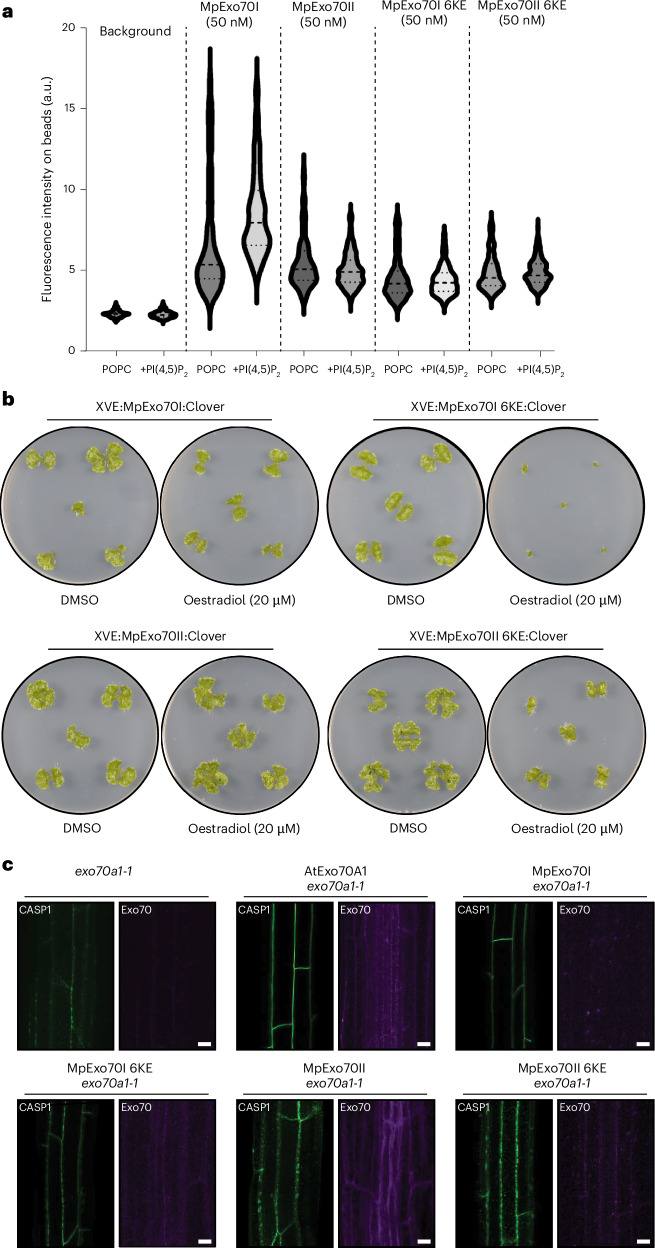
Marchantia Exo70 paralogues are functionally diverse. **a**, Evaluation of MpExo70 membrane binding at 50 nM. Fluorescence intensity of membrane-associated GFP:MpExo70I, GFP:MpExo70I 6KE, GFP:MpExo70II and GFP:MpExo70II 6KE was imaged by confocal microscopy. Representative confocal images are presented in Supplementary Fig. 13. Violin plots with median and quartiles are derived from individual beads. **b**, Macroscopic phenotypes of Marchantia transgenic lines XVE:MpExo70I:Clover, XVE:MpExo70I 6KE:Clover, XVE:MpExo70II:Clover and XVE:MpExo70II 6KE:Clover grown for 14 days on media containing β-oestradiol or dimethyl sulfoxide (DMSO). Images are representative of experimental replicates presented in Supplementary Fig. 14. **c**, CASP1:GFP accumulation at the Casparian strips of mature endodermis in *A. thaliana exo70a1-1*^47^ mutants lines (green) complemented with AtExo70A1, MpExo70I, MpExo70I 6KE, MpExo70II or MpExo70II 6KE (magenta). Dotted mis-localization of CASP1:GFP is representative of defect in Exo70 exocytic function^47^. Scale bar, 10 µm. Each experiment was repeated at least three times with similar results.

The canonical function of Exo70 is to recruit the exocyst to the plasma membrane. This function is achieved both through its interaction with the exocyst via Exo84 and through membrane targeting via phospholipid binding^28,48,59^. Given the dissociation between MpExo70II and the exocyst, we next sought to determine whether the Exo70 paralogues exhibit differential lipid binding affinities. Accordingly, we purified GFP:MpExo70I and GFP:MpExo70II from insect cells (Supplementary Fig. 12) and tested their ability to bind phospholipids by measuring their recruitment to liposomes^28^. The results showed that MpExo70I is preferentially recruited to liposomes containing phospholipids, while no preferential binding is observed for MpExo70II (Fig. 2a and Supplementary Fig. 13). These data suggest MpExo70 paralogues have functionally diverged in their ability to bind phospholipids.

**Extended Data Fig. 4:**
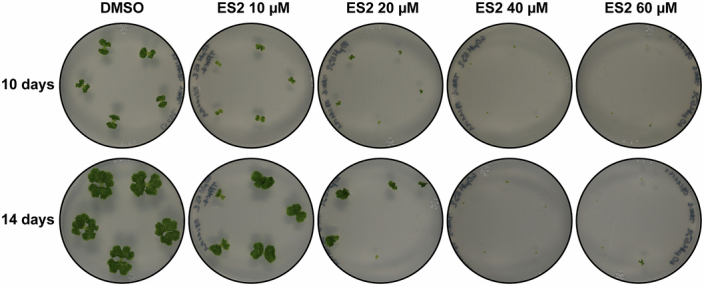
Endosidin 2 (ES2) inhibits Marchantia growth. Growth defect phenotypes of Marchantia Tak-1 grown for 10 or 14 days on media containing increasing concentrations of ES2 (0, 10, 20, 40 and 60 μM).

**Extended Data Fig. 5:**
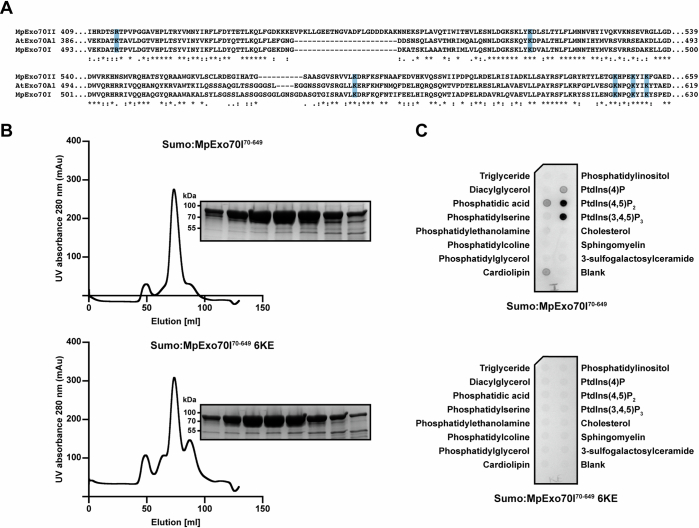
Lys to Glu (KE) mutations in MpExo70 abrogate binding to phospholipids. **(A)** Protein sequence alignment with Clustal Omega^108^ for AtExo70A1, MpExo70I and MpExo70II at the C-terminal region containing the lipid binding domain. Conserved residues involved in lipid binding previously described for AtExo70A1^48^ are highlighted in blue. **(B)** Elution trace of SUMO:MpExo70I^70–649^ and SUMO:MpExo70I^70–649^ KE (MW: 78,5 kDa) produced in *E. coli* after gel filtration and SDS-PAGE analysis of relevant elution peak. **(C)** Protein–lipid overlay assays with purified SUMO:MpExo70I^70–649^ and SUMO:MpExo70I^70–649^ KE. Bound proteins were visualized using anti-SUMO antisera. Each experiment was repeated at least three times with similar results.

Next, we sought to obtain genetic evidence for a possible functional divergence between MpExo70 paralogues. We could not generate a Marchantia CRISPR–Cas9 knockout for any of the paralogues, suggestive of an essential function. We validated this possibility using Endosidin 2 (ES2), an inhibitor that targets Exo70 and disrupts canonical exocytosis^60^. Similar to *A. thaliana*, ES2 treatment resulted in growth arrest, supporting the essentiality of Exo70-mediated exocytosis in Marchantia (Extended Data Fig. 4). To circumvent this bottleneck and investigate the importance of MpExo70I and MpExo70II, we first generated dominant negative mutants of both paralogues by replacing the conserved lysine residues (K) in the lipid-binding site with glutamic acid (E) to prevent phospholipid binding^48,59^ (Extended Data Fig. 5). Indeed, this mutation abrogated the binding of MpExo70I to liposomes (Fig. 2a). We then inducibly overexpressed these dominant negative mutants and assessed plant phenotypes. MpExo70I 6KE phenocopied ES2 treatment, leading to growth arrest (Fig. 2b and Supplementary Fig. 14). By contrast, expression of MpExo70II 6KE only mildly affected growth and instead led to extensive proliferation of rhizoids (Fig. 2b and Supplementary Fig. 14). These alternative outcomes suggest that MpExo70I and MpExo70II are functionally diversified.

To further assess the functional differences between MpExo70I and MpExo70II, we tested whether MpExo70 paralogues could complement exocytosis-related defects in an *A. thaliana exo70a1* mutant. AtExo70A1 is the orthologue of MpExo70I, and its disruption results in multiple phenotypes^48,49^, including defects in exocytosis-dependent deposition of AtCASP1 at the Casparian strip in *A. thaliana* roots^47^. Consistent with a role in canonical exocytosis, MpExo70I complemented CASP1–GFP deposition defects, and this complementation required a functional lipid-binding domain (Fig. 2c). On the contrary, neither MpExo70II nor MpExo70II 6KE could complement CASP1 deposition defects (Fig. 2c). Taken together, these results suggest that MpExo70I exhibits ancestral Exo70 activity, whereas MpExo70II has highly diversified to the point where it is not only dissociated from the exocyst but probably functions independently of exocytosis.

## The N-terminal domain of Exo70 determines its localization

**Fig. 3:**
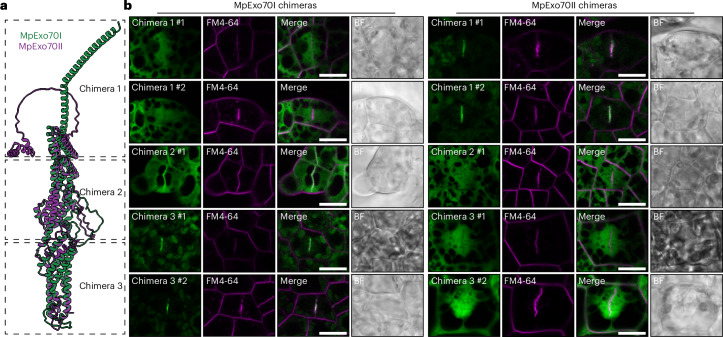
N-terminal domain determines Exo70 localization. **a**, Superposition of AlphaFold2^78,80^-predicted structures for MpExo70I (green) and MpExo70II (magenta) indicating the N-terminal, middle and C-terminal domains that are swapped to generate chimera 1 (residues Met1 to Ser154 in MpExo70I and Met1 to Ser169 in MpExo70II), chimera 2 (residues Lys155 to Asp397 in MpExo70I and Glu170 to Asp413 in MpExo70II) and chimera 3 (residues Ala398 to Arg649 in MpExo70I and Thr414 to Ser685 in MpExo70II), respectively. Both molecules were represented as cartoon ribbons using ChimeraX^107^. **b**, Confocal micrographs of Marchantia cells expressing C-terminally Clover-tagged chimeric MpExo70 proteins swapping domains between MpExo70I and MpExo70II (green) and stained with FM4-64 (magenta). The presence of the cell plate is depicted by accumulation of FM4-64 stain. Scale bar, 10 µm. Two independent transformants were analysed for each line, except for MpExo70I chimera 2 and MpExo70II chimera 2 for which only a single transformant was obtained. A gallery of images was collected to document variability in each line and is presented in Supplementary Figs. 15 and 16 for MpExo70I and MpExo70II chimeras, respectively. Each experiment was repeated at least three times with similar results.

**Extended Data Fig. 6:**
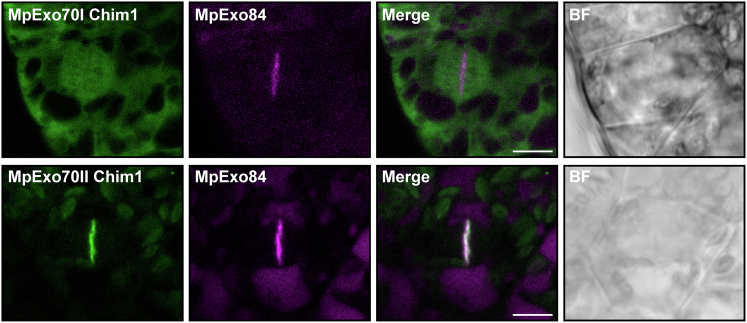
Co-localization of MpExo70 chimera 1 proteins with the exocyst complex. Confocal micrographs of *M. polymorpha* cells stably co-expressing MpExo70I chimera 1:Clover or MpExo70II chimera 1:Clover (green) and MpExo84:mScarlet (magenta). The presence of the cell plate is depicted by accumulation of MpExo84:mScarlet. Scale bar is 10 µm. BF indicates Bright Field. Each experiment was repeated at least three times with similar results.

Next, we sought to determine the molecular and structural features underlying MpExo70II diversification. First, we took advantage of the structural similarities between Exo70s^61^ and designed a series of chimeric proteins by swapping structurally equivalent regions between MpExo70I and MpExo70II (Fig. 3a). Live cell imaging analysis of Marchantia lines expressing chimeras revealed that swapping the N-terminal region between MpExo70I and MpExo70II (residues Met1 to Ser154 in MpExo70I and Met1 to Ser169 in MpExo70II) was sufficient to switch their localizations (Fig. 3b and Supplementary Figs. 15 and 16). In particular, MpExo70I with the N terminus of MpExo70II (MpExo70I chimera 1) had a diffuse localization pattern similar to MpExo70II, while MpExo70II with the N terminus of MpExo70I (MpExo70II chimera 1) accumulated at the cell plate and colocalized with MpExo84 (Extended Data Fig. 6). None of the other chimeras showed an altered localization compared with the original proteins (Fig. 3b and Supplementary Figs. 15 and 16). These data suggest that the N-terminal domain determines Exo70 localization.

## Variation at Exo70 N-terminal impacts exocyst association

**Fig. 4:**
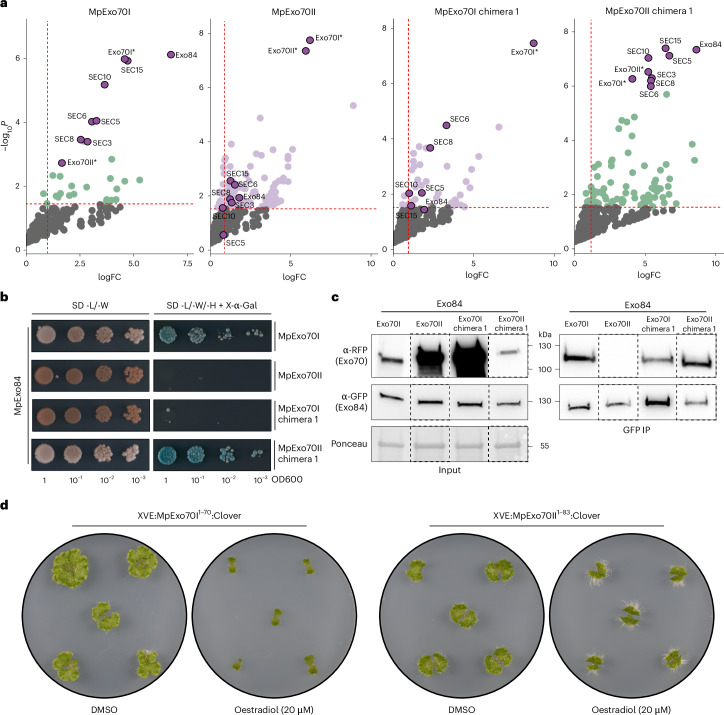
N-terminal domain determines Exo70 exocyst association. **a**, Enrichment of proteins copurified with MpExo70I chimera 1 and MpExo70II chimera 1 compared with a control expressing free GFP and represented by a volcano plot. The horizontal dashed line indicates the threshold above which proteins are enriched (*P* value <0.05, quasi-likelihood negative binomial generalized log-linear model), and the vertical dashed line indicates the threshold for which proteins’ log_2_FC is above 1. For each plot, members of Marchantia exocyst complex are depicted by a purple dot with the corresponding name. Wild-type MpExo70I and MpExo70II were included as controls, obtaining a result similar to what is presented in Fig. 1c. An asterisk next to the name has been added as a cautionary mark to indicate challenges in assigning the peptides to either a chimera or a wild-type Exo70. **b**, Y2H assay of MpExo70I chimera 1 and MpExo70II chimera 1 interaction with MpExo84. For each combination, 5 µl of yeast at indicated OD_600_ was spotted and incubated in double dropout plate (lacking L and W) for yeast growth control (left) and triple dropout media (lacking L, W and H) supplemented with X-α-gal (right). Growth and development of blue colouration on the right indicates protein–protein interactions. Wild-type MpExo70I and MpExo70II were included as controls. MpExo70s were fused to the GAL4 DNA-binding domain, while MpExo84 was fused to the GAL4 activator domain. Accumulation of each protein in yeast cells was tested by western blot and is presented in Supplementary Fig. 19. Each experiment was repeated at least three times with similar results. **c**, Co-IP of MpExo70I chimera 1 and MpExo70II chimera 1 with MpExo84. C-terminally mScarlet-tagged MpExo70 proteins were stably co-expressed with C-terminally Clover-tagged MpExo84 in Marchantia. IPs were obtained with anti-GFP magnetic beads, and total protein extracts were probed with anti-RFP or anti-GFP antibodies for MpExo70 and MpExo84 proteins, respectively. Wild-type MpExo70I and MpExo70II were included as controls. Dashed lines represent a cropped and assembled image from the same blot. Each experiment was repeated at least three times with similar results. **d**, Macroscopic phenotypes of Marchantia transgenic lines XVE:MpExo70I^1–70^:Clover and XVE:MpExo70II^1–83^:Clover grown for 14 days on media containing β-oestradiol or DMSO. Images are representative of experimental replicates presented in Supplementary Fig. 21. Source data

**Extended Data Fig. 7:**
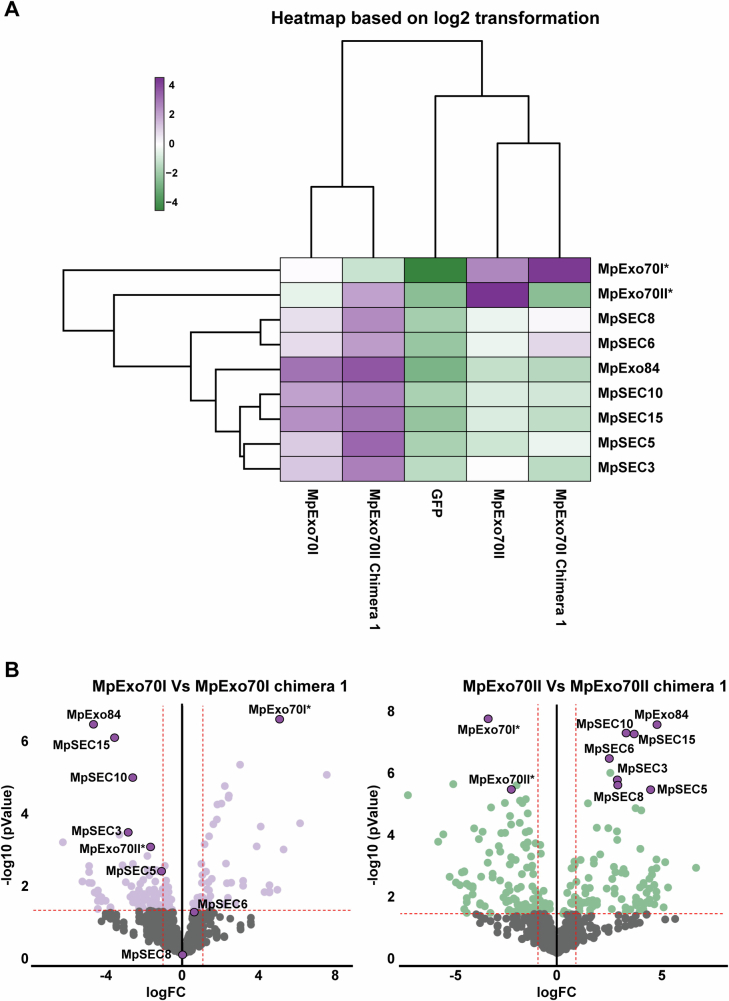
N-terminal domain swaps change MpExo70 association with the exocyst complex. **(A)** Protein abundance pattern represented by a heatmap (Log2(PSM + 1) – meanPSM per protein) for the exocyst components in each IP-MS dataset. Results represented are the mean from three independent replicates. Asterisk next to the name has been added as a cautionary mark indicating challenges in assigning the peptides to a chimera or a wild-type Exo70. **(B)** Enrichment of proteins co-purified with MpExo70I vs. MpExo70I chimera 1 and MpExo70II vs. MpExo70II chimera 1 represented by a volcano plot. The horizontal dashed line indicates the threshold above which proteins are enriched (p value < 0.05, quasi-likelihood negative binomial generalized log-linear model) and the vertical dashed line the threshold for which proteins log2 fold change (logFC) is above 1. For each plot, members of Marchantia exocyst complex are depicted by a purple dot with the corresponding name. Asterisk next to the name has been added as a cautionary mark indicating challenges in assigning the peptides to a chimera or a wild-type Exo70.

To determine whether the changes in localization also coincided with functional shifts, we investigated how N-terminal exchange altered the interactomes of MpExo70I and MpExo70II using IP–MS. Consistent with our live cell imaging results, IP–MS experiments showed that swapping the N-terminal helical bundle altered MpExo70I and MpExo70II interactomes (Supplementary Figs. 17 and 18 and Supplementary Dataset 3). Notably, swapping the N-terminal domain between MpExo70I and MpExo70II was sufficient to exchange their association with the exocyst complex (Fig. 4a and Extended Data Fig. 7), correlating with the change in cell plate localization. To determine whether the localization and interactome changes were linked to MpExo84 interaction, we also assessed Exo70 chimera and Exo84 interactions using Y2H, AlphaFold Multimer and co-IP assays. These experiments demonstrated that the N-terminal region of MpExo70I facilitates the association with MpExo84, while the MpExo70II N-terminus does not interact with MpExo84 in Y2H and reduces the association with MpExo84 in planta (Fig. 4b,c and Supplementary Figs. 19 and 20).

**Extended Data Fig. 8:**
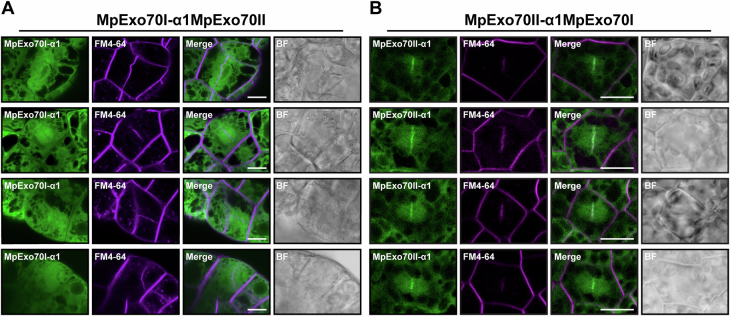
Localization of MpExo70 α1 chimeras in Marchantia. Micrograph collection of Marchantia cells stably expressing **(A)** MpExo70I-α1MpExo70II chimera:Clover and **(B)** MpExo70II-α1MpExo70I chimera:Clover stained with FM4-64 (magenta). The presence of the cell plate is depicted by accumulation of FM4-64 stain. Scale bar is 10 µm. BF indicates Bright Field. Each experiment was repeated at least three times with similar results.

Given the known importance of the N-terminal region in mediating the interaction between Exo70 and the exocyst complex, we further narrowed down the determinants for MpExo70 localization to the first predicted α-helix (residues Met1 to Leu70 in MpExo70I and Met1 to Leu83 in MpExo70II) (Extended Data Fig. 8). We then hypothesized that overexpression of this α-helix should outcompete native Exo70s, causing dominant negative phenotypes. In agreement with our hypothesis, inducible overexpression of MpExo70I^1–70^:Clover arrested growth in Marchantia (Fig. 4d and Supplementary Fig. 21), mimicking ES2 treatment and MpExo70I 6KE over-expression. By contrast, expression of MpExo70II^1–83^:Clover matched the phenotype of MpExo70II 6KE, causing only a mild growth defect (Fig. 4d and Supplementary Fig. 21). Altogether, these results suggest that exocyst dissociation and functional divergence between MpExo70I and MpExo70II are driven by alterations in the N-terminal α-helix.

## Electrostatic divergence in the Exo70 N terminus facilitated exocyst dissociation

**Fig. 5:**
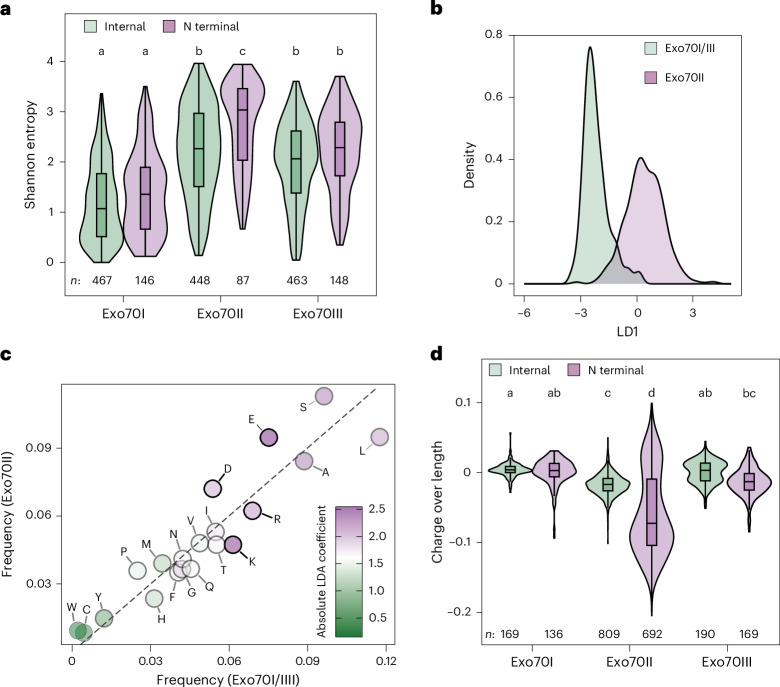
Electrostatic divergence in the Exo70 N terminus facilitated exocyst dissociation. **a**, Shannon entropy for aligned sites in the N-terminal helix and the remainder of the protein (internal) of land plant Exo70I, Exo70II and Exo70III paralogues. The centre line of the boxplots denotes the median and the upper and lower borders span from the first to the third quartiles, with whiskers extending 1.5 times the interquartile range. The ends of the whiskers denote the minima and maxima. Distributions were compared using two-sided pairwise Tukey honestly significant difference (HSD) tests. Significance groups are denoted using compact letter display (*P* < 0.01 after Bonferroni multiple test correction). Sample sizes (*n*) are noted beneath each boxplot. **b**, LDA differentiating Exo70 paralogues on the basis of N-terminal α-helix amino acid composition. **c**, Amino acid frequencies in the N-terminal α-helices of Exo70 paralogues. Points have been coloured based on their contribution to the LDA. **d**, Normalized electrostatic charge of the N-terminal α-helix compared with the rest of the protein for land plant Exo70 paralogues. Sample sizes are noted beneath each boxplot (*n*).

**Extended Data Fig. 9:**
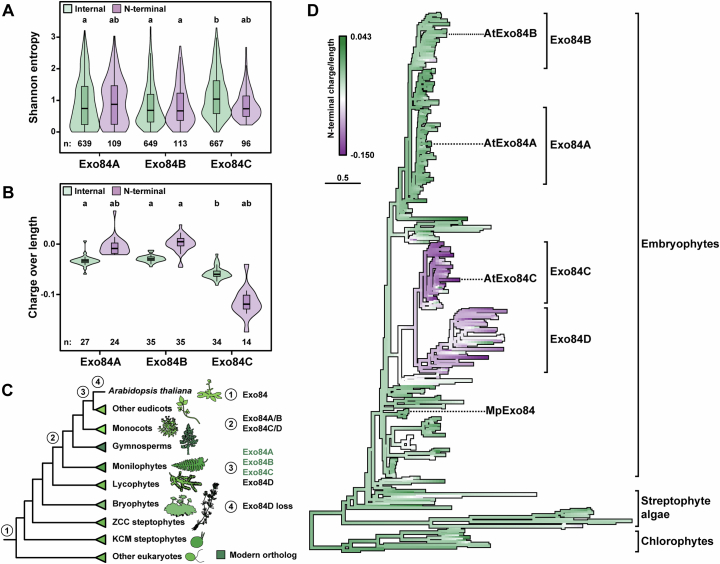
Electrostatic and evolutionary analysis of Exo84 N-terminal domain. **(A)** Shannon entropy for aligned sites (*n*) in the N-terminal α-helix and the remainder of the protein (internal) of eudicot Exo84A, Exo84B, and Exo84C paralogs. The center line of the boxplots denotes the median and the upper and lower borders span from the first to the third quartiles, with whiskers extending 1.5 times the interquartile range. The ends of the whiskers denote the minima and maxima. Distributions were compared using two-sided pairwise Tukey honestly significant difference (HSD) tests. Significance groups are denoted using compact letter display (P < 0.01 after Bonferroni multiple test correction). Sample sizes (*n*) are noted beneath each boxplot. **(B)** Normalized electrostatic charge of the N-terminal α-helix compared to the rest of the protein for eudicot Exo84 paralogs. Sample sizes (*n*) are noted beneath each boxplot. **(C)** Relative timing of the emergence of each of the eudicot Exo84 paralogs inferred using the Exo84 phylogeny in Supplementary information Fig. S1. Taxonomic groups are noted with cartoons obtained from Phylopic.org. **(D)** A maximum likelihood phylogeny of the Exo84 family overlaid with ancestral state reconstructions of the normalized electrostatic charge of the N-terminal α-helices. *Arabidopsis thaliana* and *Marchantia polymorpha* paralogs as well as Exo84 subfamilies have been noted. The scale bar represents the average number of substitutions per site. Full phylogenies are available in Supplementary information Fig. S1 and from iTOL^98^ (https://itol.embl.de/shared/OK75j4e8edHZ).

To understand how N-terminal variation impacted Exo70 diversification, we aimed to define the biochemical nature of the N-terminal domain in different Exo70 clades. To this end, we first identified Exo70 homologues from diverse land plants and assessed the amino acid conservation in Exo70I, Exo70II and Exo70III paralogues. In agreement with our experimental results, the N-terminal domain of Exo70II was significantly more diverse than the rest of the protein and both Exo70I and Exo70III (Fig. 5a). Given this diversity, we attempted to determine whether amino acid content could differentiate the Exo70 paralogues using linear discriminant analysis (LDA) (Fig. 5b). We found that amino acid content accurately predicted orthologue identity (accuracy ~95%), largely due to an increased abundance of negatively charged residues (D and E) and a decrease in positively charged residues (K and R) in the N-terminal domain of Exo70II compared with Exo70I and Exo70III (Fig. 5c). Accordingly, the average net charge of the Exo70II N-terminal domain was more negative than that of other Exo70s (Fig. 5d). Notably, we observed a similar phenomenon in the Exo84 paralogues of seed plants, where the N-terminal domain of Exo84c is more negatively charged relative to the N termini of Exo84a and Exo84b (Extended Data Fig. 9a,b). These results suggest that electrostatic tuning could impact the interactions between different Exo70 and Exo84 paralogues.

**Fig. 6:**
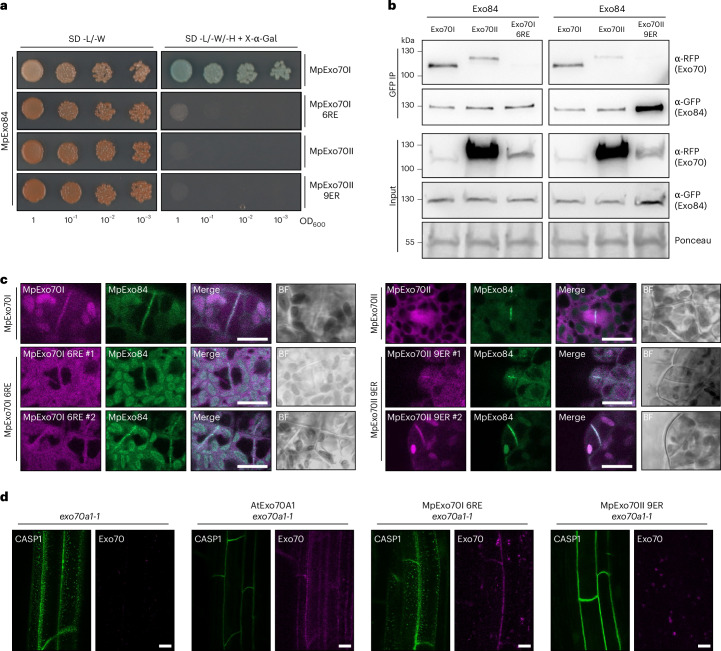
Electrostatic divergence in the Exo70 N terminus facilitated exocyst dissociation. **a**, Y2H assay of MpExo70I 6RE and MpExo70II 9ER interaction with MpExo84. For each combination, 5 µl of yeast at indicated OD_600_ was spotted and incubated in double dropout media (lacking L and W) for yeast growth control (left) and triple dropout media (lacking L, W and H) supplemented with X-α-gal (right). Growth and development of blue colouration on the right indicates protein–protein interactions. Wild-type MpExo70I and MpExo70II were included as controls. MpExo70s were fused to the GAL4 DNA-binding domain, while MpExo84 was fused to the GAL4 activator domain. Accumulation of each protein in yeast cells was tested by western blot and is presented in Supplementary Fig. 22. **b**, Co-IP of MpExo70I 6RE and MpExo70II 9ER with MpExo84. C-terminally mScarlet-tagged MpExo70 proteins were stably co-expressed with C-terminally Clover-tagged MpExo84 in Marchantia. IPs were obtained with anti-GFP magnetic beads and total protein extracts were probed with anti-RFP or anti-GFP antibodies for MpExo70 and MpExo84 proteins, respectively. Wild-type MpExo70I and MpExo70II were included as controls. Each experiment was repeated at least three times with similar results. **c**, Confocal micrographs of Marchantia cells from two independent cell lines stably co-expressing C-terminally tagged mScarlet MpExo70I 6RE or MpExo70II 9ER (magenta) with MpExo84:Clover (green). The presence of the cell plate is depicted by the accumulation of MpExo84. Wild-type MpExo70I and MpExo70II are included as controls. Scale bar, 10 µm. For each line, a gallery of images was collected to document variability and is presented in Supplementary Figs. 23 and 24 for MpExo70I 6RE and MpExo70II 9ER, respectively. Each experiment was repeated at least three times with similar results. **d**, CASP1:GFP accumulation at the Casparian strips of mature endodermis in *A. thaliana exo70a1-1*^47^ mutants lines (green) complemented with MpExo70I 6RE or MpExo70II 9ER (magenta). Dotted mis-localization of CASP1:GFP is representative of defect in Exo70 exocytic function^47^. Scale bar, 10 µm. Each experiment was repeated at least three times with similar results. Source data

**Extended Data Fig. 10:**
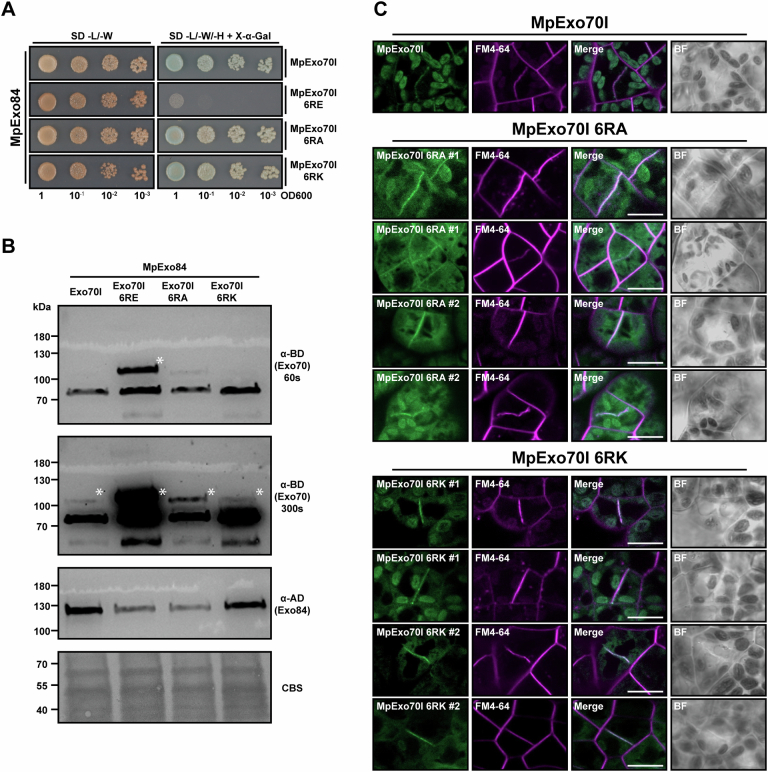
Substitution of MpExo70I Arg residues by Ala or Lys do not alter interaction with MpExo84 or cell plate localization. **(A)** Yeast two-hybrid assay of MpExo70I 6RA and MpExo70I 6RK interaction with MpExo84. For each combination, 5 µl of yeast at indicated OD_600_ were spotted and incubated in double dropout media (lacking Leucine (L) and Tryptophan (W)) for yeast growth control (left) and triple dropout media (lacking Leucine (L), Tryptophan (W) and Histidine (H)) supplemented with X-α-gal (right). Growth and development of blue coloration in the right panel indicates protein-protein interactions. Wild-type MpExo70I and MpExo70I 6RE were included as positive and negative controls, respectively. MpExo70s were fused to the GAL4 DNA binding domain while MpExo84 was fused to the GAL4 activator domain. Each experiment was repeated at least three times with similar results. **(B)** Accumulation of each protein in yeast cells was probed for the presence of MpExo70I, MpExo70I 6RE, MpExo70I 6RA and MpExo70I 6RK using anti-GAL4 binding domain (BD); and MpExo84 using anti-GAL4 DNA activation domain (AD) antibodies. Total protein extracts were stained with Coomassie Blue Stain (CBS). Accumulation of MpExo70I 6RE was consistently higher and two panels with indicated exposure times are included. Asterisks indicate the bands corresponding to the proteins of interest. **(C)** Micrograph collection of two independent Marchantia cell lines stably expressing MpExo70I 6RA:Clover or MpExo70I 6RK: Clover stained with FM4-64 (magenta). A micrograph with Wild-type MpExo70I:Clover is included as control. The presence of the cell plate is depicted by accumulation of FM4-64 stain. Scale bar is 10 µm. BF indicates Bright Field. Each experiment was repeated at least three times with similar results. Source data

To experimentally test whether the N-terminal charge is sufficient to control the association between the exocyst and Exo70I and Exo70II, we generated charge substitution mutants in which we replaced MpExo70I residues Arg13, Arg18, Arg41, Arg50, Arg55 and Arg60 with Glu (MpExo70I 6RE), recreating the charge of MpExo70II. Likewise, we changed the negative net charge of MpExo70II by replacing residues Glu7, Glu25, Glu30, Glu40, Glu47, Glu54, Glu60, Glu64 and Glu66 with Arg (MpExo70II 9ER). To assess the functional impact of charge inversion, we tested MpExo84 interaction and cell plate localization as a proxy for exocyst association. Charge inversion in MpExo70I was sufficient to abrogate the interaction with MpExo84 in Y2H (Fig. 6a and Supplementary Fig. 22) and co-IP assays (Fig. 6b). However, this inversion did not reconstitute MpExo70II binding to MpExo84 (Fig. 6a,b). Consistent with the loss of interaction with MpExo84, MpExo70I 6RE lost cell plate localization, adopting a diffuse localization reminiscent of MpExo70II (Fig. 6c and Supplementary Fig. 23). In the case of MpExo70II 9ER, although charge reversion was not sufficient to stably interact with MpExo84, it led to the partial relocalization of MpExo70II 9ER to the cell plate (Fig. 6c and Supplementary Fig. 24). To ensure the functional changes in MpExo70I 6RE were a result of charge reversion rather than alteration of specific residues, we also mutated MpExo70I Arg residues to Ala (MpExo70I 6RA) or Lys (MpExo70I 6RK). In contrast to MpExo70I 6RE, both MpExo70I 6RA and MpExo70I 6RK retained MpExo84 binding (Extended Data Fig. 10a,b) and cell plate localization (Extended Data Fig. 10c). Finally, we tested whether these electrostatic reversion mutants impact complementation of Exo70A1 loss of function in *A. thaliana*. In agreement with the loss of MpExo84 interaction and delocalization, MpExo70I 6RE could not complement the CASP1 deposition phenotype at the Casparian strip (Fig. 6d). Interestingly, MpExo70II 9ER complemented CASP1 deposition in nine out of ten plants tested, reminiscent of the partial colocalization of MpExo70II 9ER with the exocyst complex (Fig. 6d). Taken together, our results suggest that electrostatic change in the Exo70 N-terminal domain, mediated by a few amino acid substitutions, underpins the functional divergence of Exo70 paralogues.

## An electrostatic shift in the N-terminal domain predates the expansion of Exo70II

**Fig. 7:**
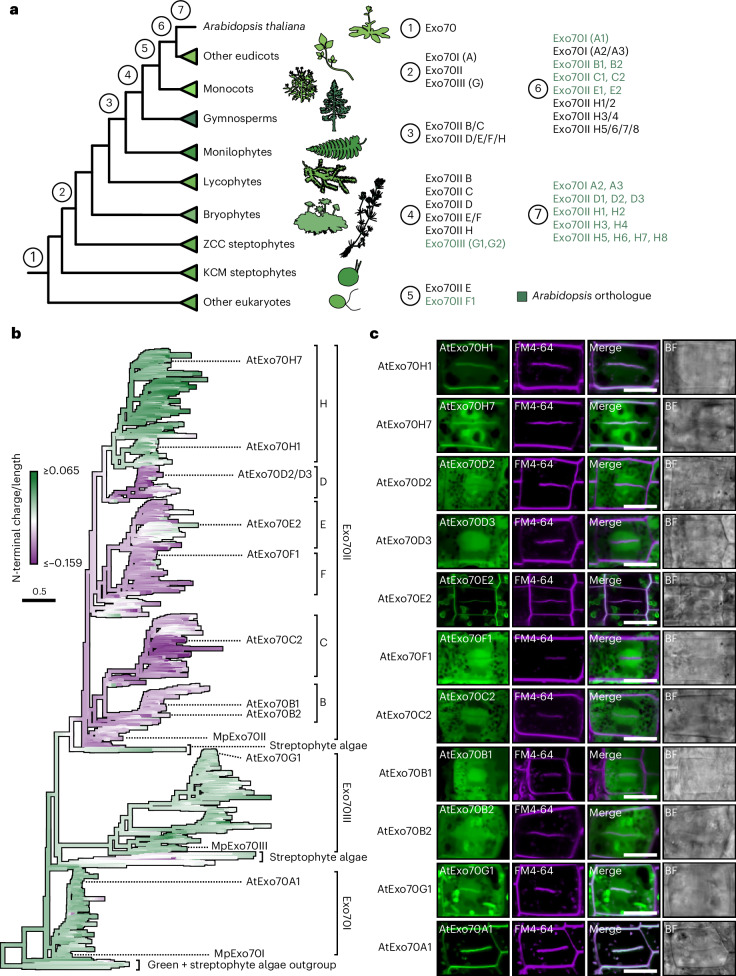
An electrostatic shift in the N-terminal domain predates the expansion of Exo70II. **a**, Relative timing of the emergence of each of the *A. thaliana* Exo70 paralogues inferred using the Exo70 phylogeny presented in Supplementary Fig. 1. Taxonomic groups are noted with cartoons obtained from Phylopic.org. **b**, A maximum likelihood phylogeny of the Exo70 family overlaid with ancestral state reconstructions of the normalized electrostatic charge of the N-terminal α-helices. *A. thaliana* and *M. polymorpha* paralogues used in this study, as well as Exo70 subfamilies, have been noted. Subfamily classification was based on the phylogeny in Supplementary Fig. 1 and assigns both gymnosperm and angiosperm paralogues when possible. The scale bar represents the average number of substitutions per site. Full phylogenies are available in Supplementary Fig. 1 and from iTOL^98,99^. **c**, Confocal micrographs of *A. thaliana* root cells stably expressing C-terminally GFP tagged AtExo70 proteins (green) stained with FM4-64 (magenta). The presence of the cell plate is depicted by the accumulation of FM4-64 stain. Scale bar, 10 µm. Two additional images were collected to document variability in each line and are presented in Supplementary Fig. 25.

Given the association between Exo70 function and N-terminal electrostatics, we hypothesized that N-terminal charge alterations may have enabled exocyst dissociation, leading to Exo70 diversification. Our comprehensive phylogenetic analysis of the Exo70 family in plants (Supplementary information Fig. 1) confirmed that the three Exo70 families (I, II and III) arose early in the streptophyte lineage, and in contrast to Exo70I and III, Exo70II radiated extensively in vascular plants (Fig. 7a, Supplementary Fig. 1 and Supplementary Dataset 4), as suggested previously^40,42^. Based on this phylogenetic context, we examined the evolutionary history of Exo70 N-terminal charge using ancestral state reconstruction. This analysis revealed that the ancestral Exo70 probably harboured an uncharged N terminus, which was retained in the Exo70I and Exo70III families. However, the emergence of Exo70II coincided with a negative electrostatic shift in the N terminus that predated the Exo70II radiation in vascular plants (Fig. 7b). Exo84 showed a similar pattern where N-terminal charge diversification coincided with the emergence of Exo84c and Exo84d in seed plants (Extended Data Fig. 9c,d). Interestingly, during the diversification of Exo70II, N termini in subclades E and H reverted to a neutral or positive charge. Based on these data, we hypothesized that N-terminal charge variation would correlate with exocyst association. To assess this, we generated a collection of *A. thaliana* lines overexpressing representative members of Exo70 subclades A to H and assessed their localization at the cell plate (Fig. 7c and Supplementary Fig. 25). Similar to Marchantia, *A. thaliana* Exo70s from clades I and III with non-negative N-terminal domains (subclades A and G, respectively), localized at the cell plate, consistent with exocyst association. On the contrary, Exo70II paralogues with a predicted negative charge (subclades B, C, D and F) had diffuse localizations reminiscent of Marchantia MpExo70II. However, Exo70II paralogues exhibiting charge reversion such as AtExo70E2, AtExo70H1 and AtExo70H7 all localized to the cell plate (Fig. 7c and Supplementary Fig. 25). Overall, our data indicate that electrostatic tuning impacted Exo70 evolution by enabling the reversible dissociation from the exocyst.

## Discussion and conclusion

**Fig. 8:**
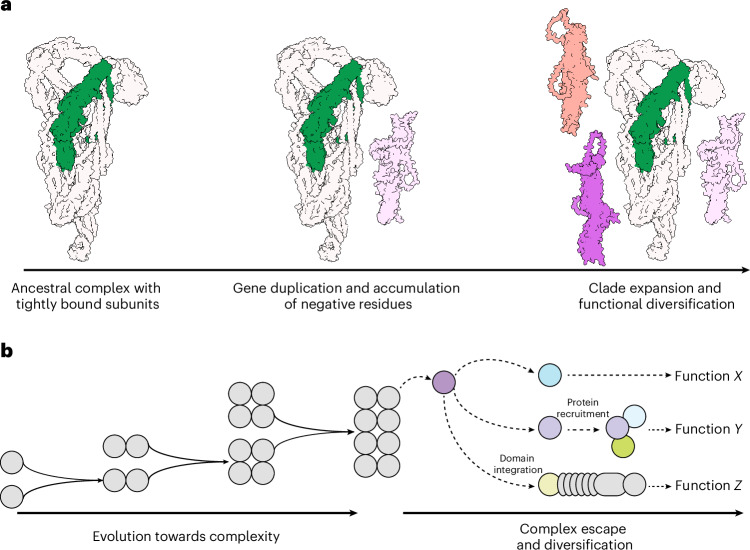
Graphical summary of plant Exo70 diversification by protein complex escape. **a**, From left to right: the exocyst is a heteromeric complex composed of tightly bound subunits required to perform its function. Gene duplication and accumulation of negative residues at the N-terminal domain led to dissociation of the Exo70 subunit. Dissociation from the exocyst complex relieved paralogue interference and enabled Exo70 expansion and functional diversification. **b**, Protein complexes can evolve towards complexity by gene duplication and neofunctionalization. In a protein complex escape scenario, a single subunit dissociates from the complex to perform a novel function (function *X*), to be recruited by molecular interactions with other proteins (function *Y*) or to get integrated as a domain within other proteins (function *Z*).

As most of the protein machinery governing essential cellular processes is thought to have been present in the last common eukaryotic ancestor^62^, understanding the diversification of core eukaryotic machinery is a fundamental challenge in evolutionary cell biology. In this study, we reveal how a core subunit of the exocyst complex, Exo70, diversified in plants following its dissociation from the complex. This separation was driven by electrostatic changes in the N-terminal region of Exo70, which are crucial for its interaction with Exo84 and subsequent association with the exocyst. We hypothesize that disengagement from the exocyst alleviated evolutionary constraints typically associated with integration into hetero-multimeric complexes, permitting functional diversification. This evolutionary flexibility was probably exploited during vascular plant evolution and led to the remarkable diversification of the Exo70 family observed today (Fig. 8). Notably, complex escape appears to have not only permitted the neofunctionalization of Exo70 but may also have enabled the indirect diversification of the exocyst, as certain Exo70 paralogues seem to have dissociated from the complex, diversified and subsequently reintegrated. Understanding the activity of individual Exo70 paralogues, the functional contributions of their altered N- and C-terminal regions, and their relationships to the exocyst and core subunits such as Exo84 represents a key direction for future research.

Although dissociation from the exocyst could have permitted neofunctionalization, integration into alternative cellular systems was probably required for Exo70 diversification. This was reflected in the alternative interactomes of MpExo70I and MpExo70II, which could be reversed through N-terminal exchange. This suggests that, following exocyst escape, novel interactions emerged, resulting in the recruitment of Exo70 into diverse exocytosis-independent pathways. Whether the potential for these novel interactions was present in the Exo70 ancestor but masked by strong exocyst association, or emerged de novo following dissociation, is still unclear. However, Exo70s have been reported to acquire short linear motifs that enable interactions with the core autophagy protein ATG8^63–65^ and are involved in pathogen recognition in monocots^53,54,61,66^. These functions may not depend on exocytosis. Consistent with this, some members of the Exo70 F clade have lost the entire N-terminal domain, presumably precluding association with the exocyst^54,67^. In *Arabidopsis*, pollen-specific AtExo70C2 was also reported to lack interaction with other exocyst subunits^68^. Collectively, these examples warrant caution when inferring function from homology and underscore the need to assess paralogue-specific activities.

The functional flexibility of Exo70 also sheds light on the evolution of protein subunits. Previous work primarily focusing on homomeric complexes, elucidated how proteins evolve into multimeric assemblies^4,8,10,26^, partly through the formation of hydrophobic interfaces^6,8,9^ leading to molecular entrenchment^5,7,10^. These models suggest that integration into multimeric complexes should result in evolutionary stasis. However, our work reveals an alternative mechanism in which simple substitutions enable dissociation even from ancestral complexes. The extent to which ‘protein complex escape’ occurs in other systems probably depends on specific factors, such as the ease of subunit dissociation, the potential to form new interactions and the ability to function as an independent module. We hypothesize that further research may uncover overlooked cases of subunit duplication in other complexes, which could reveal unexpected instances of protein diversification, expanding the functional repertoire of the core eukaryotic proteome.

## Methods

### Molecular cloning

For protein production in *Escherichia coli*, MpExo70I and MpExo70I 6KE spanning amino acids 70 to 649 were amplified by PCR and subsequently cloned into the pOPIN-GG vector pPGN-C^69^ with an N-terminal 6xHis:SUMO tag via Golden Gate cloning^70^. For insect cells, GFP:MpExo70I^99–649^ and GFP:MpExo70II^111–685^ and their corresponding mutants were synthesized as codon-optimized DNA fragments and cloned via GoldenBac^71^ by the ProTech service at Vienna BioCenter. Marchantia exocyst subcomplex IIa (MpSEC15:MpSEC10:MpExo84:MpExo70I) and exocyst subcomplex IIb (MpSEC15:MpSEC10:MpExo84:MpExo70II) were synthesized as codon-optimized full-length coding sequences and cloned for co-expression by GoldenBac.

For Y2H assays, coding sequences for MpExo70 proteins, mutants and MpExo84 were synthesized as double-stranded DNA fragments (Twist Bioscience) and cloned in pGADT7 (MpExo84) or pGBKT7 (MpExo70) vectors adapted for Golden Gate cloning^70^ provided by the SynBio service at The Sainsbury Laboratory, Norwich.

For stable transformation in Marchantia and *A. thaliana*, coding sequences were assembled with the indicated promoter, fluorescence tag and terminators into pGGsun via GreenGate cloning^72^. β-Oestradiol-inducible expression constructs were generated by Gateway cloning^73^ into pMpGWB168 (XVE::GW)^74,75^.

### Plant growth and transformation

#### *Marchantia polymorpha*

Marchantia Tak-1 was axenically maintained and asexually propagated on 0.5 Gamborg B5 + MES medium (1.5 g l^−1^ B5 Gamborg, 0.5 g l^−1^ MES hydrate, 1% sucrose, pH adjusted to 5.5) containing 1% (w/v) agar at 21 °C under continuous white light (50 µM m^−2^ s^−1^).

Stable transformants of Marchantia were generated by gemmae transformation. Tak-1 gemmae were grown for 2 days in 0.5 Gamborg B5 + MES medium plates containing 1% (w/v) agar. A solution of *Agrobacterium tumefaciens* strain GV3101 containing the construct resuspended in transformation media (1.5 g l^−1^ B5 Gamborg, 0.5 g l^−1^ MES hydrate, 1 g l^−1^ casein hydrolysate, 0.3 g l^−1^ of L-glutamine, 2% sucrose, pH adjusted to 5.5) supplemented with 150 µM acetosyringone was added to the plates and incubated at 28 °C in dark. After incubation, Marchantia gemmae were washed with sterile water, scraped from the transformation plate, and transferred to 0.5× Gamborg B5 + MES plates containing ticarcillin and the selective antibiotic. Transformants were recovered from the plates after 2–4 weeks.

#### *Arabidopsis thaliana*

*A. thaliana* lines used in this study were sown on water-saturated soil for standard plant growth under a 16 h light/8 h dark photoperiod with 165 µM m^−2^ s^−1^ light intensity. For in vitro seedling growth, *Arabidopsis* seeds were surface sterilized in 70% ethanol 0.05% sodium dodecyl sulfate (SDS) for 15 min, rinsed in ethanol absolute and dried on sterile paper. Seeds were plated in ½ Murashige–Skoog salts (Duchefa), 1% sucrose containing 1% (w/v) agar and stratified for 48 h at 4 °C in dark. Plates were then grown under light-emitting diodes with 50 µM m^−2^ s^−1^ and a 16-h light/8-h dark photoperiod.

Stable transformants of *A. thaliana* were generated by delivering the desired constructs via plant transformation with *Agrobacterium tumefaciens* strain GV3101 using the floral dip method^76^.

A list of all plant lines generated here can be found in Supplementary Table 1.

### Confocal microscopy

For imaging of Marchantia cells, asexual gemmae were incubated in liquid 0.5 Gamborg B5 + MES medium for 2 days at 21 °C under continuous white light (50 µM m^−2^ s^−1^). The thalli were then placed on a microscope slide with deionized water and covered with a coverslip. The meristem region was used for image acquisition. For colocalization of Marchantia exocyst proteins with FM4-64, plants were treated for 10 min with 13.3 μM FM4-64 before imaging.

For confocal microscopy of *A. thaliana* seedling roots, seed sterilization was performed using Sterilization Solution I (70% ethanol, 0.01% Triton X-100) for 10 min followed by Sterilization Solution II (50% sodium hypochlorite) for 5 min. Removal of Sterilization Solution II was done rinsing the seeds four times with Milli-Q water. Seeds were sown on Murashige–Skoog medium right after sterilization. Stratification was carried out by incubating the plates at 4 °C in the dark for 3 days, after which they were transferred to a controlled-environment growth chamber and incubated at 22 °C under long-day photoperiod conditions (16-h-light/8-h-dark photoperiod) at 80–100 µM m^−2^ s^−1^. Plates were placed vertically to let the roots elongate along the media surface.

Three-day-old *Arabidopsis* seedlings were treated for 5 min with 13.3 μM FM4-64, placed on a microscope slide with water and covered with a coverslip. The epidermal cells of root meristem zone were used for AtExo70 and FM4-64 colocalization imaging. For imaging of the *Arabidopsis* Casparian strip, we imaged the tenth root cell above the elongation region.

Confocal imaging was performed using a downright point laser scanning confocal microscope Zeiss LSM 800 AxioImager.Z2 (Carl Zeiss) equipped with plan-Apochromat 63× corrective water immersion objective and ZEN software (blue edition, Carl Zeiss). FM 4-64 fluorescence was excited at 561 nm and detected between 656 nm and 700 nm. GFP fluorescence was excited at 488 nm and detected between 488 nm and 545 nm. mScarlet fluorescence was excited at 561 nm and detected between 570 nm and 617 nm. Pinholes were adjusted to one Airy Unit for each wavelength. For each experiment, all replicate images were acquired using identical confocal microscopic parameters. Confocal images were processed with Fiji (version 1.52, Fiji).

### Chemical treatments in Marchantia

Chemical treatments with Endosisin2 (ES2)^60^ or β-oestradiol were performed by culturing Marchantia gemmae directly into 0.5 Gamborg B5 + MES medium containing 1% (w/v) agar supplemented with different concentrations of ES2 or 20 μM β-oestradiol, respectively.

### Expression and purification of proteins for in vitro studies

#### Expression and purification of MpExo70s in *E. coli*

SUMO:MpExo70I^70–649^ or SUMO:MpExo70I^70–649^ 6KE were produced in *E. coli* Rosetta^TM^ (DE3). Cell cultures were grown in Terrific Broth at 37 °C for 5–7 h and then at 16 °C overnight after induction with 1 mM isopropyl β-D-1-thiogalactopyranoside. Cells were collected by centrifugation and resuspended in 20 mM HEPES pH 8, 500 mM NaCl, 5% (vol/vol) glycerol and 20 mM imidazole supplemented with cOmplete EDTA-free protease inhibitor tablets (Roche). Cells were sonicated, and, following centrifugation at 40,000*g* for 30 min, the clarified lysate was applied to a HisTrap Ni2 + -NTA column connected to an ÄKTA pure protein purification system (Cytiva). Proteins were step-eluted with elution buffer (20 mM HEPES pH 8, 500 mM NaCl, 5% (vol/vol) glycerol and 500 mM imidazole).

Fraction with the eluted proteins were collected and injected onto a HiLoad 16/600 Superdex 200 pg gel filtration column (Cytiva) preequilibrated with 20 mM HEPES pH 7.5, 150 mM NaCl and 5% (vol/vol) glycerol supplemented with 1 mM TCEP (Sigma). Elution fractions were collected and evaluated by SDS–polyacrylamide gel electrophoresis (PAGE), and relevant fractions with purified proteins were concentrated as appropriate for further studies.

#### Expression and purification of MpExo70s in insect cells

Source plasmids containing the desired constructs were transformed into DH10EMBacY cells. Colonies containing recombinant baculoviral shuttle vectors (bacmids) were selected by blue–white selection on LB agar plates containing X-Gal and isopropyl β-D-1-thiogalactopyranoside. Bacmid DNA was extracted by alkaline lysis and isopropanol precipitation and confirmed by PCR. To generate the viral stock (V0), bacmids were then transfected into adherent *Spodoptera frugiperda* (Sf9) insect cells in six-well plates, using transfection medium (Expression Systems) and polyethylenimine. Successful transfection was tracked by monitoring a yellow fluorescent protein encoded by the bacmid backbone. The plate was incubated for 1 week, and the virus was collected for amplification.

Recombinant protein was produced in *Trichoplusia ni* High Five cells (Thermo Fisher) infected at a density of 1× 10^6^ ml with the appropriate virus, and grown at 21 °C and 120 rpm. Cells were collected 4 days after infection by centrifugation at 1,000*g* for 15 min, and pellets were stored at −70 °C. All insect cell culture works were performed using ESF 921 serum-free growth medium (Expression Systems) without antibiotic supplementation.

Cell pellets were resuspended in 20 mM HEPES pH 8, 300 mM NaCl, 5% (vol/vol) glycerol and 20 mM imidazole supplemented with cOmplete EDTA-free protease inhibitor tablets (Roche) and Benzonase (IMP Molecular Biology Service). Resuspended cells were then disrupted using a glass Dounce homogenizer. After centrifugation at 40,000*g* for 30 min, the clarified lysate was processed as described above for protein purification.

#### Expression and purification of Marchantia exocyst subcomplex II

For expression and production of Marchantia exocyst subcomplex IIa (MpSEC15:MpSEC10:MpExo84:MpExo70I) and exocyst subcomplex IIb (MpSEC15:MpSEC10:MpExo84:MpExo70II), source plasmids containing the four proteins were transformed into DH10EMBacY cells. Viral stocks and *Trichoplusia ni* High Five cells expressing the subcomplexes were produced as described above.

Once collected, the pellets were resuspended in 20 mM HEPES pH 8, 150 mM NaCl and 20 mM imidazole supplemented with cOmplete EDTA-free protease inhibitor tablets (Roche) and Benzonase (IMP Molecular Biology Service). Resuspended cells were then disrupted using a glass Dounce homogenizer. After centrifugation at 40,000*g* for 30 min, the clarified lysate was applied to a HisTrap Ni2 + -NTA column connected to an ÄKTA pure protein purification system (Cytiva). Proteins were step-eluted with elution buffer (20 mM HEPES pH 8, 150 mM NaCl and 500 mM imidazole).

Fractions with the eluted proteins were collected and injected onto a HiLoad 16/600 Superose 6 pg preparative gel filtration column (Cytiva) preequilibrated with 20 mM HEPES pH 7.5, 150 mM NaCl and supplemented with 1 mM TCEP. Elution fractions were collected and evaluated by SDS–PAGE. Relevant fractions with purified proteins were then concentrated as appropriate.

### Analysis of purified proteins by mass photometry

Mass photometry analysis of proteins was performed at room temperature on a OneMP photometer instrument (Refeyn) with the data acquisition software AcquireMP (Refeyn). High-precision cover glasses (24 × 50 mm, VWR) were rinsed thoroughly with isopropanol, cleared using double-distilled water and dried under a clean nitrogen stream. One drop of type F immersion oil (Olympus) was applied to the photometer lens, and a cleaned cover glass was settled on the lens. A gasket (Grace Bio-Labs) was then adhered to the cover glass, and 9 μl of the buffer was pipetted into the gasket wall without touching the cover glass. The buffer drop was focused in a 5 μm × 10 μm field to a stable sharpness value around 5.5 before gently pipetting 1 μl of diluted protein sample (~0.1 μM) into the drop. The counting events were recorded for 60 s at a 1 kHz frame rate, and images were processed using DiscoverMP (Refeyn).

### Membrane overlay assays for studying protein–lipid interaction

Membrane lipid strips (Echelon Bioscience, P-6002) were blocked with 10 ml blocking buffer (5% bovine serum albumin, 10 mM Tris–HCl pH 7.5, 150 mM NaCl and 0.1% Tween 20) at room temperature for 1 h in gentle agitation. After incubation, 5 ml of the blocking buffer was exchanged with 5 ml of blocking buffer containing ~130 nM concentration of purified SUMO:MpExo70I^70–649^ or SUMO:MpExo70I^70–649^ KE. Strips were incubated at room temperature for 2 h under gentle agitation and then washed three times for 15 min with TBST (10 mM Tris–HCl pH 7.5, 150 mM NaCl and 0.1% Tween 20). A solution of SUMO-tag monoclonal antibody (4G11E9, Thermo Fisher Scientific) at 1:1,000 dilution in blocking buffer was added to the lipid strips and incubated 1 h at room temperature. The membranes were then washed with TBST again three times for 15 min before adding goat anti-mouse IgG (H + L)-HRP antibody (1706516, Bio-Rad) 1:10,000 in blocking buffer. After 1 h incubation, membranes were washed again three times with TBST, and images were captured using iBright Imaging System (Invitrogen) with SuperSignal West Pico PLUS Chemiluminescent Substrate (Thermo Fisher Scientific).

### Membrane-coated bead binding assay for studying protein–lipid interaction

Liposomes containing PI(4,5)P_2_ were made by mixing 85 mol% 1-palmitoyl-2-oleoyl-glycero-3-phosphocholine (POPC, Avanti Polar Lipids), 10 mol% phosphatidylserine (POPS, Avanti Polar Lipids), 5 mol% PI(4,5)P_2_ (Echelon Biosciences) and 0.1% Atto647N-DOPE (ATTO-TEC). Control liposomes without phosphoinositides were made by mixing 90 mol% POPC, 10 mol% POPS and 0.1% Atto647N-DOPE. The mixtures were then evaporated under a clean nitrogen stream and dried overnight under vacuum. Dried lipids were resuspended in 20 mM HEPES, 150 mM NaCl and 0.5 mM TCEP (Sigma) at 37 °C. The mixtures were then freeze-thawed for six cycles in liquid nitrogen and extruded to 0.1 μm via track-etched membranes (Whatman). The homogenized liposomes were ready to use, aliquoted, frozen in liquid nitrogen and stored at −20 °C.

Membrane-coated beads were made in 100 μl reactions by incubating 100 μM liposomes and 5 × 10^5^ silica beads (Whitehouse Scientific) in 250 mM NaCl on a rotator at room temperature for 30 min. Beads were then washed twice with 20 mM HEPES, pH 7.4, and resuspended in 20 mM HEPES, pH 7.4, 250 mM NaCl and 0.5 mM TCEP. Membrane coated beads were kept rotating while the binding assay was set up.

The protein–membrane binding assay was set up by mixing 7 μl of membrane-coated beads with 7 μl of protein samples at gradient concentrations in uncoated μ-Slide 8-well chambers (Ibidi)

### Y2H assay for studying protein–protein interaction

pGADT7 plasmid encoding MpExo84 was cotransformed into chemically competent Y2HGold cells (Takara Bio) with a pGBKT7 plasmid encoding MpExo70 alleles and mutants using the Frozen-EZ Yeast Transformation Kit (Zymo Research).

After growing in selection plates, single cotransformants were inoculated in liquid SD -Leu -Trp medium for 2 days at 30 °C. Saturated culture was then used to make serial dilutions of OD_600_ 1, 0.1, 0.01 and 0.001, and 5 µl of each dilution was spotted on a SD -Leu -Trp plate as a growth control, and on a SD -Leu -Trp -His plate containing X-α-gal (Takara Bio). Plates were imaged after incubation for 72 h at 30 °C.

To assay the accumulation of MpExo84 and MpExo70 proteins in yeast cells, total yeast extracts were produced by collecting cells from the liquid media and incubating for 10 min at 95 °C after resuspending them in Laemmli buffer. Samples were then centrifugated, and the supernatant was subjected to SDS–PAGE and western blot. The membranes were probed with anti-GAL4 DNA-binding domain (Sigma) antibody for the MpExo70s proteins in pGBKT7 and with the anti-GAL4 activation domain (Sigma) antibody for MpExo84 in pGADT7.

### In planta co-IP for studying protein–protein interaction

Marchantia gemmae from 10 gemmae cups were placed in 40 ml of liquid 0.5 Gamborg B5 + MES medium and incubated at 21 °C under continuous white light (50 µM m^−2^ s^−1^). Plant tissue was then collected and briefly dried on paper before grinding to a fine powder in liquid nitrogen using a pestle and mortar. The plant powder was mixed with ice-cold extraction buffer at a 1:2 (w/v) ratio (10% glycerol, 25 mM Tris pH 7.5, 1 mM EDTA, 150 mM NaCl, 2% w/v polyvinylpolypyrrolidone, 10 mM dithiothreitol, one cOmplete EDTA-free Protease Inhibitor Cocktail tablet (Roche) and 0.1% Tween 20 (Sigma) per 50 ml), centrifuged at 4,200*g* at 4 °C for 20–30 min, and the supernatant was passed through a 0.45-μm Minisart syringe filter. The presence of proteins in the input was determined by SDS–PAGE and western blot and probing the membranes with anti-GFP (11814460001/54732800, Roche) or anti-RFP (AB_2631395, Chromotek) antibodies for protein tagged with Clover and mScarlet, respectively.

For IP, 1 ml of filtered plant extract was incubated with 30 μl of GFP-Trap Magnetic beads (Chromotek) in a rotatory mixer at 4 °C. After 1 h, the beads were pelleted on a magnetic rack and the supernatant removed. The pellet was then washed and resuspended in 1 ml of IP buffer (10% glycerol, 25 mM Tris pH 7.5, 1 mM EDTA, 150 mM NaCl and 0.1% Tween 20 (Sigma)) and pelleted again as before. Washing steps were repeated five times. Finally, 30 μl of Laemmli buffer was added to the agarose and incubated for 10 min at 70 °C. The beads were pelleted again by centrifugation, and the supernatant was loaded on SDS–PAGE gels before western blotting. Membranes were probed with anti-GFP antibody (11814460001/54732800, Roche) to detect MpExo84:Clover and anti-RFP (AB_2631395, Chromotek) to detect MpExo70:mScarlet proteins.

### AP–MS for studying protein–protein interaction

#### AP

For affinity purification (AP) of Marchantia exocyst proteins, stable lines overexpressing MpSEC6, MpExo84 or MpExo70s with a C-terminal Clover tag were grown and processed for total protein extraction as detailed above.

IP of the proteins was performed by adding 30 μl of GFP-Trap magnetic beads (Chromotek) into 1 ml of crude extract and incubating in LoBind Eppendorf tubes for 1 h in a rotatory mixer at 4 °C. The beads were washed twice with IP buffer (10% glycerol, 25 mM Tris pH 7.5, 1 mM EDTA, 150 mM NaCl and 0.1% Tween 20 (Sigma)) by pelleting them on a magnetic rack and removing the supernatant. The beads were then washed four more times with IP buffer without Tween 20. After washing, the magnetic beads were resuspended in 100 µl of IP buffer without Tween 20. Then, 10 µl was drained and denatured in 20 µl of Laemmli buffer for quality check by western blot. The remaining 90 µl was pelleted by centrifugation, and after removing the excess of buffer, the tubes were stored at −20 °C before on-bead trypsin digestion.

#### Trypsin digestion

Beads were resuspended in 40 µl of 100 mM ammonium bicarbonate, supplemented with 400 ng of lysyl endopeptidase (Lys-C, Fujifilm Wako Pure Chemical Corporation), and incubated for 4 h at 37 °C on a Thermo-shaker set to 1,200 rpm. The supernatant was transferred to a fresh tube and reduced with 0.5 mM Tris 2-carboxyethyl phosphine hydrochloride (TCEP, Sigma) for 30 min at 60 °C and alkylated in 4 mM methyl methanethiosulfonate (Fluka) for 30 min at room temperature. Subsequently, the sample was digested with 400 ng trypsin (Trypsin Gold, Promega) at 37 °C overnight. The digest was acidified by addition of trifluoroacetic acid (Pierce) to 1%. A similar aliquot of each sample was analysed by liquid chromatography–MS/MS.

#### MS data acquisition

The nano HPLC system (UltiMate 3000 RSLC nano system) was coupled to an Orbitrap Exploris 480 mass spectrometer equipped with a Nanospray Flex ion source (all parts from Thermo Fisher Scientific).

Peptides were loaded onto a trap column (PepMap Acclaim C18, 5 mm × 300 μm, 5 μm particles, 100 Å pore size; Thermo Fisher Scientific) at a flow rate of 25 μl min^−1^ using 0.1% trifluoroacetic acid as the mobile phase. After loading, the trap column was switched in line with the analytical column (PepMap Acclaim C18, 500 mm × 75 μm ID, 2 μm particles, 100 Å pore size; Thermo Fisher Scientific). Peptides were eluted using a flow rate of 230 nl min^−1^, starting with the mobile phases 98% A (0.1% formic acid in water) and 2% B (80% acetonitrile, 0.1% formic acid) and linearly increasing to 35% B over the next 120 min. This was followed by a steep gradient to 95% B over 5 min, maintained for 5 min, then ramped down in 2 min to the starting conditions of 98% A and 2% B for equilibration at 30 °C.

The Orbitrap Exploris 480 mass spectrometer was operated in data-dependent mode, performing a full scan (*m*/*z* range 350–1,200, resolution 60,000, normalized automatic gain control target 300%) at three different compensation voltages (CVs −45 V, −60 V and −75 V), followed by MS/MS scans of the most abundant ions for a cycle time of 0.9 s for CVs −45 V and −60 V, and 0.7 s for CV −75 V. MS/MS spectra were acquired using an isolation width of 1.2 *m*/*z*, normalized automatic gain control target 200%, minimum intensity set to 25,000, higher-energy collision dissociation energy of 30%, maximum injection time of 100 ms and resolution of 30,000. Precursor ions selected for fragmentation (charge states 2–6) were excluded for 45 s. The monoisotopic precursor selection (MIPS) mode was set to peptide, and the exclude isotopes feature was enabled.

#### Analysis of MS results

The total number of MS/MS fragmentation spectra was used to quantify each protein (Supplementary Datasets 1 and 3). The data matrix of Peptide-Spectrum Match (PSM) was analysed using the R package IPinquiry4 (https://github.com/hzuber67/IPinquiry4) that calculates log_2_fold change (FC) and *P* values using the quasi-likelihood negative binomial generalized loglinear model implemented in the edgeR package^77^. Only proteins identified with at least three PSM were considered. Each sample was triplicated per experiment. Unspecific binding to the GFP tag and beads was accounted for by pairwise comparison to the GFP (empty vector) control, using a cut-off of log_2_FC >1 and *P* value <0.01.

#### AlphaFold2 Multimer for studying protein–protein interaction

We used AlphaFold2 Multimer^78–80^ to predict protein–protein interaction between Exo70s and candidate proteins as described in refs. ^81,82^. Protein sequences were extracted in FASTA format from marchantia.info and processed using mmseqs^83^ to generate local multiple sequence alignments. Subsequent structural predictions were performed with ColabFold^80^. The confidence in protein–protein interaction predictions was assessed using two metrics: the Interface Predicted TM-score (ipTM) and a custom PEAK score. The PEAK score represents the predicted aligned error between chains, excluding intramolecular interactions. For each interaction, five independent models were generated by AlphaFold2 using default settings. The average or maximum ipTM and PEAK scores from these models were used in the analysis.

#### Comparative genomics and phylogenetics for bioinformatic analyses

Exo70 and Exo84 paralogues were identified across eukaryotes using a diverse eukaryotic dataset comprised of genome-predicted proteomes obtained from UniProt (*n* = 174, downloaded 15 March 2023)^84^. To taxonomically balance the dataset, we selected the best two proteomes per genus based on BUSCO (Benchmarking Universal Single Copy Orthologues) completeness^85^. For metazoans, fungi, and embryophytes, stricter taxonomic criteria were applied by selecting the best proteome per phylum for metazoans (*n* = 17), the best proteome per class for fungi (up to two per phylum, *n* = 8) and the best proteome per order for embryophytes (up to three per class, *n* = 12). The dataset was also supplemented with transcriptome-predicted proteomes from two species of CRuMs (*Rigifila ramosa*: SRR5997435, *Diphylleia rotans*: SRR5997435). Each individual proteome was then clustered at 100% sequence identity using CD-HIT v4.8.1, and the resulting proteomes were combined and compiled into a searchable database using Diamond v2.0.9^86,87^.

To identify Exo70 homologues, the database was searched using Diamond BLASTp with *A. thaliana* Exo70A1 (UniProt accession: Q9LZD3) and Exo84A (UniProt accession: F4I4B6) as initial queries (query coverage ≥50%, *E* < 10^−5^, sensitive mode). The resulting hits were aligned using MAFFT v7.520, trimmed using trimAl v1.4.rev15 with a gap threshold of 50%, and a maximum-likelihood phylogeny was inferred with IQ-Tree v2.2.6 using the LG4M substitution model with topology support assessed using Shimodaira–Hasegawa approximate likelihood ratio tests (SH-aLRT, *n* = 1,000)^88–91^. The phylogenies were inspected manually in FigTree v1.4^92^, and non-homologous sequences were excluded. This search was repeated twice iteratively, and the resulting homologues were aligned using the L-INS-i algorithm of MAFFT and used to generate a profile hidden Markov model (HMM). The proteomes were then searched a final time with the HMM using HMMER v3.4 (*E* < 10^−5^), and the hits were screened phylogenetically as described^93^. Lastly, to check for proteins that were missed due to genomic mis-annotation, proteins identified from the predicted proteomes were used as queries for tBLASTn (*E* < 10^−5^) searches against eukaryotic genomes and downloaded from the National Center for Biotechnology Information, and protein predictions were generated using Exonerate v2.2 (https://github.com/nickatirwin/Phylogenomic-analysis)^94,95^. The final set of Exo70 and Exo84 homologues was finally aligned and used to generate an HMM.

To expand the identification of Exo70 and Exo84 homologues in Viridiplantae, a second dataset of predicted proteomes from plants (*n* = 81), streptophyte algae (*n* = 13) and chlorophytes (*n* = 21) was assembled from multiple sources, including UniProt and EukProt v3^96^. The resulting dataset was surveyed as described above, using two iterative rounds of HMMER searches. Lastly, to remove non-homologous sites, the HMMs were mapped to each protein using HMMER HMMScan (*E* < 10^−5^, domE <10^−5^) and homologous regions were extracted. These regions were aligned and trimmed with a gap threshold of 50%, and sequences with less than 50% (Exo70) or 25% (Exo84) trimmed alignment coverage were excluded. The final phylogenies were generated with IQ-Tree using the LG + C50 + F + R10 (Exo70) and LG + C50 + F + R7 (Exo84) substitution models, selected using ModelFinder^97^. Phylogenies were visualized in IToL v6^98^^,99^. Raw data with datasets, sequences, alignments and phylogenies can be found in Supplementary Dataset 4.

#### Sequence analysis and ancestral state reconstruction for bioinformatic analyses

To characterize N termini of Exo70 and Exo84, the secondary structure of each viridiplantae homologue was predicted using PSSPred v4^100^. Amino acid sequences were then recoded with secondary structure predictions and were subsequently aligned using MAFFT. The alignments were visualized using AliView v1.28^101^, and the N termini were identified and extracted (untrimmed sites: 0 to 9,870 for Exo70, and 0 to 2,906 for Exo84). The N-terminal and C-terminal regions were then recoded back to amino acids, clustered at 95% identity using CD-Hit and aligned using MAFFT L-INS-i. Plant Exo70 (I, II and III) and Exo84 (A, B and C) paralogues were then separated, and the individual alignments were trimmed with a gap threshold of 75% using trimAl. Shannon entropy was calculated for each site using the bio3D package in R v4.3.2^102^. LDA was conducted using the LDA implementation in the MASS package in R, based on amino acid composition of Exo70 N-terminal sequences. Model training was conducted using 30% of the data. To analyse electrostatics, charge at pH 7 was predicted using BioPython (Bio.SeqUtils) on N-terminal helices more than 100 amino acids in length^103^. Lastly, ancestral state reconstructions were conducted using the fastAnc function in PhyTools v2 based on the rooted Exo70 and Exo84 phylogenies and the length-normalized N-terminal charge of each sequence^104^. Raw data for the N-terminal analysis can be found in Supplementary Dataset 4.

### Reporting summary

Further information on research design is available in the Nature Portfolio Reporting Summary linked to this article.

## Supplementary information

Supplementary Information Supplementary Figs. 1–25.

Reporting Summary

Supplementary Table 1 Plant lines generated in this study.

Supplementary Dataset 1

Supplementary Dataset 2

Supplementary Dataset 3

Supplementary Dataset 4

## Source data

Source Data Figs. 1, 4, 6 and Extended Data Fig. 10 Unprocessed western blots.

## Data Availability

The MS proteomics data have been deposited to the ProteomeXchange Consortium via the PRIDE partner repository^105^ with the dataset identifier PXD051853. Source data are provided with this paper. These data and raw images used to generate Figs. 1–8, Extended Data Figs. 1–10 and Supplementary Figs. 1–25 are available via Zenodo at 10.5281/zenodo.13374122 (ref. ^106^).
