# Supplementary material for: Electrostatic changes enabled the diversification of an exocyst subunit via protein complex escape
Source: Nat Plants. 2025 Oct 31;11(11):2350–67. doi: 10.1038/s41477-025-02135-1 (PMC12626893; doi:10.1038/s41477-025-02135-1)
Supplement: Supplementary file 1 — Supplementary Figs. 1–25. [file 41477_2025_2135_MOESM1_ESM.pdf]

nature plants

Supplementary information https://doi.org/10.1038/s41477-025-02135-1

Electrostatic changes enabled the diversification of an exocyst subunit via protein complex escape

In the format provided by the authors and unedited

Supplemental Information

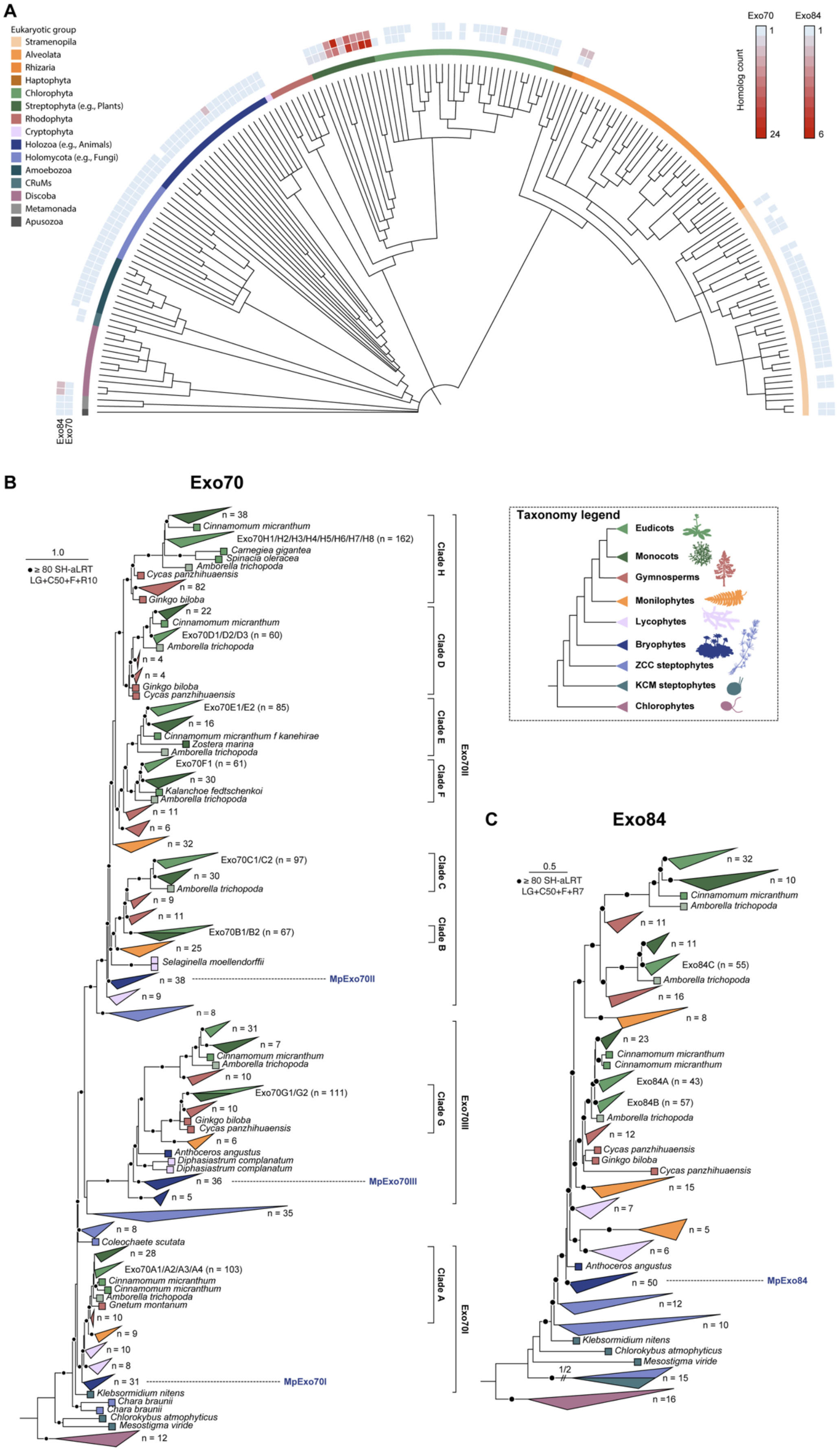

Fig. S1. (A) A eukaryotic phylogeny depicting the frequency and abundance of Exo70 and Exo84 paralogs across representative species. Eukaryotic supergroups have been denoted with a colored ribbon. **(B, C)** Maximum likelihood phylogenies of Exo70 **(A)** and Exo84 **(B)** in plants and green algae. Statistical support was inferred using ultrafast bootstrap (UFB) and Shimodaira-Hasegawa approximate likelihood ratio tests (SH-aLRT). The scale bars represent the average number of substitutions per site and clades have been colored taxonomically for clarity. Full phylogenies are available from iTOL (https://itol.embl.de/shared/OK75j4e8edHZ).

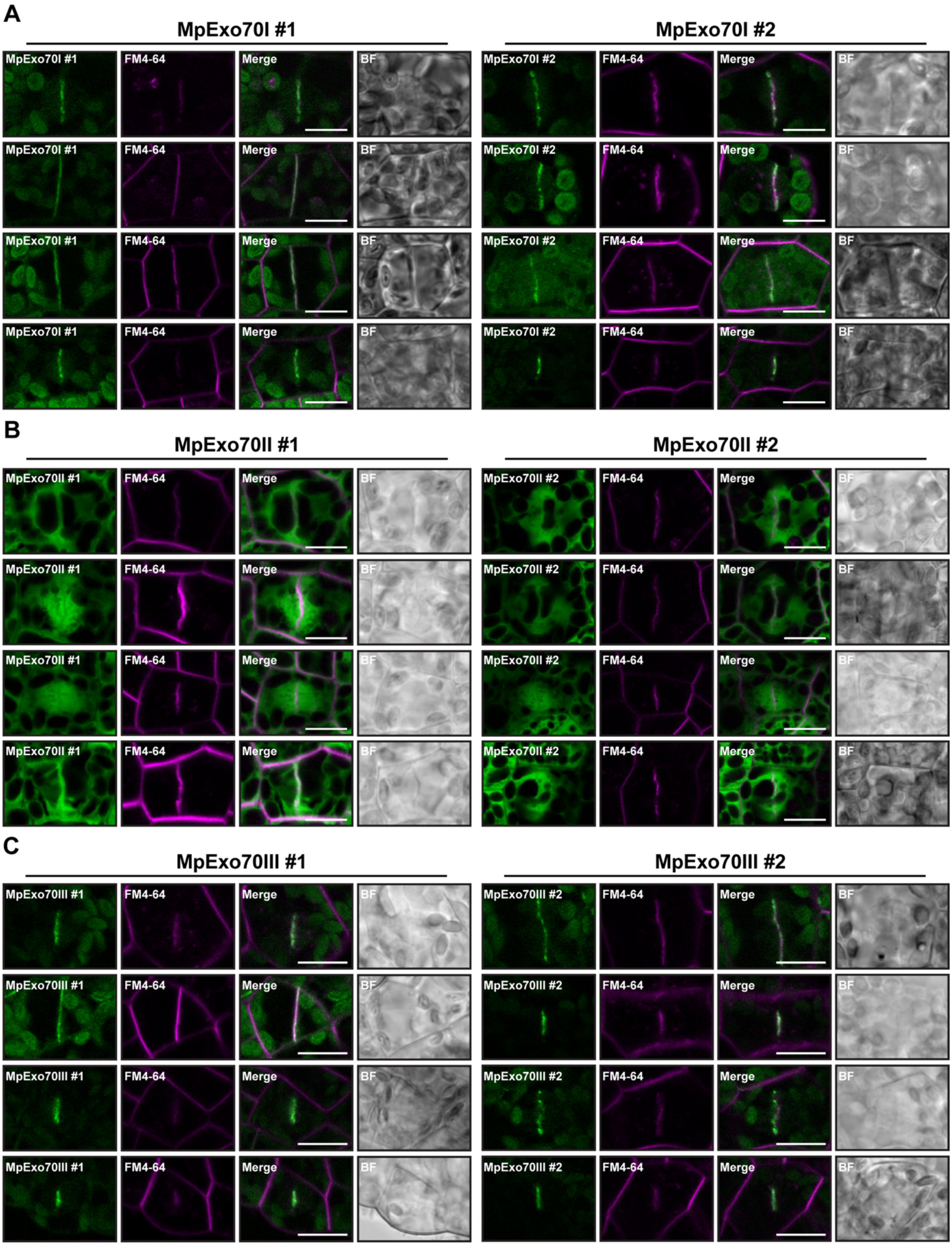
A
MpExo70I #1
MpExo70I #2
MpExo70I #1
MpExo70I #2
FM4-64
Merge
BF
B
MpExo70II #1
MpExo70II #2
C
MpExo70III #1
MpExo70III #2

Fig. S2. Image gallery for MpExo70 localization in Marchantia. Micrograph collection of Marchantia cells from two independent lines stably expressing **(A)** MpExo70I:Clover, **(B)** MpExo70II:Clover and **(C)** MpExo70III:Clover stained with FM4-64 (magenta). The presence of the cell plate is depicted by accumulation of FM4-64 stain. Scale bar is 10 µm. BF indicates Bright Field.

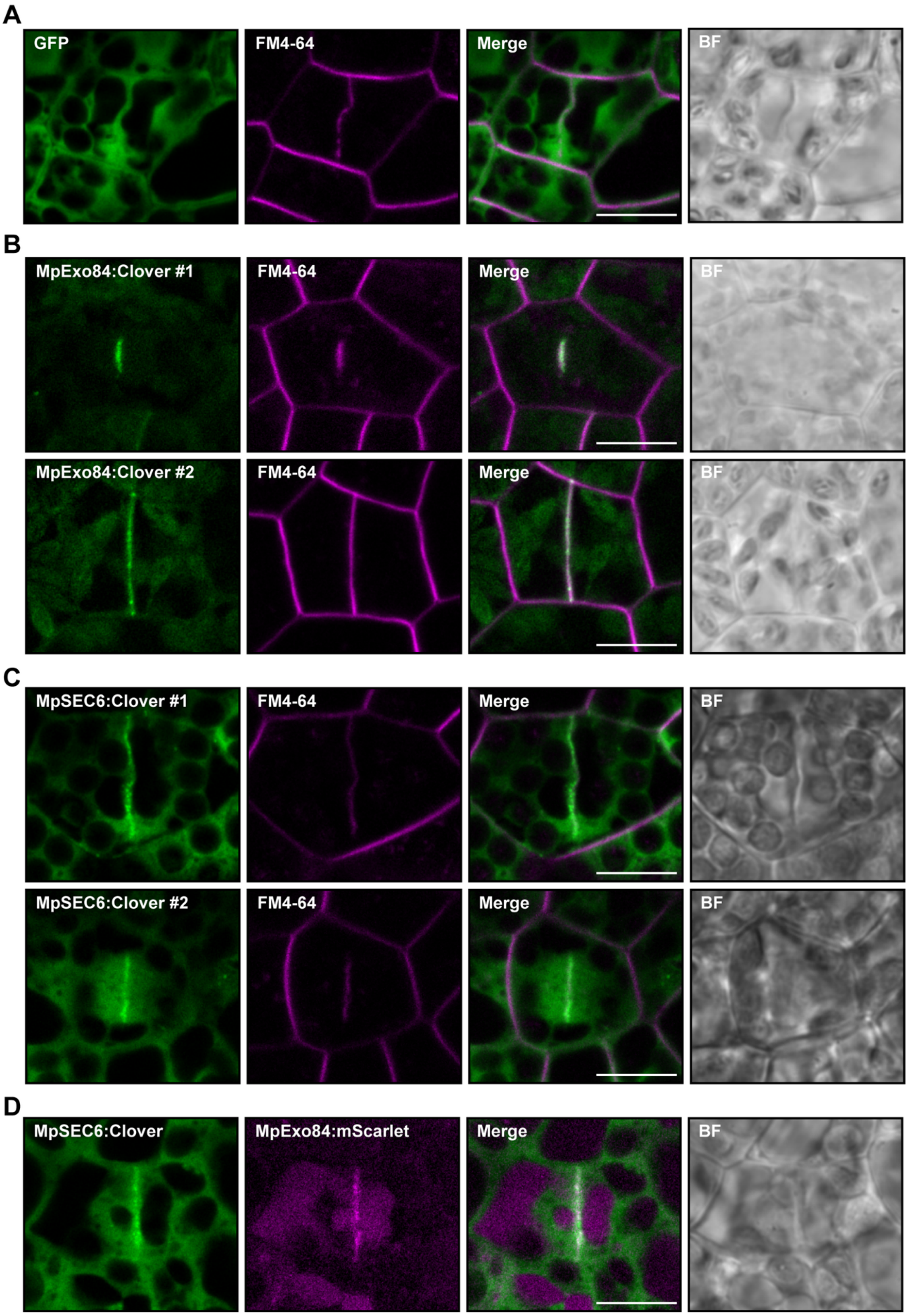
A
GFP
FM4-64
Merge
BF
B
MpExo84:Clover #1
FM4-64
Merge
BF
MpExo84:Clover #2
FM4-64
Merge
BF
C
MpSEC6:Clover #1
FM4-64
Merge
BF
MpSEC6:Clover #2
FM4-64
Merge
BF
D
MpSEC6:Clover
MpExo84:mScarlet
Merge
BF

Fig. S3. Marchantia exocyst localizes at the cell plate. Confocal micrographs of Marchantia cells stably expressing **(A)** free GFP, **(B)** MpExo84:Clover (two independent lines) and **(C)** MpSEC6:Clover (two independent lines) stained with FM4-64 (magenta). The presence of the cell plate is depicted by accumulation of FM4-64 stain. **(D)** Confocal micrographs of Marchantia cells stably co-expressing MpSEC6: Clover (green) or MpExo84:mScarlet (magenta). Scale bar is 10 μm. BF indicates Bright Field.

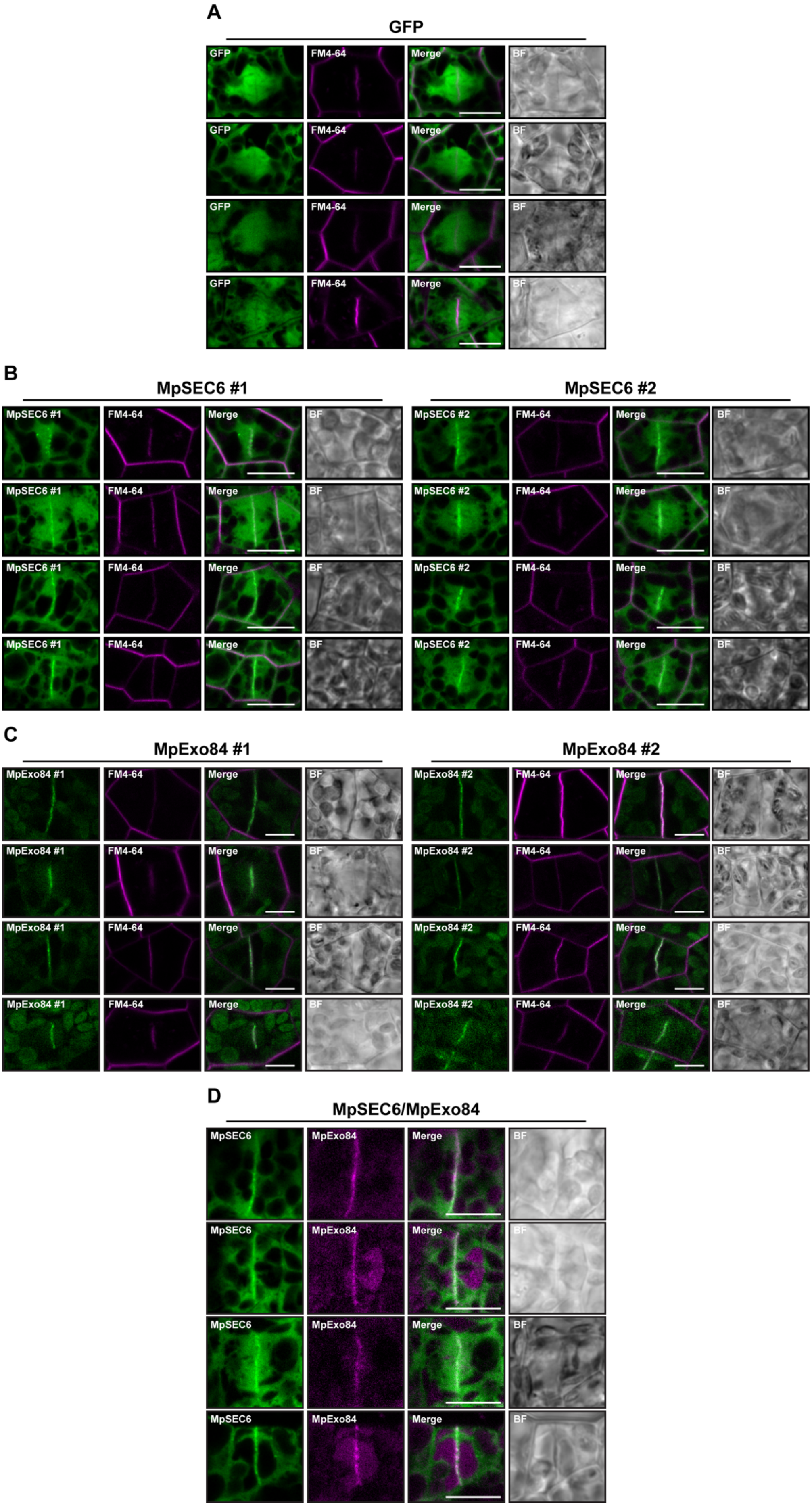
A
GFP
GFP
FM4-64
Merge
BF
B
MpSEC6 #1
MpSEC6 #2
FM4-64
Merge
BF
C
MpExo84 #1
MpExo84 #2
FM4-64
Merge
BF
D
MpSEC6/MpExo84
MpSEC6
MpExo84
Merge
BF

Fig. S4. Image gallery for MpExo84 and MpSEC6 localization in Marchantia. Micrograph collection of Marchantia cells stably expressing **(A)** free GFP, **(B)** MpExo84:Clover (two independent lines) and **(C)** MpSEC6:Clover (two independent lines) stained with FM4-64 (magenta). The presence of the cell plate is depicted by accumulation of FM4-64 stain. **(D)** Micrograph collection of Marchantia cells stably co-expressing MpSEC6: Clover (green) or MpExo84:mScarlet (magenta). Scale bar is 10 µm. BF indicates Bright Field.

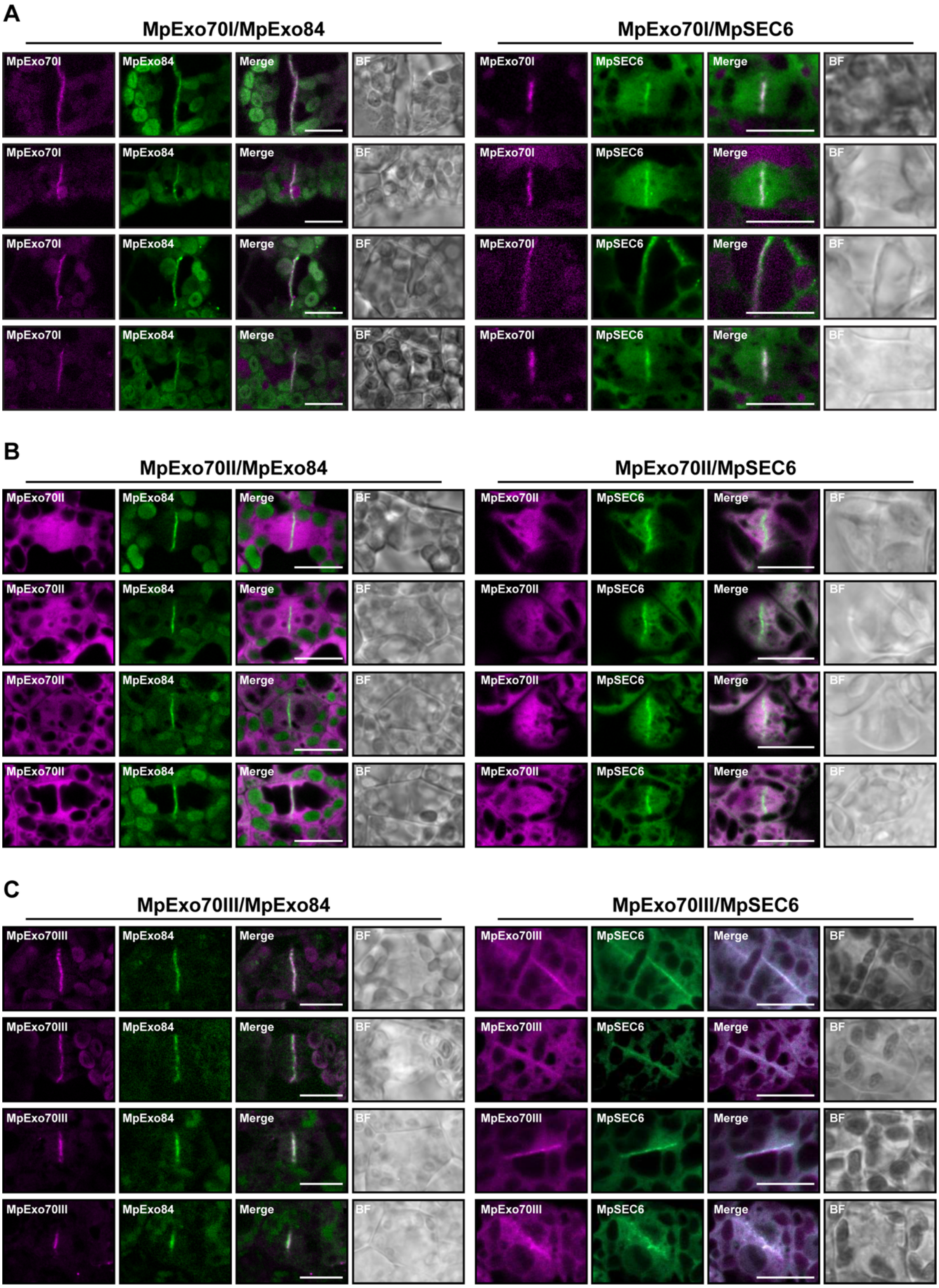
A
MpExo70I/MpExo84
MpExo70I
MpExo84
Merge
BF
MpExo70I/MpSEC6
MpSEC6
B
MpExo70II/MpExo84
MpExo70II
MpExo70II/MpSEC6
C
MpExo70III/MpExo84
MpExo70III
MpExo70III/MpSEC6

Fig. S5. Image gallery for MpExo70 co-localization with MpExo84 and MpSEC6 in Marchantia. Micrograph collection of Marchantia cells stably co-expressing MpExo84:Clover or MpSEC6:Clover (green) with **(A)** MpExo70I:mScarlet, **(B)** MpExo70II:mScarlet and **(C)** MpExo70III:mScarlet (magenta). The presence of the cell plate is depicted by accumulation of either MpExo84:Clover or MpSEC6:Clover. Scale bar is 10 µm. BF indicates Bright Field.

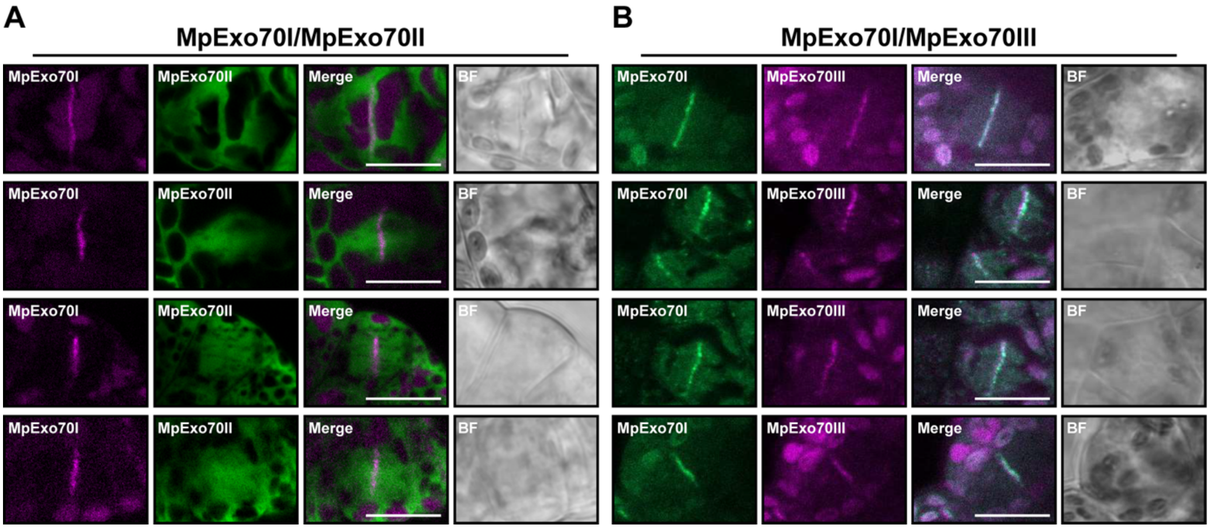

Fig. S6. Co-localization of MpExo70 proteins in Marchantia cells. Micrograph collection of Marchantia cells stably co-expressing **(A)** MpExo70I:mScarlet (magenta) and MpExo70II:Clover (green) or **(B)** MpExo70I:Clover (green) and MpExo70III:mScarlet (magenta). Scale bar is 10 µm. BF indicates Bright Field.

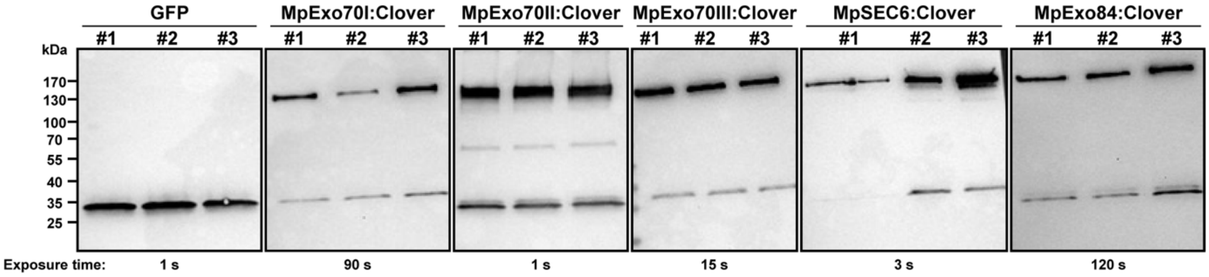

Fig. S7. Protein accumulation in IP-MS samples analyzed by Western blot. Prior to mass-spectrometry analysis, immunoprecipitates obtained with anti-GFP magnetic beads were probed for the presence of free GFP, MpExo70I:Clover, MpExo70II:Clover, MpExo70III:Clover, MpSEC6:Clover or MpExo84:Clover using anti-GFP antibody. Exposure time is indicated below each panel as the accumulation of different proteins varied consistently.

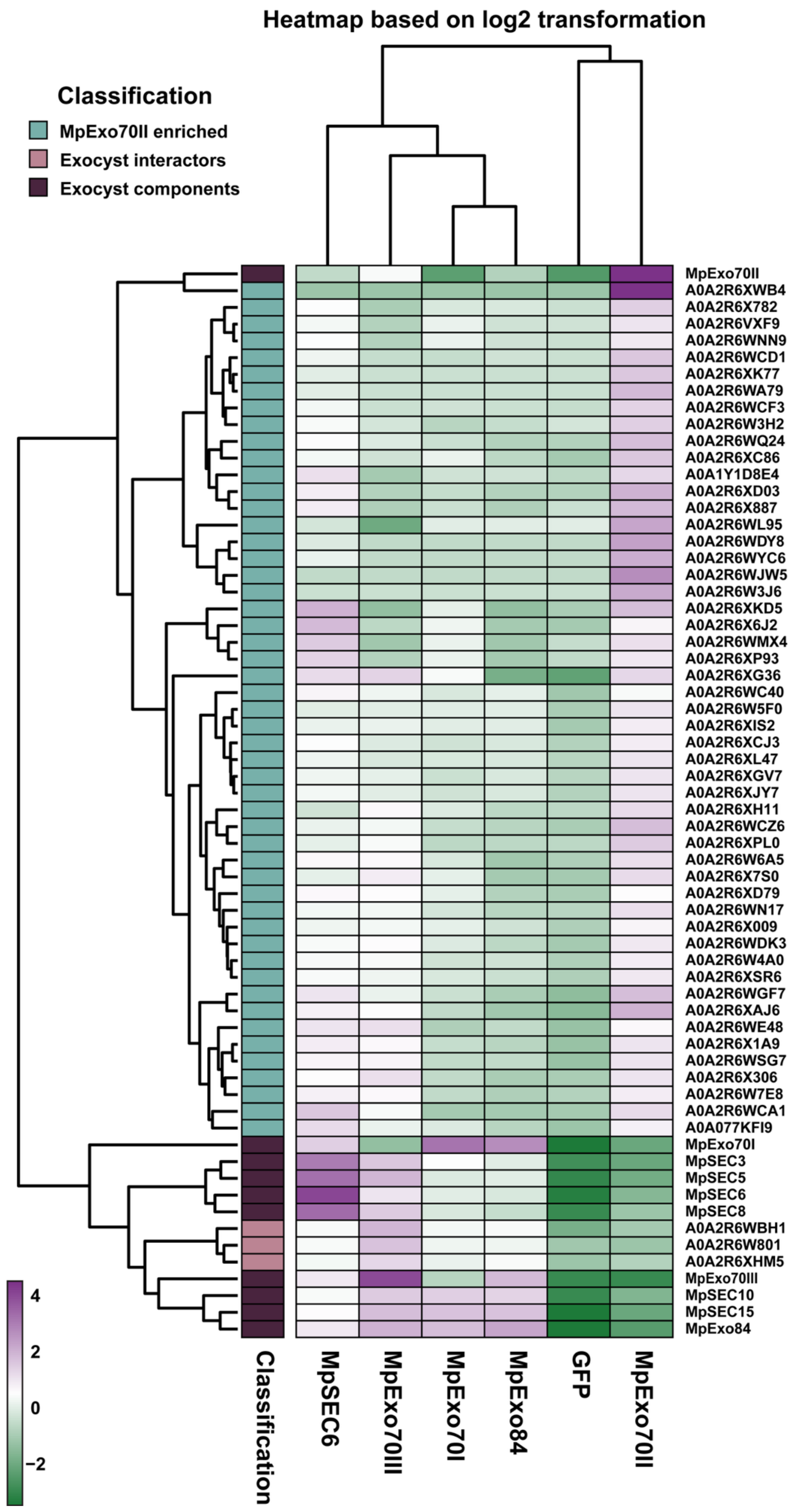
Heatmap based on log2 transformation
Classification
MpExo70II enriched
Exocyst interactors
Exocyst components
MpExo70II
A0A2R6XWB4
A0A2R6X782
A0A2R6VXF9
A0A2R6WNN9
A0A2R6WCD1
A0A2R6XK77
A0A2R6WA79
A0A2R6WCF3
A0A2R6W3H2
A0A2R6WQ24
A0A2R6XC86
A0A1Y1D8E4
A0A2R6XD03
A0A2R6X887
A0A2R6WL95
A0A2R6WDY8
A0A2R6WYC6
A0A2R6WJW5
A0A2R6W3J6
A0A2R6XKD5
A0A2R6X6J2
A0A2R6WMX4
A0A2R6XP93
A0A2R6XG36
A0A2R6WC40
A0A2R6W5F0
A0A2R6XIS2
A0A2R6XCJ3
A0A2R6XL47
A0A2R6XGV7
A0A2R6XJY7
A0A2R6XH11
A0A2R6WCZ6
A0A2R6XPL0
A0A2R6W6A5
A0A2R6X7S0
A0A2R6XD79
A0A2R6WN17
A0A2R6X009
A0A2R6WDK3
A0A2R6W4A0
A0A2R6XSR6
A0A2R6WGF7
A0A2R6XAJ6
A0A2R6WE48
A0A2R6X1A9
A0A2R6WSG7
A0A2R6X306
A0A2R6W7E8
A0A2R6WCA1
A0A077KFI9
MpExo70I
MpSEC3
MpSEC5
MpSEC6
MpSEC8
A0A2R6WBH1
A0A2R6W801
A0A2R6XHM5
MpExo70III
MpSEC10
MpSEC15
MpExo84
4
2
0
−2
Classification
MpSEC6
MpExo70III
MpExo70I
MpExo84
GFP
MpExo70II

Fig. S8. MpExo70 have different interactomes. (A) Protein abundance pattern represented by a heatmap (Log2(PSM+1) – meanPSM per protein, with values clipped between -3.5 and 4.5) for the exocyst components and the proteins identified as uniquely enriched in MpExo70II:Clover vs. GFP control dataset.

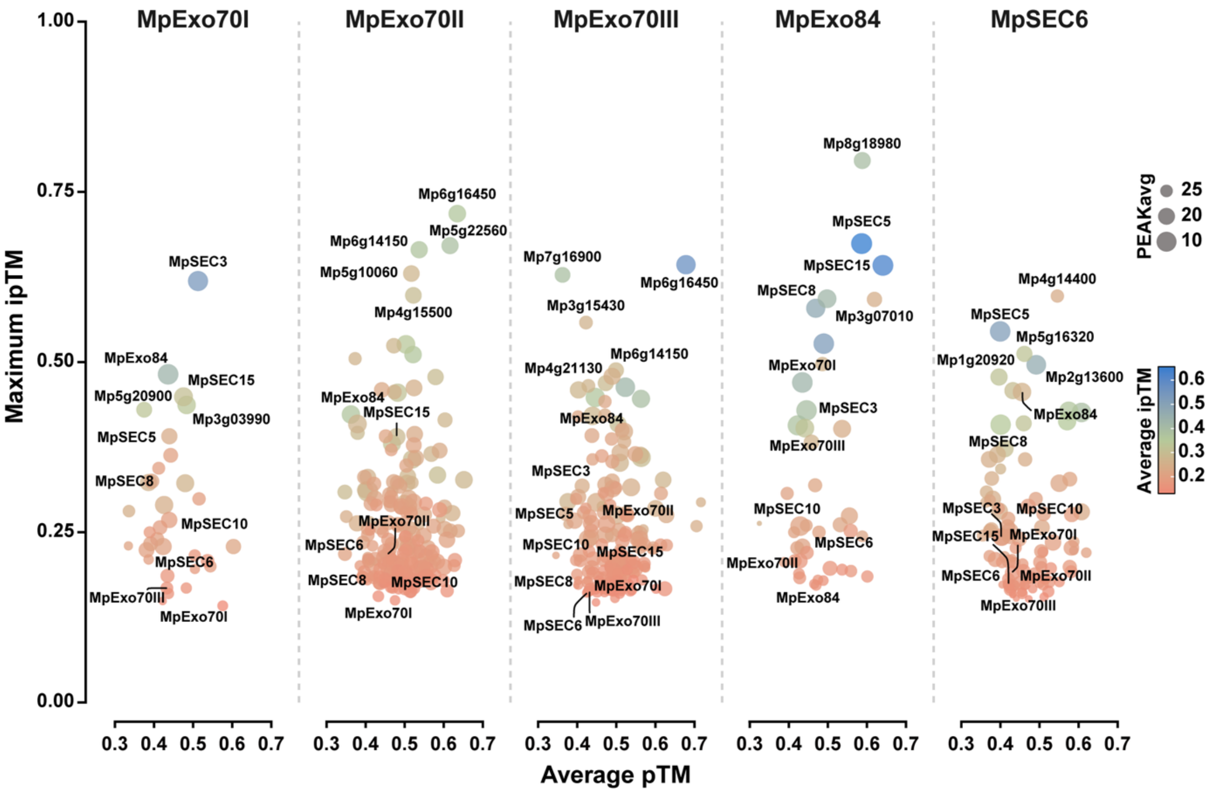

Fig. S9. Analysis of MpExo70 interactors by AlphaFold2. Scatterplot of AlphaFold-Multimer predicted ipTM versus pTM scores (ipTM: interface predicted Template Modeling score; pTM: predicted Template Modeling score) for MpExo70I, MpExo70II, MpExo70III, MpExo84 and MpSEC6 vs. their respective interactomes obtained by IP-MS. The maximum ipTM value from 5 independent predictions are use in the Y axis, while the average of pTM values from the 5 predictions is used in the X axis. The average ipTM is represented by the color of the dot. Dot size correlates to PEAK value average, where PEAK score represents average minimum predicted aligned error between protein chains excluding intra-molecular interactions. Top 5 candidates and exocyst components are indicated.

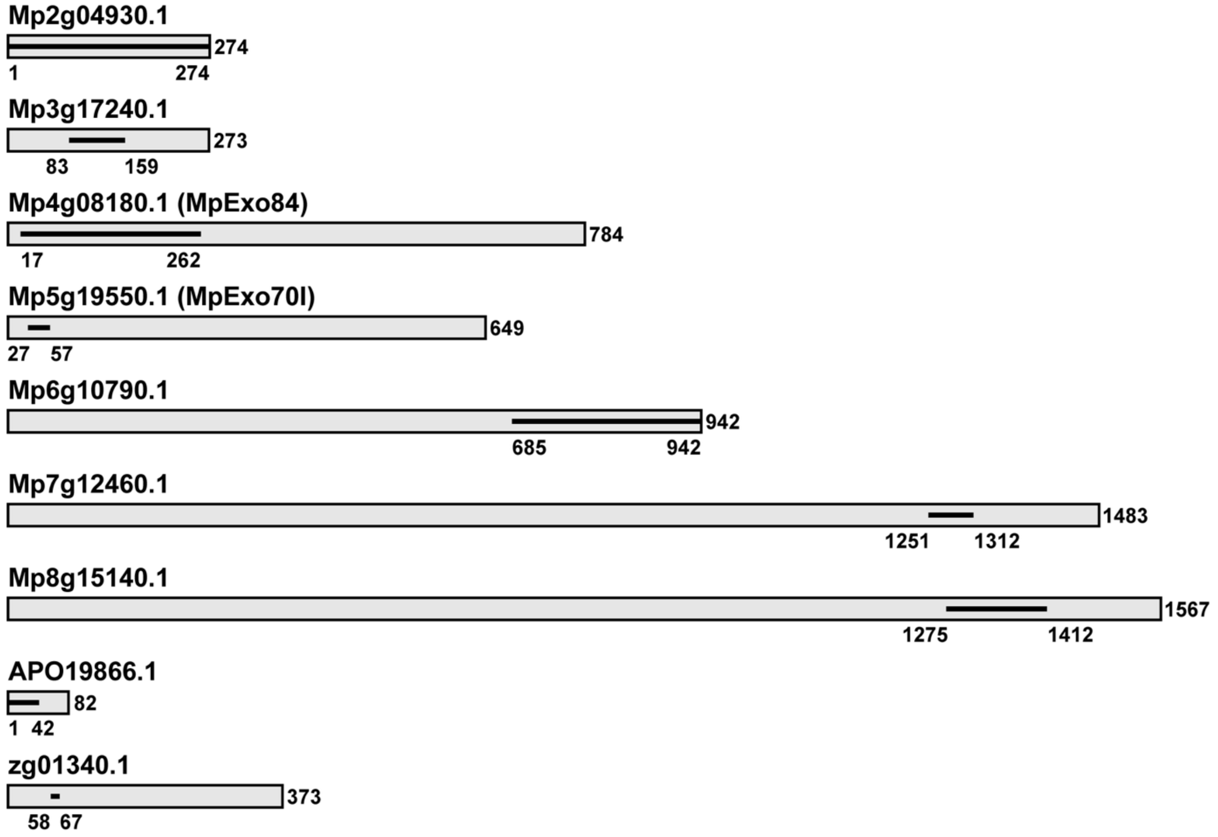

Fig. S10. Schematic representation of MpExo70I interactors obtained by genome-wide yeast-two-hybrid. For each candidate, full-length protein is represented by a grey box. The line inside the box represents the domain for which an interacting clone was found.

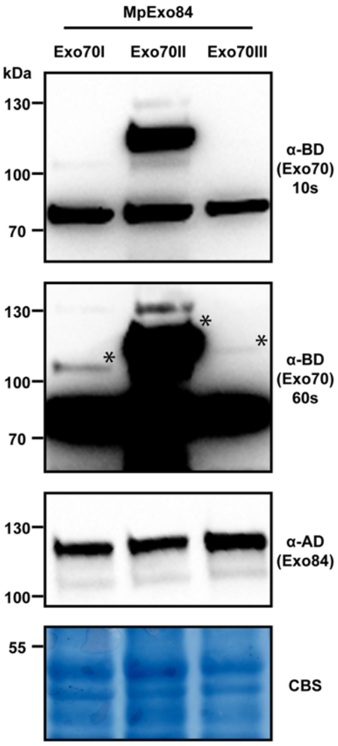

Fig. S11. Protein accumulation in Yeast-Two-Hybrid assay analyzed by Western blot. Yeast lysate was probed for the presence of MpExo70I, MpExo70II and MpExo70III using anti-GAL4 binding domain (BD); and MpExo84 using anti-GAL4 DNA activation domain (AD) antibodies. Total protein extracts were stained with Coomassie Blue Stain (CBS). Accumulation of MpExo70II was consistently higher and two panels with indicated exposure times are included. Asterisks indicate the bands corresponding to the proteins of interest.

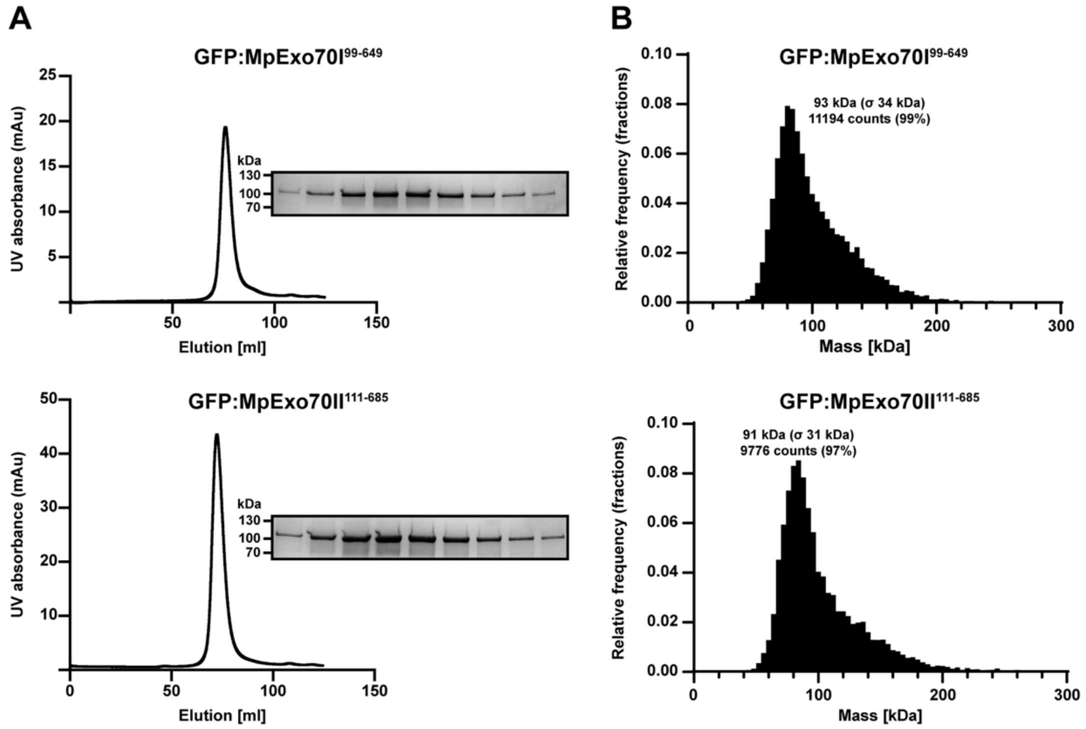

Fig. S12. Purification of GFP:Exo70 proteins from insect cells. (A) Elution trace of GFP:MpExo70I$^{99\text{-}649}$ and GFP:MpExo70II$^{111\text{-}685}$ after gel filtration and SDS-PAGE analysis of relevant elution peak. **(B)** Mass distribution of purified GFP:MpExo70I$^{99\text{-}649}$ (MW: 90 kDa) and GFP:MpExo70II$^{111\text{-}685}$ (MW: 93,6 kDa) estimated by mass photometry. For each peak, an estimated mass, standard deviation and number of counts is indicated.

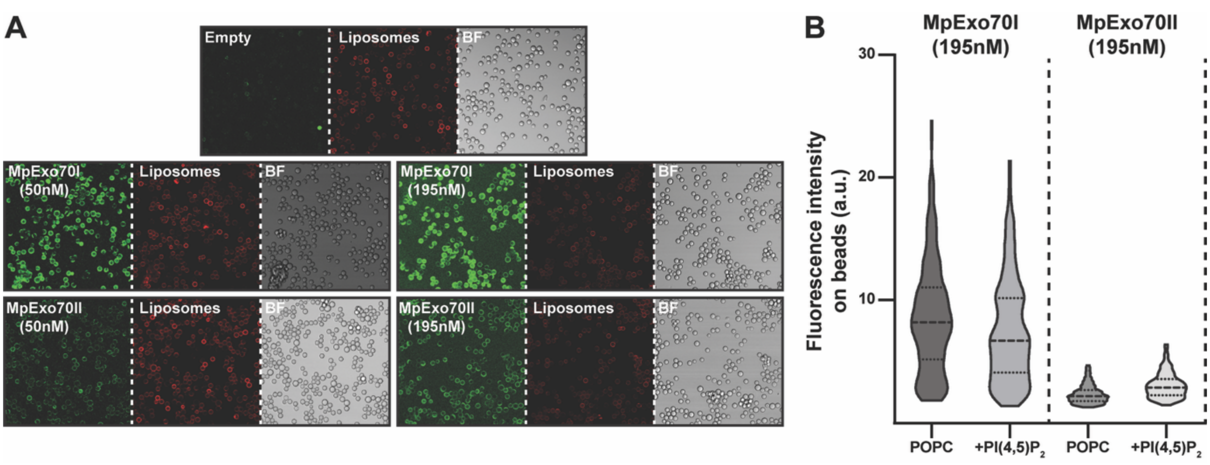

Fig. S13. MpExo70I and MpExo70II binds liposome with different affinity. (A) Representative confocal microscopy images of purified GFP:MpExo70I$^{99-649}$ and GFP:MpExo70II$^{111-685}$ (green) added to liposomes (red) at 50 nM and 195 nM concentrations. BF indicates Bright Field. **(B)** Violin plots with median and quartiles of GFP:MpExo70I$^{99-649}$ and GFP:MpExo70II$^{111-685}$ fluorescence intensity in liposomes assayed at 195 nM concentration.

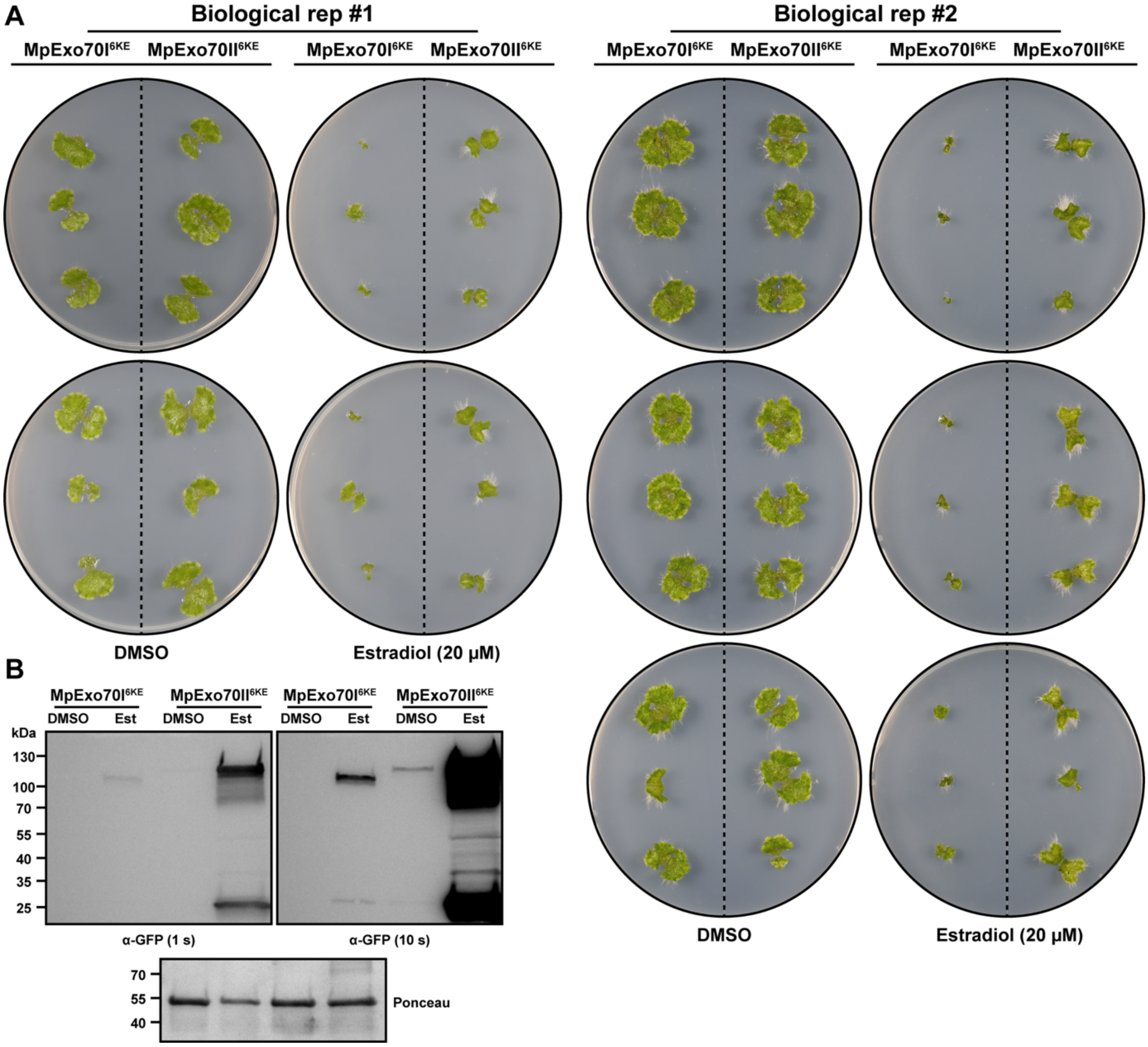

Fig. S14. Experimental replicates of inducible expression of MpExo70 KE mutants in Marchantia. (A) Macroscopic phenotypes of Marchantia transgenic lines XVE:MpExo70I:Clover, XVE:MpExo70I 6KE:Clover, XVE:MpExo70II:Clover and XVE:MpExo70II 6KE:Clover grown for 14 days on media containing B-estradiol (20 µM) or DMSO. **(B)** Accumulation of MpExo70I 6KE:Clover and MpExo70II 6KE:Clover after estradiol-mediated induction visualized by western blot.

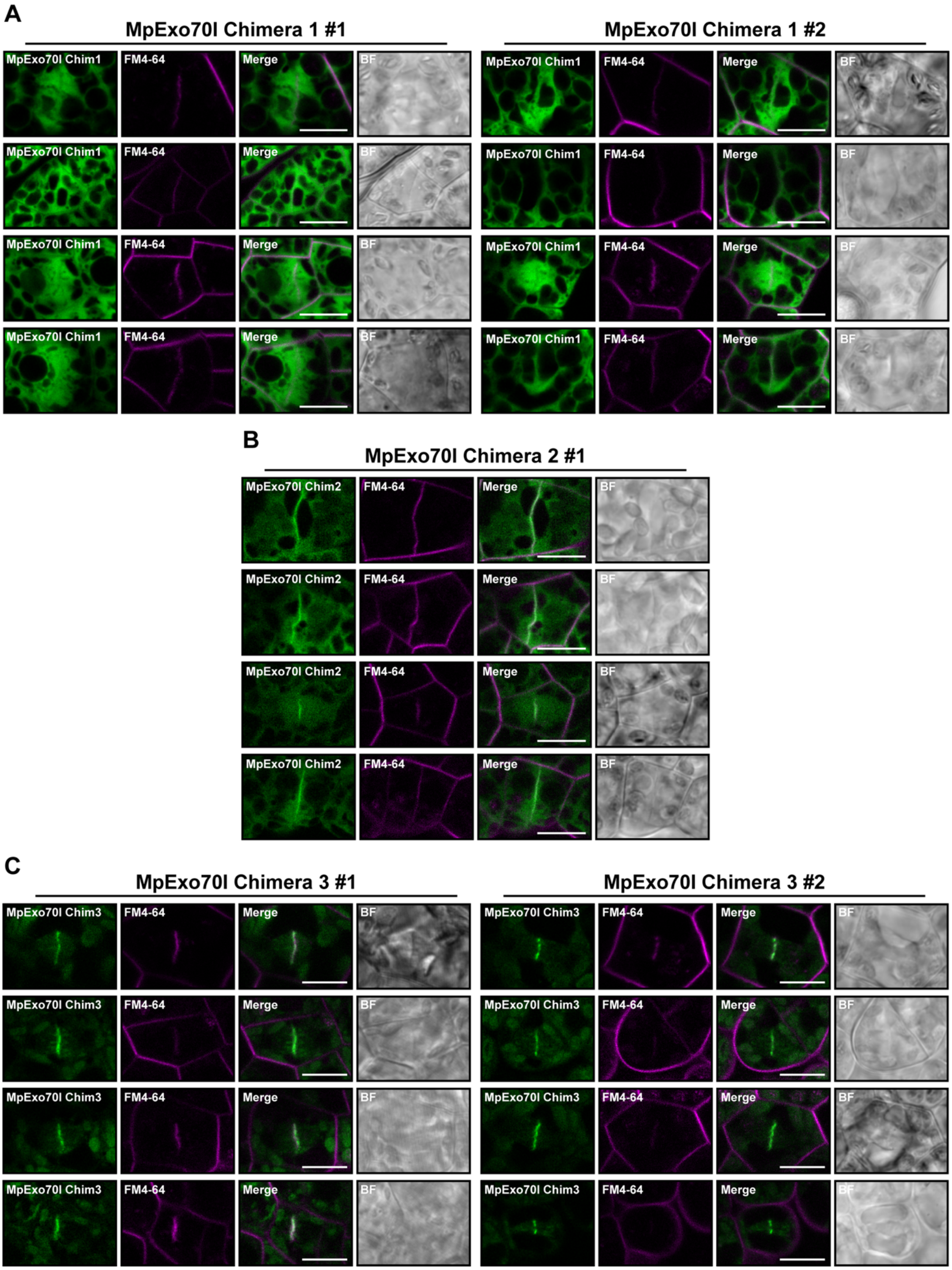
A
MpExo70I Chimera 1 #1
MpExo70I Chimera 1 #2
MpExo70I Chim1
FM4-64
Merge
BF
B
MpExo70I Chimera 2 #1
MpExo70I Chim2
C
MpExo70I Chimera 3 #1
MpExo70I Chimera 3 #2
MpExo70I Chim3

Fig. S15. Image gallery for localization of MpExo70I chimeras in Marchantia. Micrograph collection of Marchantia cells stably expressing **(A)** MpExo70I chimera 1:Clover, **(B)** MpExo70I chimera 2:Clover and **(C)** MpExo70I chimera 3:Clover stained with FM4-64 (magenta). Two independent lines were imaged except for MpExo70I chimera 2. The presence of the cell plate is depicted by accumulation of FM4-64 stain. Scale bar is 10 μm. BF indicates Bright Field.

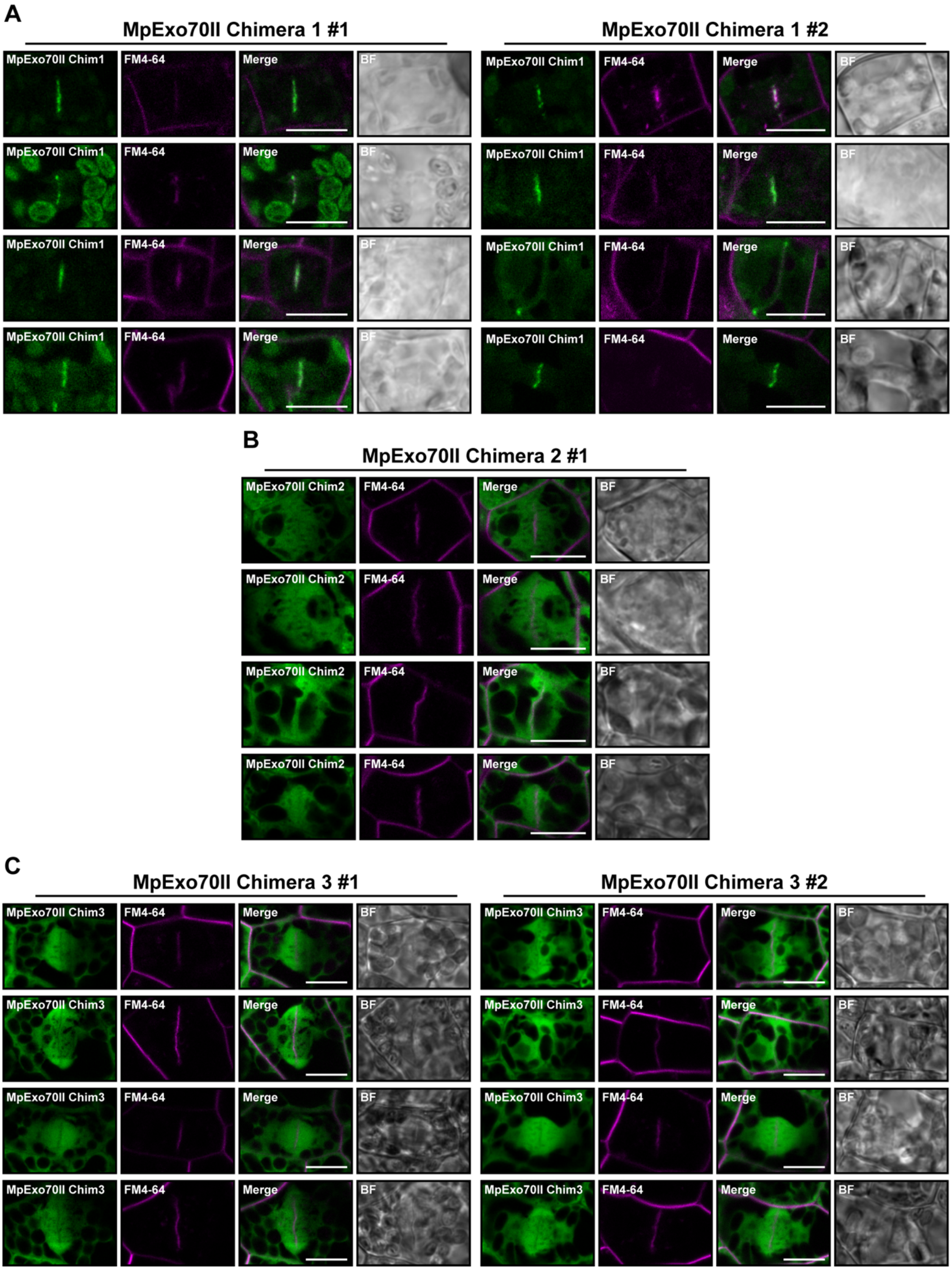
A
MpExo70II Chimera 1 #1
MpExo70II Chimera 1 #2
MpExo70II Chim1
FM4-64
Merge
BF
B
MpExo70II Chimera 2 #1
MpExo70II Chim2
FM4-64
Merge
BF
C
MpExo70II Chimera 3 #1
MpExo70II Chimera 3 #2
MpExo70II Chim3
FM4-64
Merge
BF

Fig. S16. Image gallery for localization of MpExo70II chimeras in Marchantia. Micrograph collection of Marchantia cells stably expressing **(A)** MpExo70II chimera 1:Clover, **(B)** MpExo70II chimera 2:Clover and **(C)** MpExo70II chimera 3:Clover stained with FM4-64 (magenta). Two independent lines were imaged except for MpExo70II chimera 2. The presence of the cell plate is depicted by accumulation of FM4-64 stain. Scale bar is 10 µm. BF indicates Bright Field.

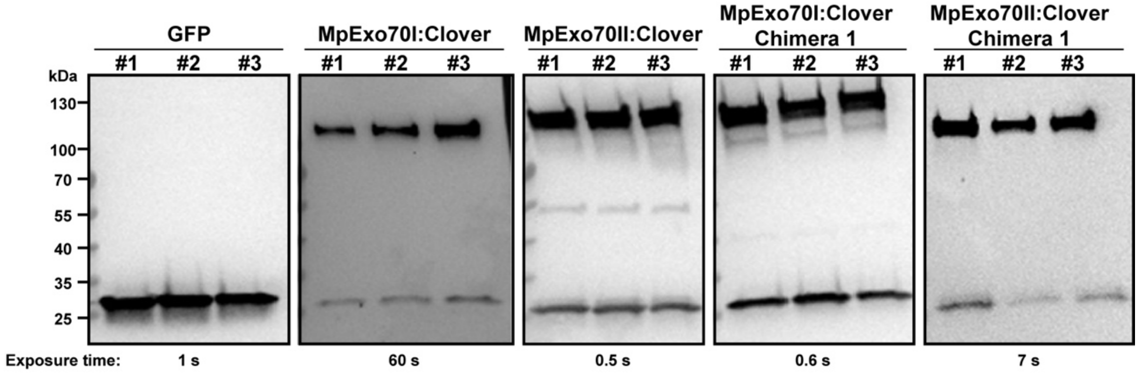

Fig. S17. Protein accumulation in IP-MS samples analyzed by Western blot. Prior to mass-spectrometry analysis, immunoprecipitates obtained with anti-GFP magnetic beads were probed for the presence of free GFP, MpExo70I:Clover, MpExo70II:Clover, MpExo70I chimera 1:Clover or MpExo70II chimera 1:Clover using anti-GFP antibody. Exposure time is indicated below each panel as the accumulation of different proteins varied consistently.

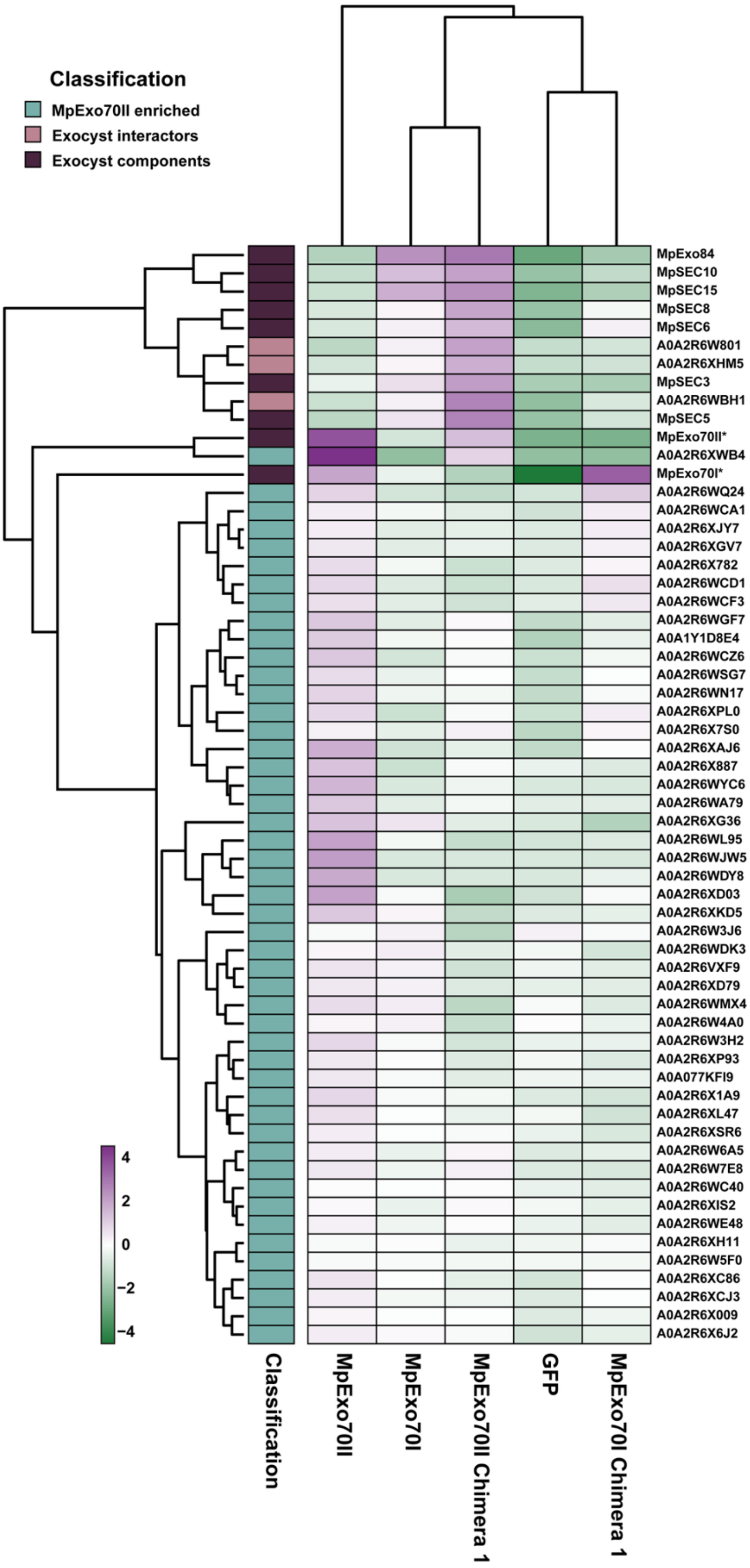

Classification
MpExo70II enriched
Exocyst interactors
Exocyst components
MpExo84
MpSEC10
MpSEC15
MpSEC8
MpSEC6
A0A2R6W801
A0A2R6XHM5
MpSEC3
A0A2R6WBH1
MpSEC5
MpExo70II*
A0A2R6XWB4
MpExo70I*
A0A2R6WQ24
A0A2R6WCA1
A0A2R6XJY7
A0A2R6XGV7
A0A2R6X782
A0A2R6WCD1
A0A2R6WCF3
A0A2R6WGF7
A0A1Y1D8E4
A0A2R6WCZ6
A0A2R6WSG7
A0A2R6WN17
A0A2R6XPL0
A0A2R6X7S0
A0A2R6XAJ6
A0A2R6X887
A0A2R6WYC6
A0A2R6WA79
A0A2R6XG36
A0A2R6WL95
A0A2R6WJW5
A0A2R6WDY8
A0A2R6XD03
A0A2R6XKD5
A0A2R6W3J6
A0A2R6WDK3
A0A2R6VXF9
A0A2R6XD79
A0A2R6WMX4
A0A2R6W4A0
A0A2R6W3H2
A0A2R6XP93
A0A077KFI9
A0A2R6X1A9
A0A2R6XL47
A0A2R6XSR6
A0A2R6W6A5
A0A2R6W7E8
A0A2R6WC40
A0A2R6XIS2
A0A2R6WE48
A0A2R6XH11
A0A2R6W5F0
A0A2R6XC86
A0A2R6XCJ3
A0A2R6X009
A0A2R6X6J2
4
2
0
−2
−4
Classification
MpExo70II
MpExo70I
MpExo70II Chimera 1
GFP
MpExo70I Chimera 1

Fig. S18. Replacement of N-terminal domain alter MpExo70s interactomes. Protein abundance pattern represented by a heatmap (Log2(PSM+1) – meanPSM per protein) for the exocyst components and the proteins identified as uniquely enriched in MpExo70II:Clover vs. GFP control dataset. Results represented are the mean from three independent replicates. Asterisk next to the name has been added as a cautionary mark indicating challenges in assigning the peptides to a chimera or a wild-type Exo70.

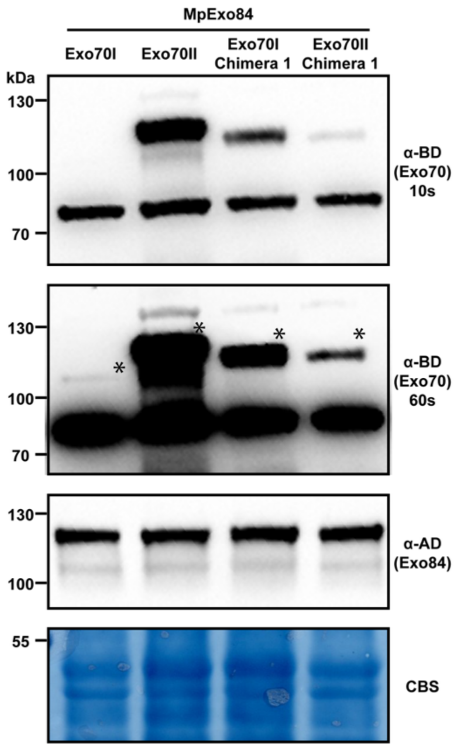

Fig. S19. Protein accumulation in Yeast-Two-Hybrid assay analyzed by Western blot. Yeast lysate was probed for the presence of MpExo70I, MpExo70II, MpExo70I chimera 1 and MpExo70II chimera 1 using anti-GAL4 binding domain (BD); and MpExo84 using anti-GAL4 DNA activation domain (AD) antibodies. Total protein extracts were stained with Coomassie Blue Stain (CBS). Accumulation of MpExo70II and MpExo70I chimera 1 were consistently higher and two panels with indicated exposure times are included. Asterisks indicate the bands corresponding to the proteins of interest.

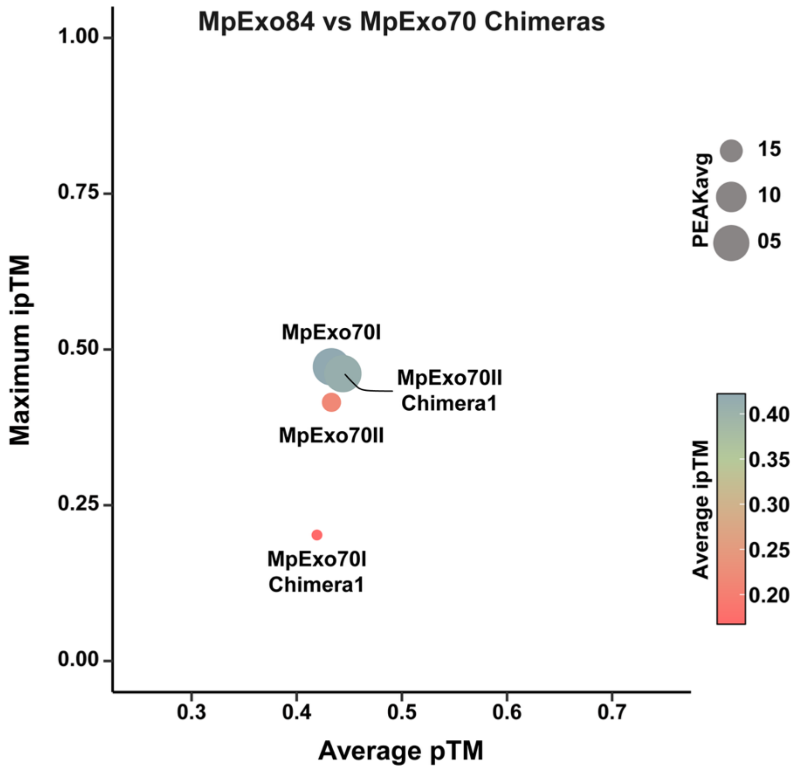

Fig. S20. Analysis of MpExo70 chimera interaction with MpExo84 by AlphaFold2. Scatterplot of AlphaFold-Multimer predicted ipTM versus pTM scores (ipTM: interface predicted Template Modeling score; pTM: predicted Template Modeling score) of the interaction between MpExo84 and MpExo70I, MpExo70II, MpExo70I chimera 1 or MpExo70II chimera 1. The maximum ipTM value from 5 independent predictions are use in the Y axis, while the average of pTM values from the 5 predictions is used in the X axis. The average ipTM is represented by the color of the dot. With the dot size correlating with the PEAK average. Dot size correlates to PEAK value average, where PEAK score represents average minimum predicted aligned error between protein chains excluding intra-molecular interactions.

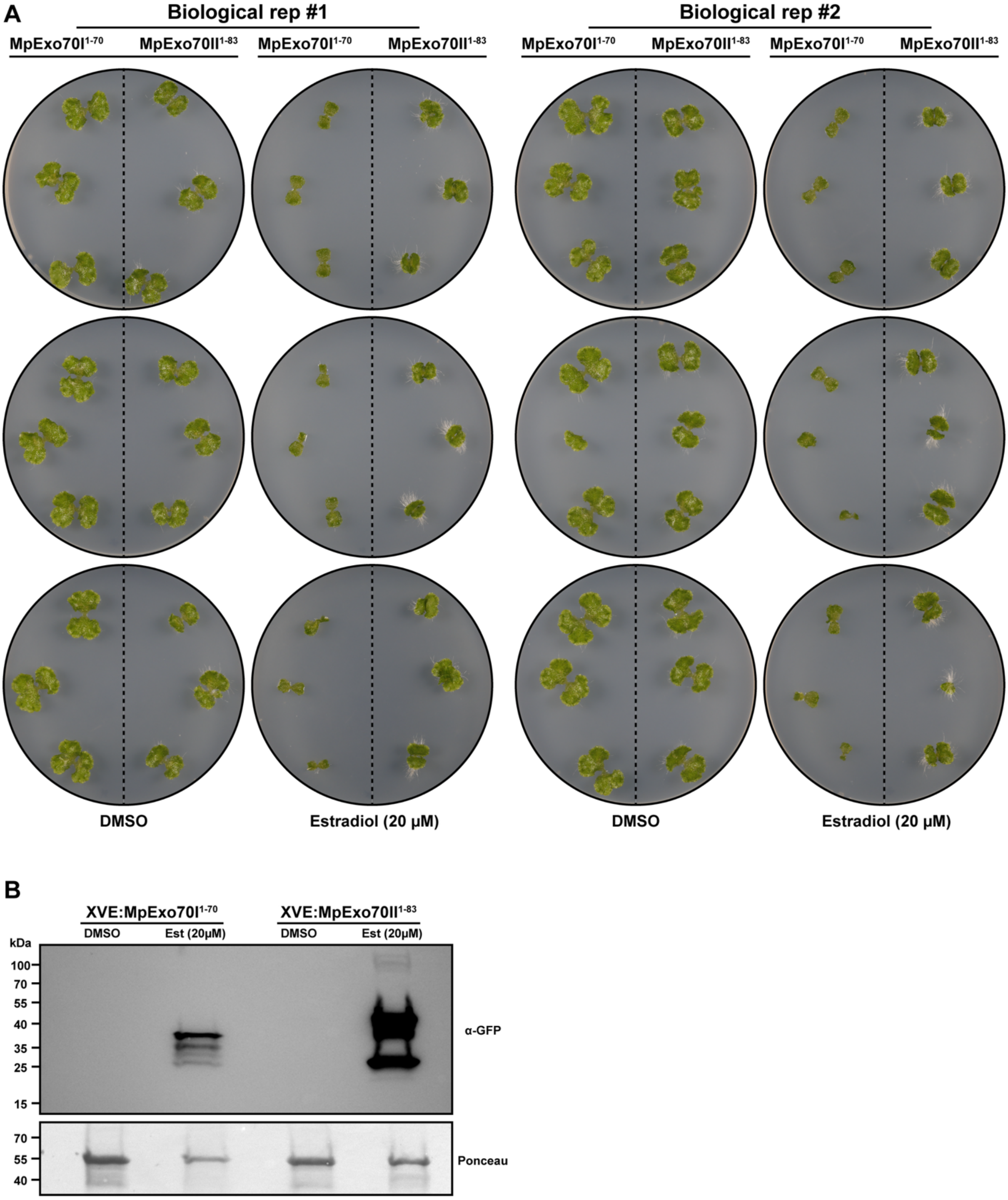

Fig. S21 Experimental replicates of inducible expression of MpExo70 N-terminal domain expression in Marchantia. (A) Macroscopic phenotypes of Marchantia transgenic lines XVE:MpExo70I$^{1-70}$:Clover and XVE:MpExo70II$^{1-83}$ grown for 14 days on media containing B-estradiol (20 µM) or DMSO. **(B)** Accumulation of MpExo70I$^{1-70}$:Clover and MpExo70II$^{1-83}$:Clover after estradiol-mediated induction visualized by western blot.

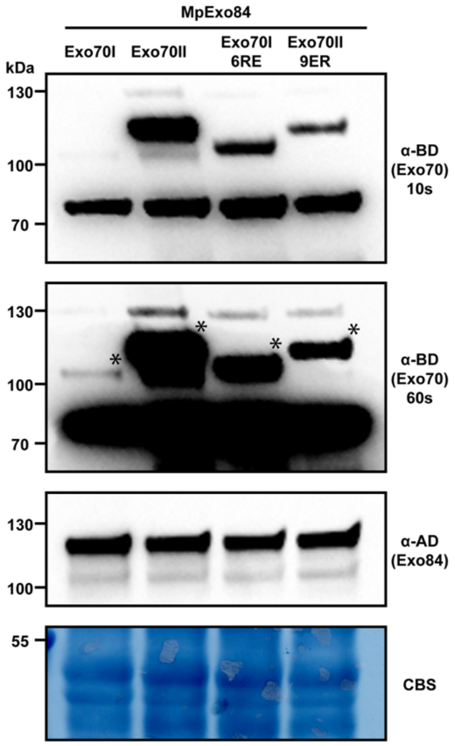

Fig. S22. Protein accumulation in Yeast-Two-Hybrid assay analyzed by Western blot. Yeast lysate was probed for the presence of MpExo70I, MpExo70II, MpExo70I 6RE and MpExo70II 9ER using anti-GAL4 binding domain (BD); and MpExo84 using anti-GAL4 DNA activation domain (AD) antibodies. Total protein extracts were stained with Coomassie Blue Stain (CBS). Accumulation of MpExo70I was consistently lower and two panels with indicated exposure times are included. Asterisks indicate the bands corresponding to the proteins of interest.

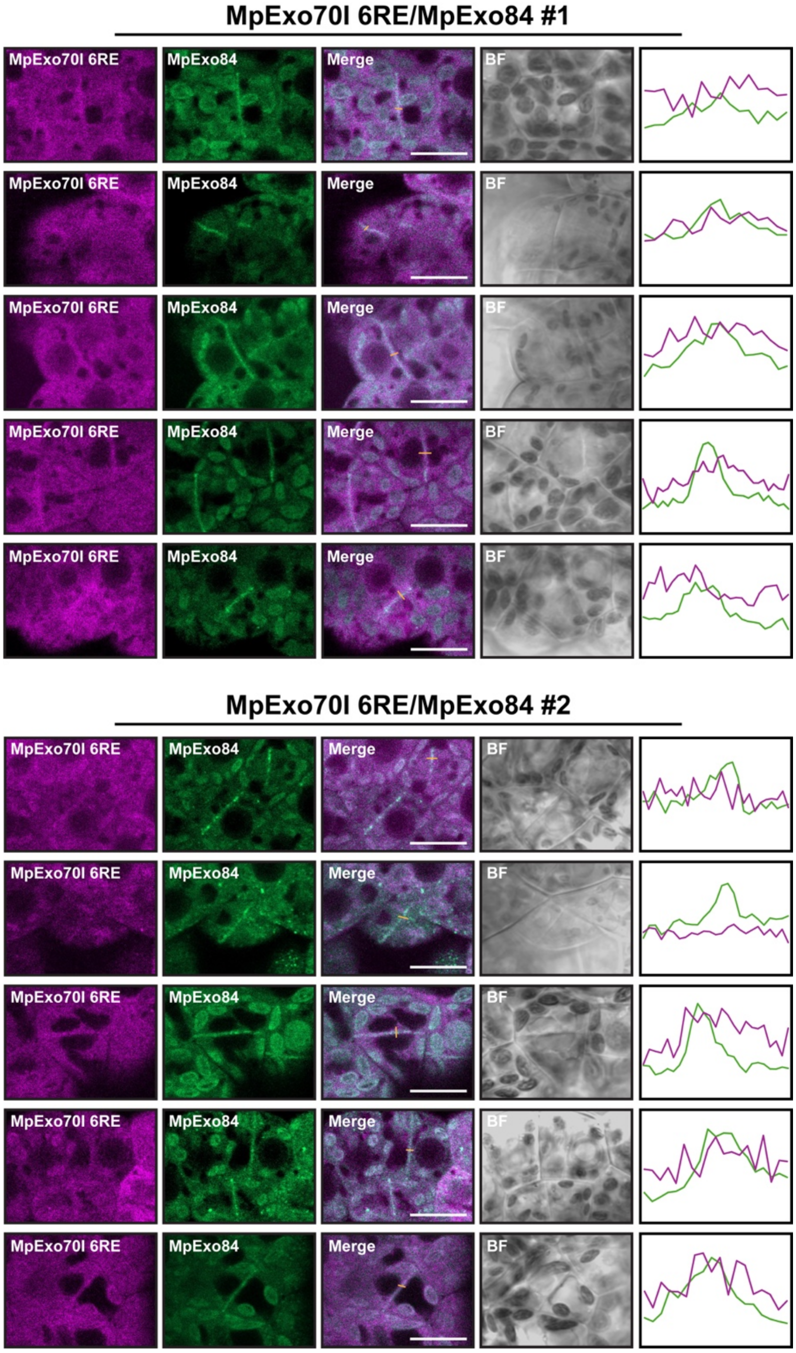

Fig. S23. Image gallery for MpExo70I 6RE co-localization with MpExo84 in Marchantia. Micrograph collection of two independent Marchantia cell lines stably co-expressing MpExo84:Clover (green) with MpExo70I 6RE:mScarlet (magenta). The presence of the cell plate is depicted by accumulation of MpExo84:Clover. Right panels represent the fluorescence intensity profiles of Clover (green) and mScarlet (magenta) measured along the distance of the selected orange lines in merge channel. The X axis represents distance in microns while the Y axis represents intensity (a.u). Scale bar is 10 µm. BF indicates Bright Field.

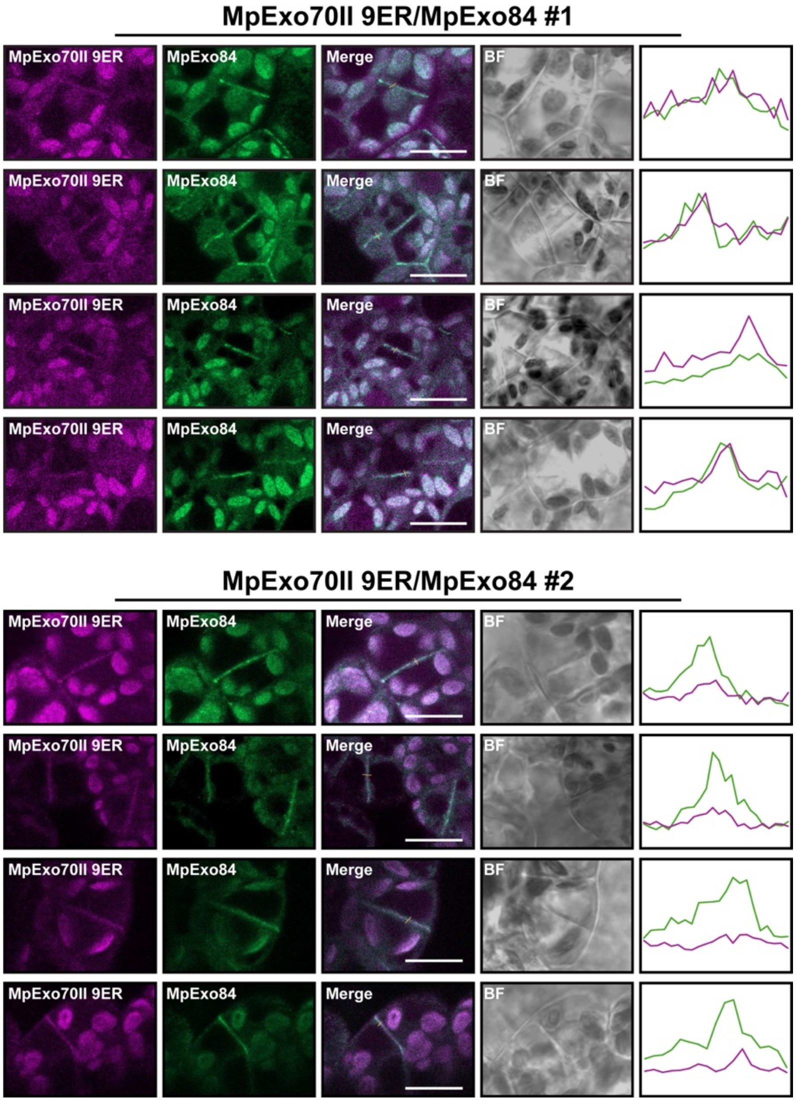

Fig. S24. Image gallery for MpExo70II 9ER co-localization with MpExo84 in Marchantia. Micrograph collection of two independent Marchantia cell lines stably co-expressing MpExo84:Clover (green) with MpExo70I 9ER:mScarlet (magenta). The presence of the cell plate is depicted by accumulation of MpExo84:Clover. Right panels represent the fluorescence intensity profiles of Clover (green) and mScarlet (magenta) measured along the distance of the selected orange lines in merge channel. The X axis represents distance in microns while the Y axis represents intensity (a.u). Scale bar is 10 μm. BF indicates Bright Field.

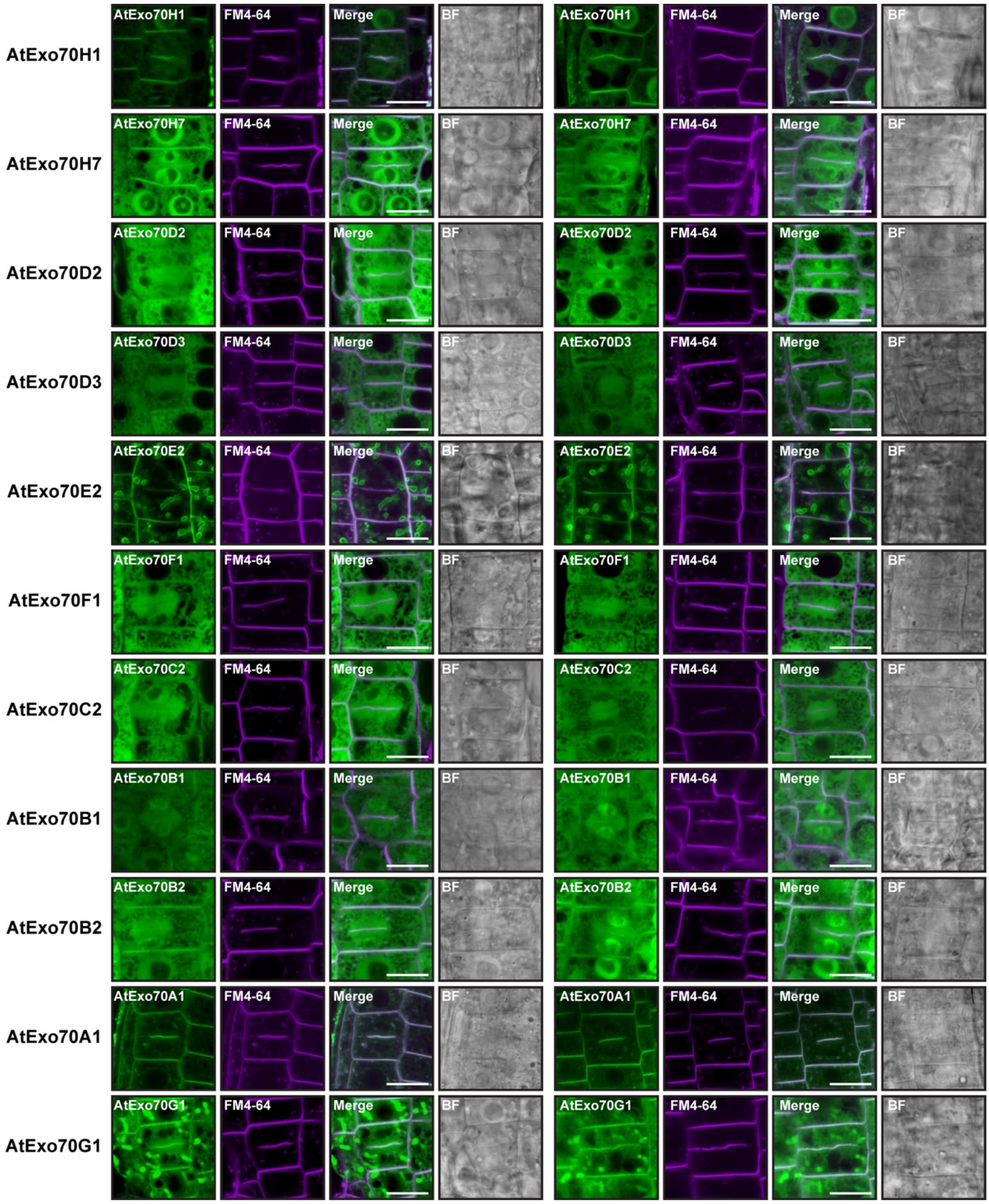

Fig. S25. Image gallery for AtExo70 localization in Arabidopsis root cells. Micrograph collection of Arabidopsis root cells stably expressing C-terminally GFP tagged AtExo70 proteins (green) stained with FM4-64 (magenta). The presence of the cell plate is depicted by accumulation of FM4-64 stain. Scale bar is 10 µm. BF indicates Bright Field.
