# Supplementary material for: Electrostatic changes enabled the diversification of an exocyst subunit via protein complex escape
Source: Nat Plants. 2025 Oct 31;11(11):2350–67. doi: 10.1038/s41477-025-02135-1 (PMC12626893; doi:10.1038/s41477-025-02135-1)
Supplement: Supplementary file 2 — Reporting Summary [file 41477_2025_2135_MOESM2_ESM.pdf]

nature portfolio

Corresponding author(s): Juan Carlos De la Concepcion
Nicholas Irwin
Yasin Dagdas

Last updated by author(s): Sep 3, 2025

Reporting Summary

Nature Portfolio wishes to improve the reproducibility of the work that we publish. This form provides structure for consistency and transparency in reporting. For further information on Nature Portfolio policies, see our Editorial Policies and the Editorial Policy Checklist.

Statistics

For all statistical analyses, confirm that the following items are present in the figure legend, table legend, main text, or Methods section.

n/a	Confirmed	
☐	☒	The exact sample size (*n*) for each experimental group/condition, given as a discrete number and unit of measurement
☐	☒	A statement on whether measurements were taken from distinct samples or whether the same sample was measured repeatedly
☐	☒	The statistical test(s) used AND whether they are one- or two-sided<br>*Only common tests should be described solely by name; describe more complex techniques in the Methods section.*
☒	☐	A description of all covariates tested
☒	☐	A description of any assumptions or corrections, such as tests of normality and adjustment for multiple comparisons
☐	☒	A full description of the statistical parameters including central tendency (e.g. means) or other basic estimates (e.g. regression coefficient) AND variation (e.g. standard deviation) or associated estimates of uncertainty (e.g. confidence intervals)
☐	☒	For null hypothesis testing, the test statistic (e.g. *F*, *t*, *r*) with confidence intervals, effect sizes, degrees of freedom and *P* value noted<br>*Give P values as exact values whenever suitable.*
☒	☐	For Bayesian analysis, information on the choice of priors and Markov chain Monte Carlo settings
☒	☐	For hierarchical and complex designs, identification of the appropriate level for tests and full reporting of outcomes
☐	☒	Estimates of effect sizes (e.g. Cohen's *d*, Pearson's *r*), indicating how they were calculated

Our web collection on statistics for biologists contains articles on many of the points above.

Software and code

Policy information about availability of computer code

Data collection	All instruments and software used for data collection are detailed in the Methods section
Data analysis	description of all commercial and readily available software for data analysis are detailed in the Methods section

For manuscripts utilizing custom algorithms or software that are central to the research but not yet described in published literature, software must be made available to editors and reviewers. We strongly encourage code deposition in a community repository (e.g. GitHub). See the Nature Portfolio guidelines for submitting code & software for further information.

Data

Policy information about availability of data

All manuscripts must include a data availability statement. This statement should provide the following information, where applicable:

- Accession codes, unique identifiers, or web links for publicly available datasets
- A description of any restrictions on data availability
- For clinical datasets or third party data, please ensure that the statement adheres to our policy

Source data and raw images used to generate the main and supplementary figures are deposited to Zenodo (https://zenodo.org/records/13374123).The mass spectrometry proteomics data have been deposited to the ProteomeXchange Consortium via the PRIDE partner repository with the dataset identifier PXD051853.

Research involving human participants, their data, or biological material

Policy information about studies with human participants or human data. See also policy information about sex, gender (identity/presentation), and sexual orientation and race, ethnicity and racism.

Reporting on sex and gender	n/a
Reporting on race, ethnicity, or other socially relevant groupings	n/a
Population characteristics	n/a
Recruitment	n/a
Ethics oversight	n/a

Note that full information on the approval of the study protocol must also be provided in the manuscript.

Field-specific reporting

Please select the one below that is the best fit for your research. If you are not sure, read the appropriate sections before making your selection.

☒ Life sciences ☐ Behavioural & social sciences ☐ Ecological, evolutionary & environmental sciences

For a reference copy of the document with all sections, see nature.com/documents/nr-reporting-summary-flat.pdf

Life sciences study design

All studies must disclose on these points even when the disclosure is negative.

Sample size	No sample size calculation was performed. Sample size was based on previous literature and experimental logistic. The data is consistent within and between independent experiments.
Data exclusions	No data points were excluded
Replication	All assays were repeated in triplicates with comparable results. All replication attempts were successfull
Randomization	No experimental group was used in this study.
Blinding	No blinding has been used. Blinding was not relevant for our study as the order or position of samples would not affect the results

Reporting for specific materials, systems and methods

We require information from authors about some types of materials, experimental systems and methods used in many studies. Here, indicate whether each material, system or method listed is relevant to your study. If you are not sure if a list item applies to your research, read the appropriate section before selecting a response.

Materials & experimental systems

n/a	Involved in the study	
☐	☒	Antibodies
☐	☒	Eukaryotic cell lines
☒	☐	Palaeontology and archaeology
☒	☐	Animals and other organisms
☒	☐	Clinical data
☒	☐	Dual use research of concern
☐	☒	Plants

Methods

n/a	Involved in the study	
☒	☐	ChIP-seq
☒	☐	Flow cytometry
☒	☐	MRI-based neuroimaging

Antibodies

Antibodies used	GFP was detected using the anti-GFP antibody (Mouse monoclonal, 11814460001; Roche) diluted 1:3000 (v/v)<br>RFP was detected using anti-RFP (AB_2631395, Chromotek) diluted 1:3000 (v/v).<br>SIGMA Anti-GAL4 DNA-BD antibody produced in rabbit, Cat no. G3042. Used diluted 1:3000.

SIGMA Anti-GAL4 Activation domain antibody produced in rabbit, Cat no. G9293. Used diluted 1:3000.
SUMO-tag Monoclonal Antibody (4G11E9, Thermo Fischer Scientific) at 1:1000 dilution
Goat Anti-Mouse IgG (H + L)-HRP antibody (1706516, Bio-Rad) 1:10000 in blocking buffer.
Rabbit polyclonal antibody was detected with a goat anti-rabbit IgG HRP-linked antibody (65-6120; Invitrogen) diluted 1:5000.

Validation: All the antibodies used in this study are commercial and were validated by manufacturers.

Eukaryotic cell lines

Policy information about cell lines and Sex and Gender in Research

Cell line source(s): Hi5 insect cell lines

Authentication: Cell were commercially purchased

Mycoplasma contamination: Performed by the provider

Commonly misidentified lines (See ICLAC register): *Name any commonly misidentified cell lines used in the study and provide a rationale for their use.*

Dual use research of concern

Policy information about dual use research of concern

Hazards

Could the accidental, deliberate or reckless misuse of agents or technologies generated in the work, or the application of information presented in the manuscript, pose a threat to:

No	Yes	
☒	☐	Public health
☒	☐	National security
☒	☐	Crops and/or livestock
☒	☐	Ecosystems
☒	☐	Any other significant area

Experiments of concern

Does the work involve any of these experiments of concern:

No	Yes	
☒	☐	Demonstrate how to render a vaccine ineffective
☒	☐	Confer resistance to therapeutically useful antibiotics or antiviral agents
☒	☐	Enhance the virulence of a pathogen or render a nonpathogen virulent
☒	☐	Increase transmissibility of a pathogen
☒	☐	Alter the host range of a pathogen
☒	☐	Enable evasion of diagnostic/detection modalities
☒	☐	Enable the weaponization of a biological agent or toxin
☒	☐	Any other potentially harmful combination of experiments and agents

Plants

Seed stocks: No plant specimen was collected from the field. All seed stocks are described in Supplementary Table S1.

Novel plant genotypes: Novel plant genotypes were generated by Agrobacterium mediated plant transformation by floral dipping in Arabidopsis thaliana and gemmae transformation in the case of Marchantia polymorpha. As described in the Materials and methods. All novel genotypes are described in Supplementary Table S1.

Authentication: Authentication of generated genotypes were carried out by western blot for detection of the protein produced by the inserted transgene
