# Supplementary figures and images for: Electrostatic changes enabled the diversification of an exocyst subunit via protein complex escape
Source: Nat Plants. 2025 Oct 31;11(11):2350–67. doi: 10.1038/s41477-025-02135-1 (PMC12626893; doi:10.1038/s41477-025-02135-1)

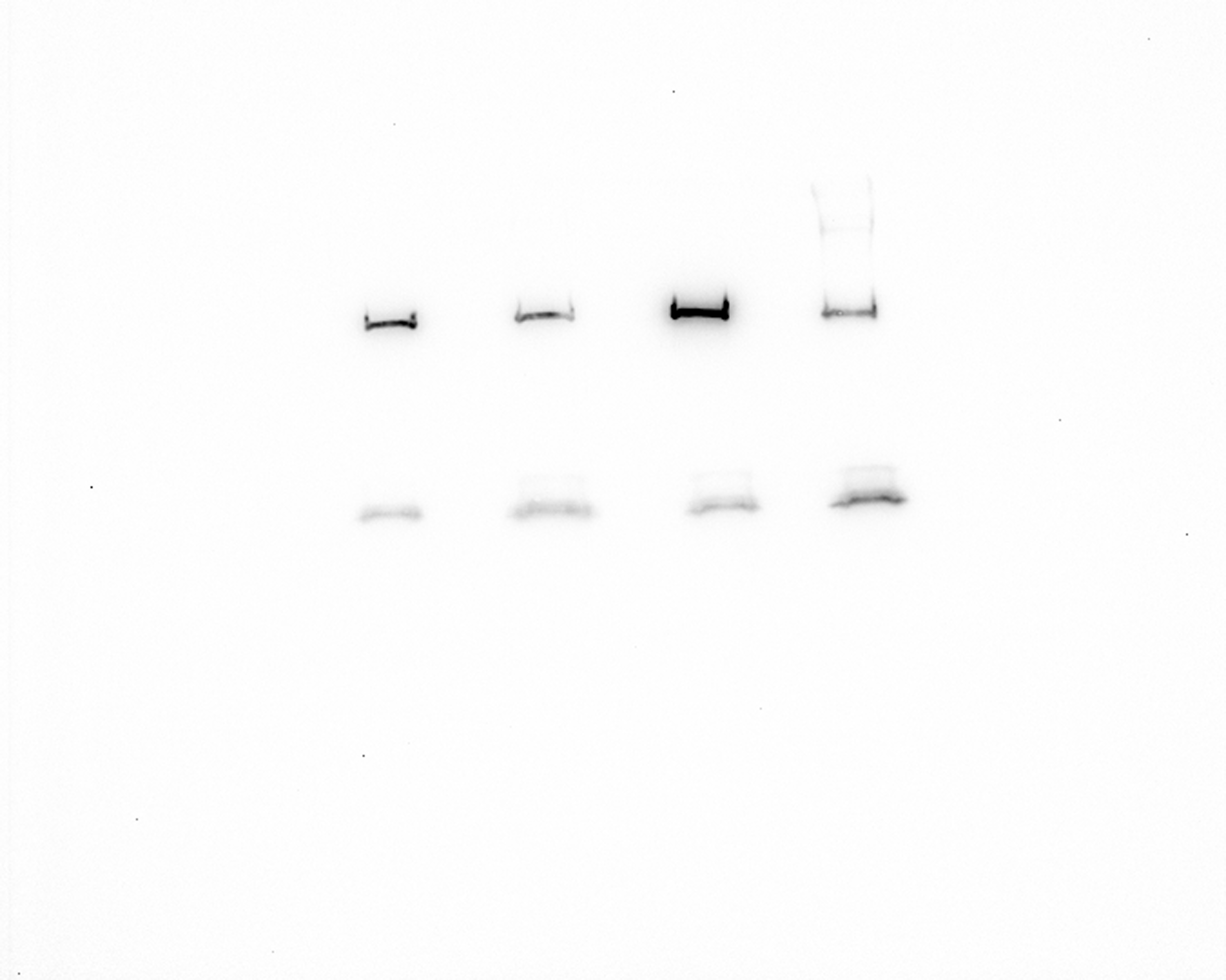

Supplement: Supplementary file 8 — Unprocessed western blots. [file 41477_2025_2135_MOESM8_ESM.zip › Source blots/Figure 4c/Figure 4c GFP IP.tif]

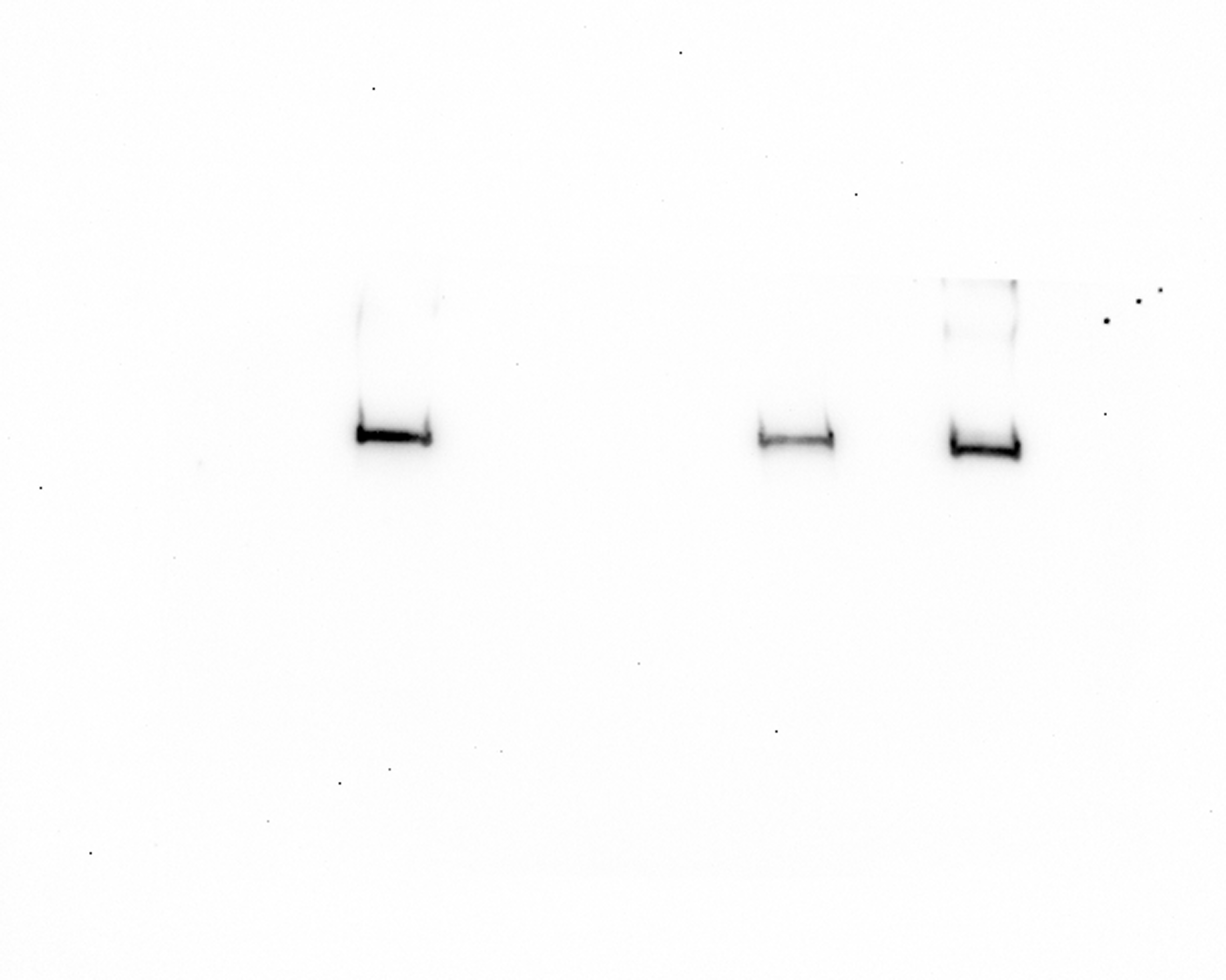

Supplement: Supplementary file 8 — Unprocessed western blots. [file 41477_2025_2135_MOESM8_ESM.zip › Source blots/Figure 4c/Figure 4c RFP IP.tif]

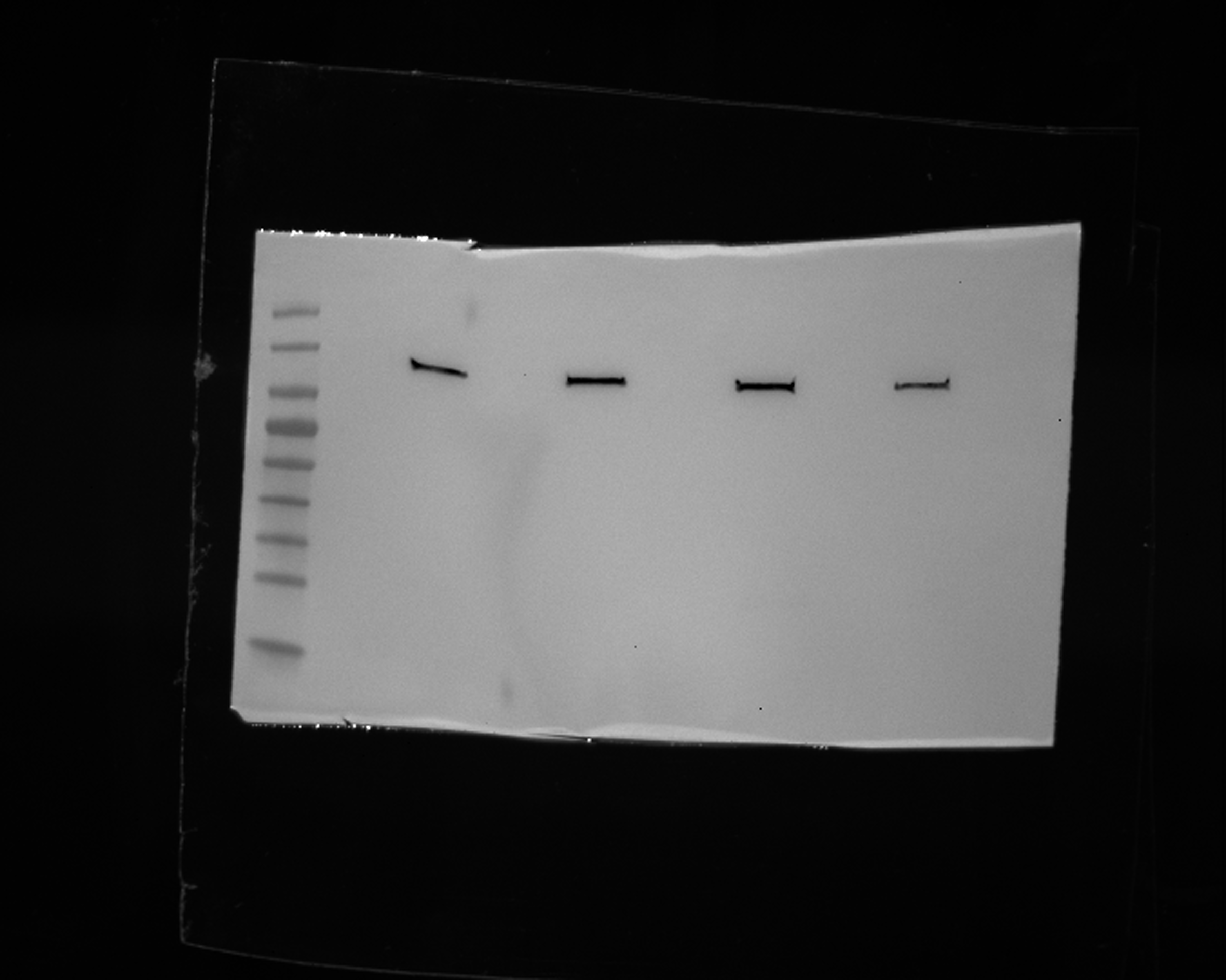

Supplement: Supplementary file 8 — Unprocessed western blots. [file 41477_2025_2135_MOESM8_ESM.zip › Source blots/Figure 4c/Figure 4c GFP input (Overlay).tif]

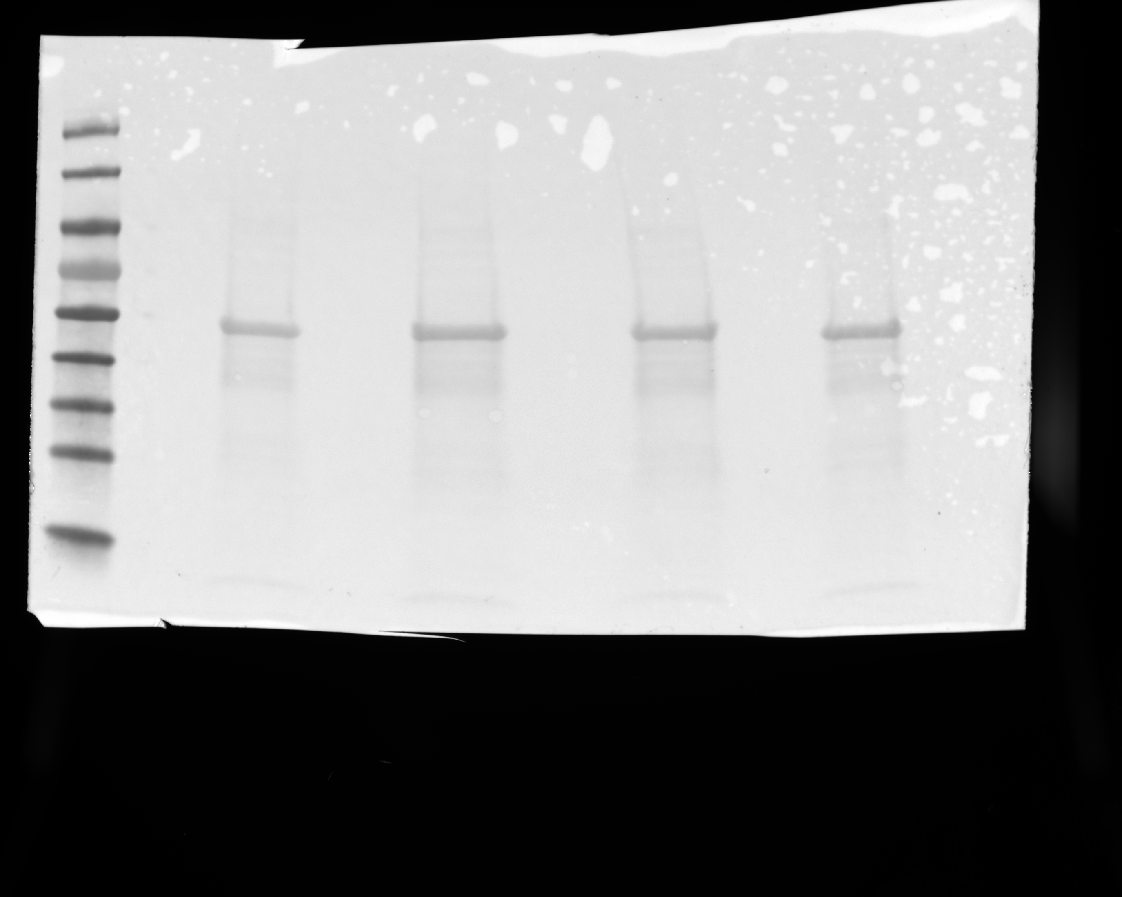

Supplement: Supplementary file 8 — Unprocessed western blots. [file 41477_2025_2135_MOESM8_ESM.zip › Source blots/Figure 4c/Figure 4c Ponceau.tif]

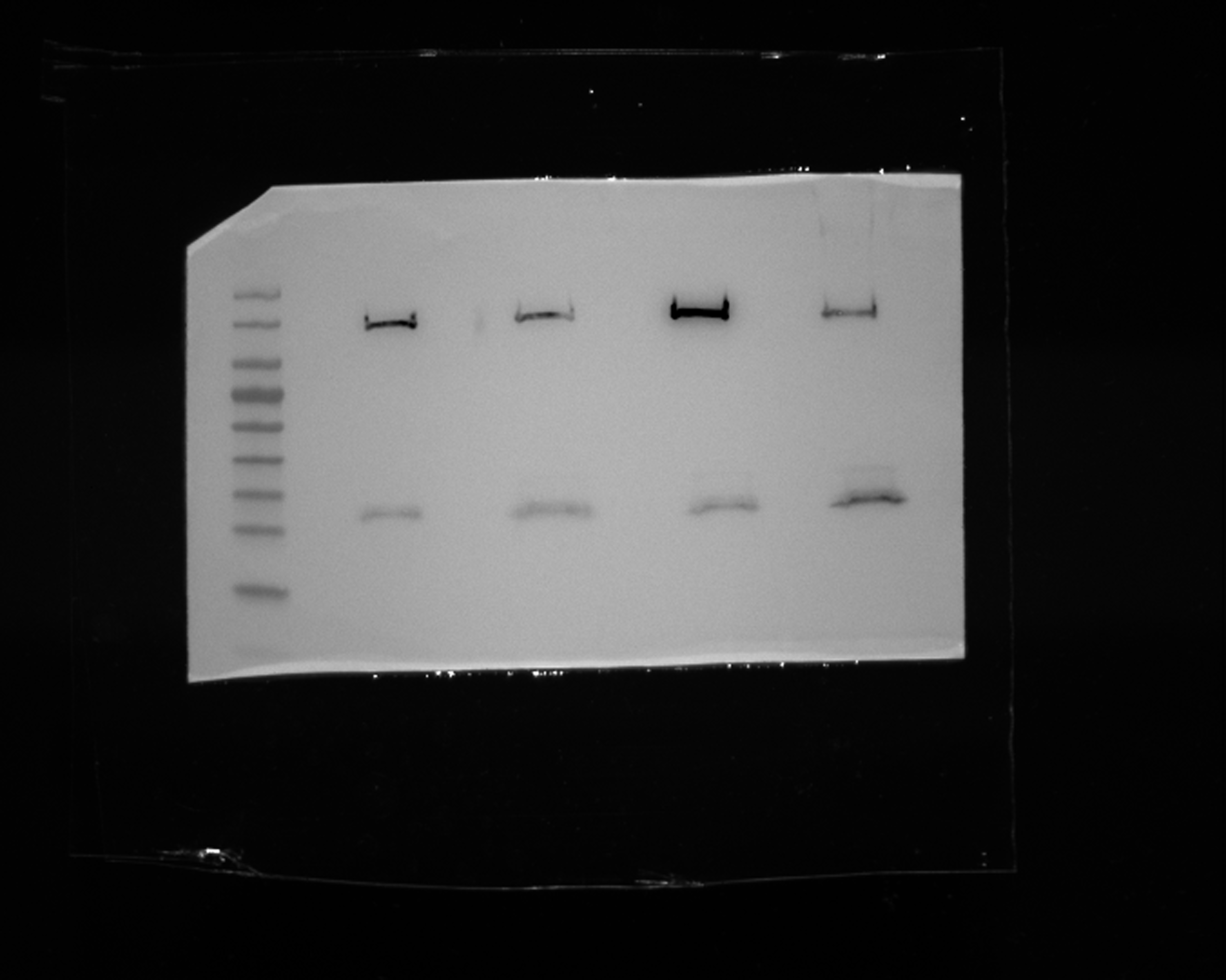

Supplement: Supplementary file 8 — Unprocessed western blots. [file 41477_2025_2135_MOESM8_ESM.zip › Source blots/Figure 4c/Figure 4c GFP IP (Overlay).tif]

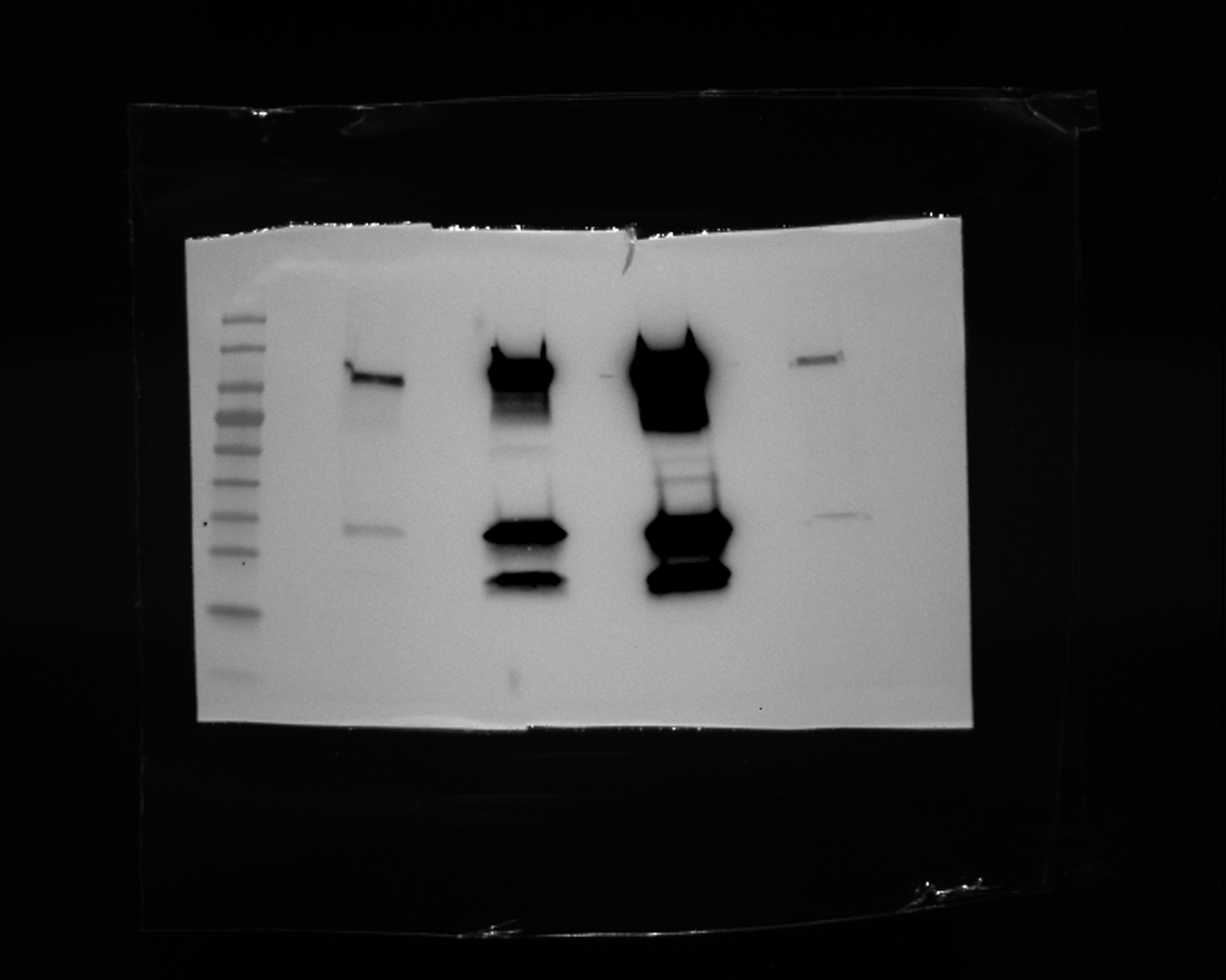

Supplement: Supplementary file 8 — Unprocessed western blots. [file 41477_2025_2135_MOESM8_ESM.zip › Source blots/Figure 4c/Figure 4c RFP input (Overlay).tif]

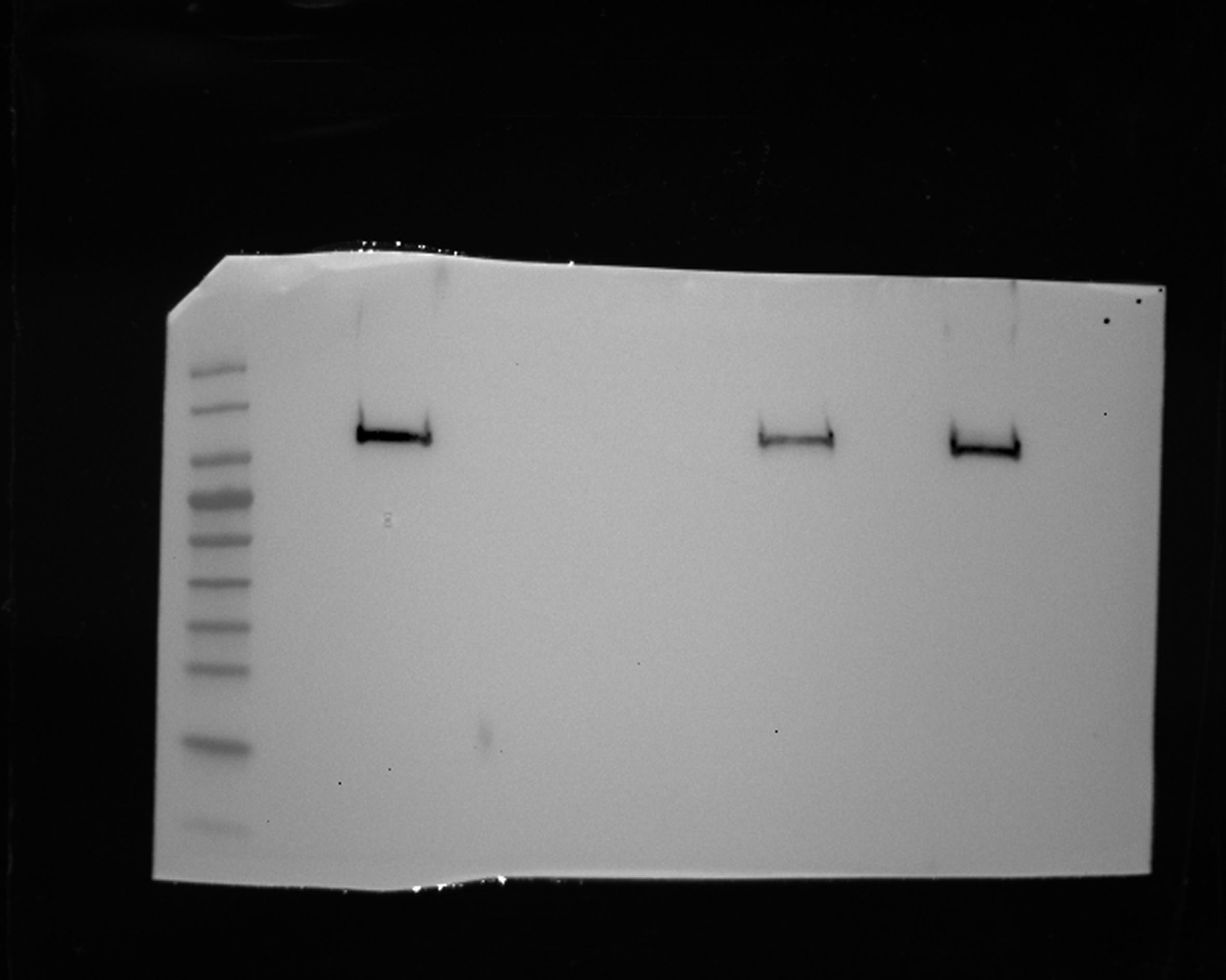

Supplement: Supplementary file 8 — Unprocessed western blots. [file 41477_2025_2135_MOESM8_ESM.zip › Source blots/Figure 4c/Figure 4c RFP IP (Overlay).tif]

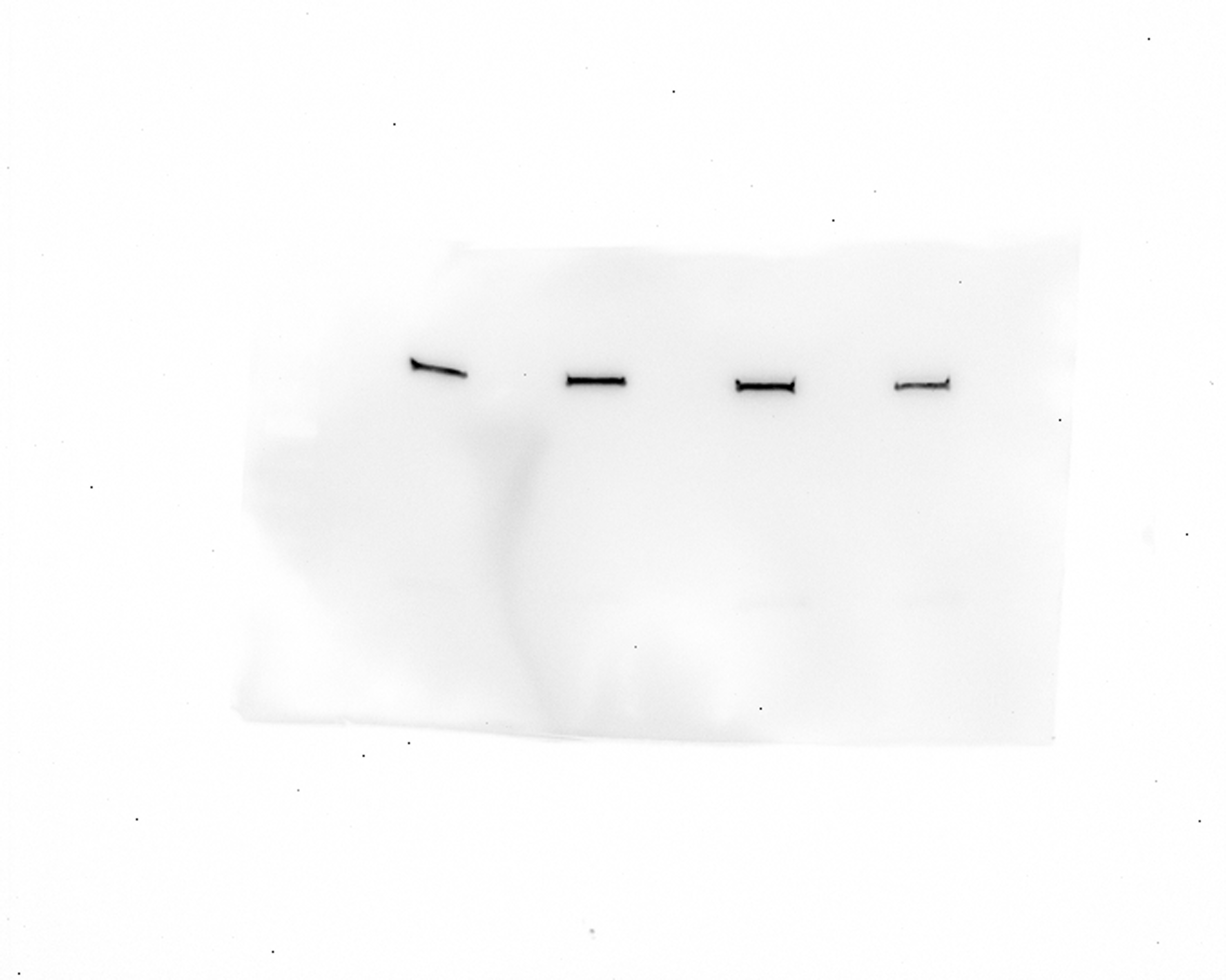

Supplement: Supplementary file 8 — Unprocessed western blots. [file 41477_2025_2135_MOESM8_ESM.zip › Source blots/Figure 4c/Figure 4c GFP input.tif]

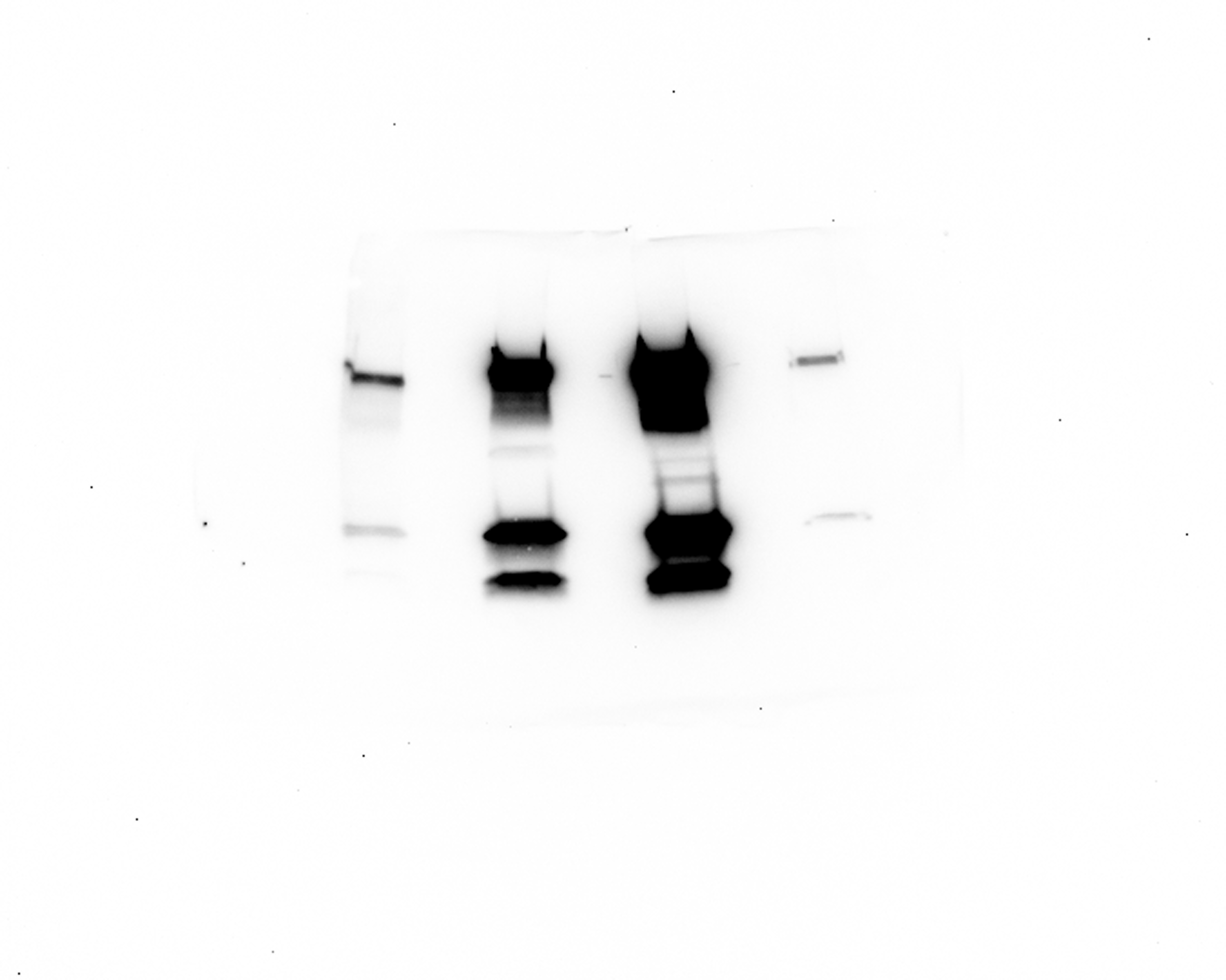

Supplement: Supplementary file 8 — Unprocessed western blots. [file 41477_2025_2135_MOESM8_ESM.zip › Source blots/Figure 4c/Figure 4c RFP input.tif]

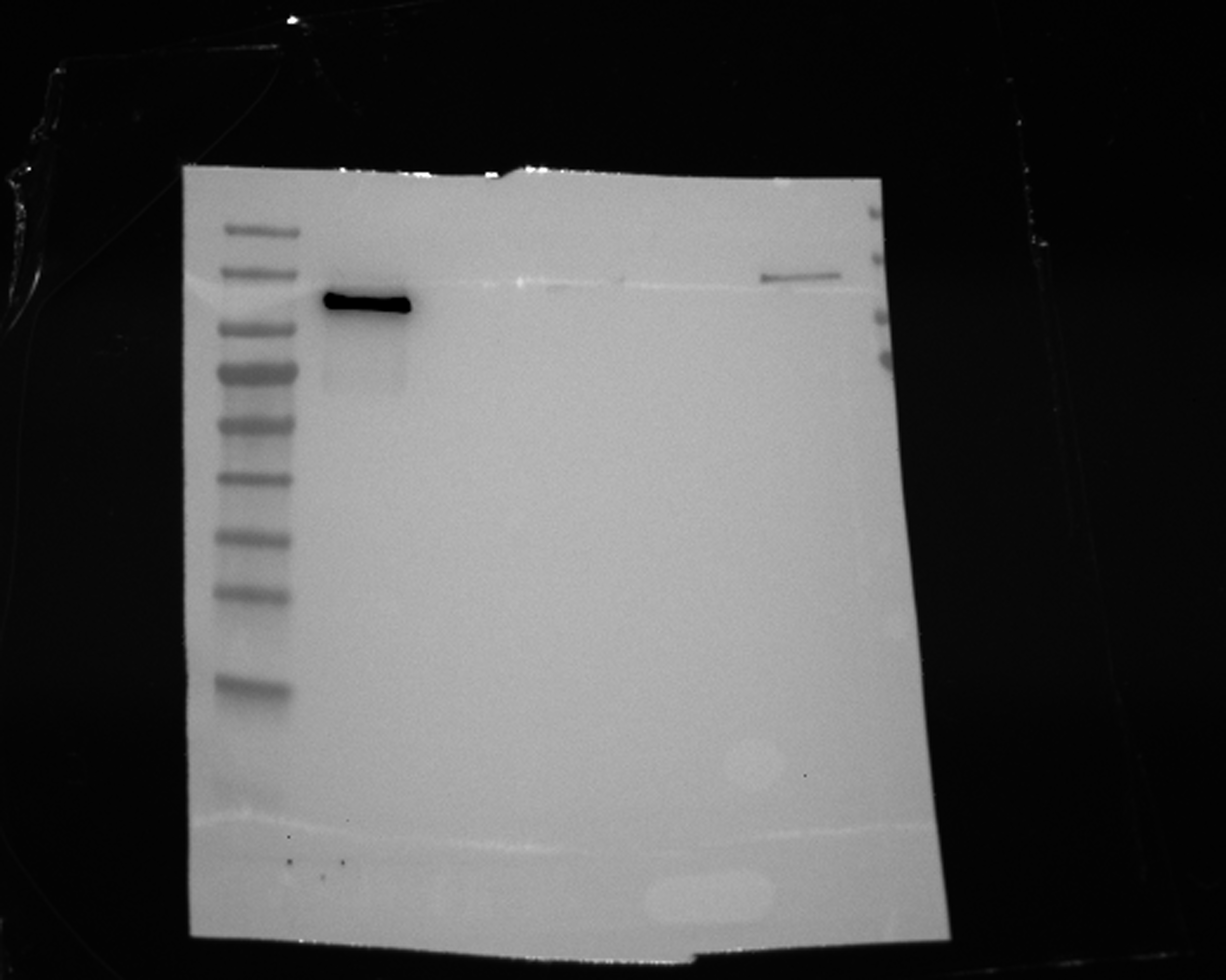

Supplement: Supplementary file 8 — Unprocessed western blots. [file 41477_2025_2135_MOESM8_ESM.zip › Source blots/Figure 1e/Figure 1e RFP IP(Overlay).tif]

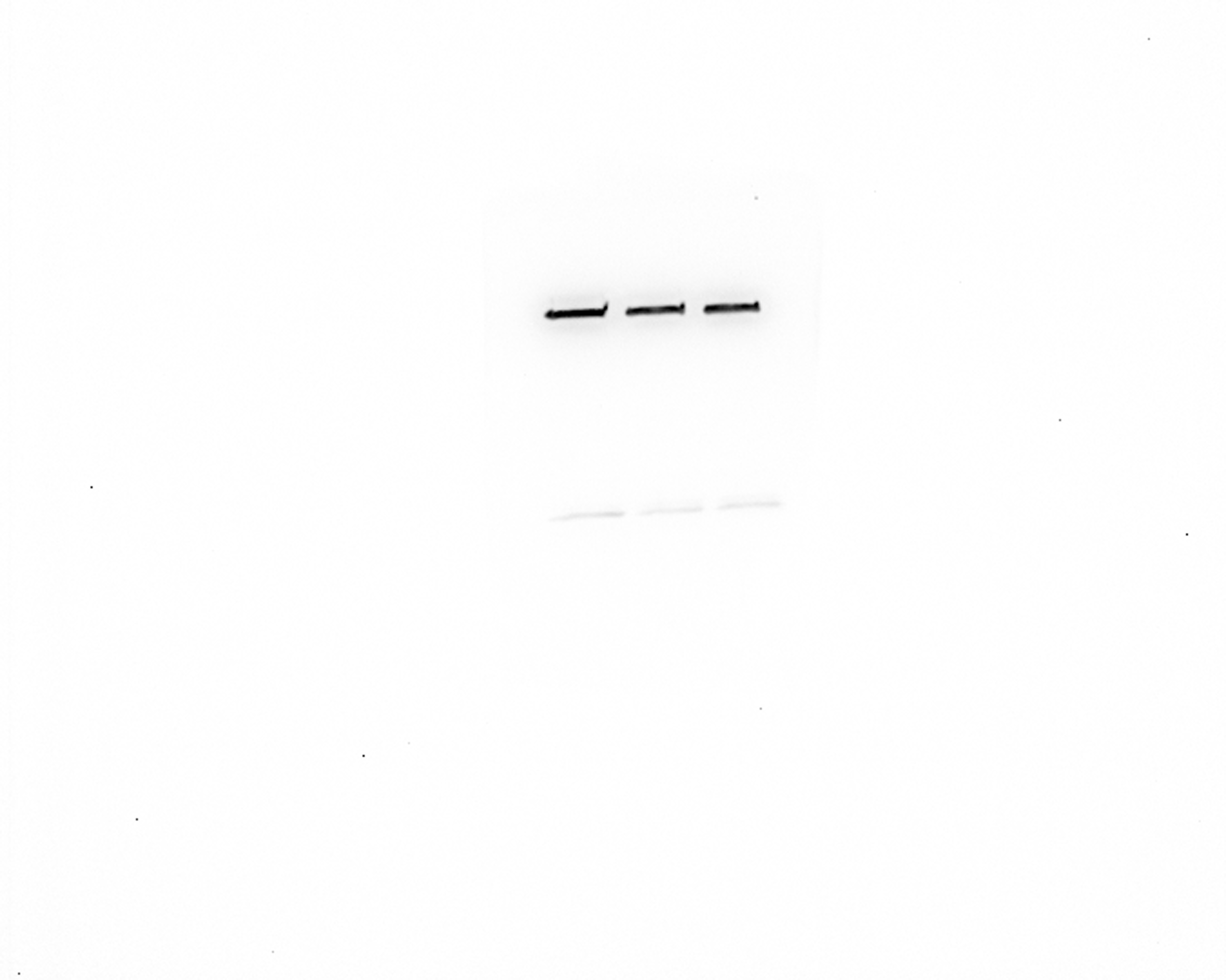

Supplement: Supplementary file 8 — Unprocessed western blots. [file 41477_2025_2135_MOESM8_ESM.zip › Source blots/Figure 1e/Figure 1e GFP input.tif]

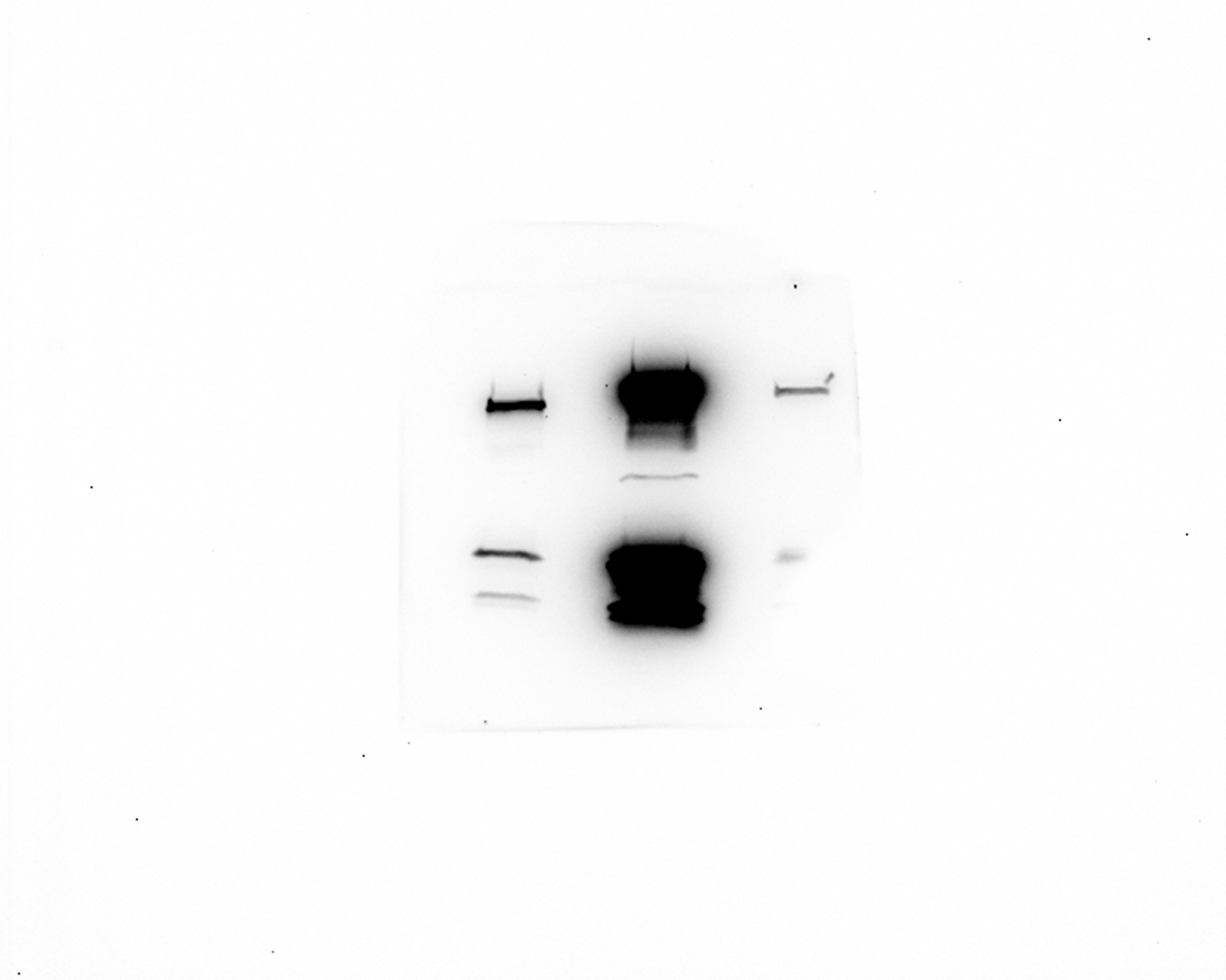

Supplement: Supplementary file 8 — Unprocessed western blots. [file 41477_2025_2135_MOESM8_ESM.zip › Source blots/Figure 1e/Figure 1e RFP Input.tif]

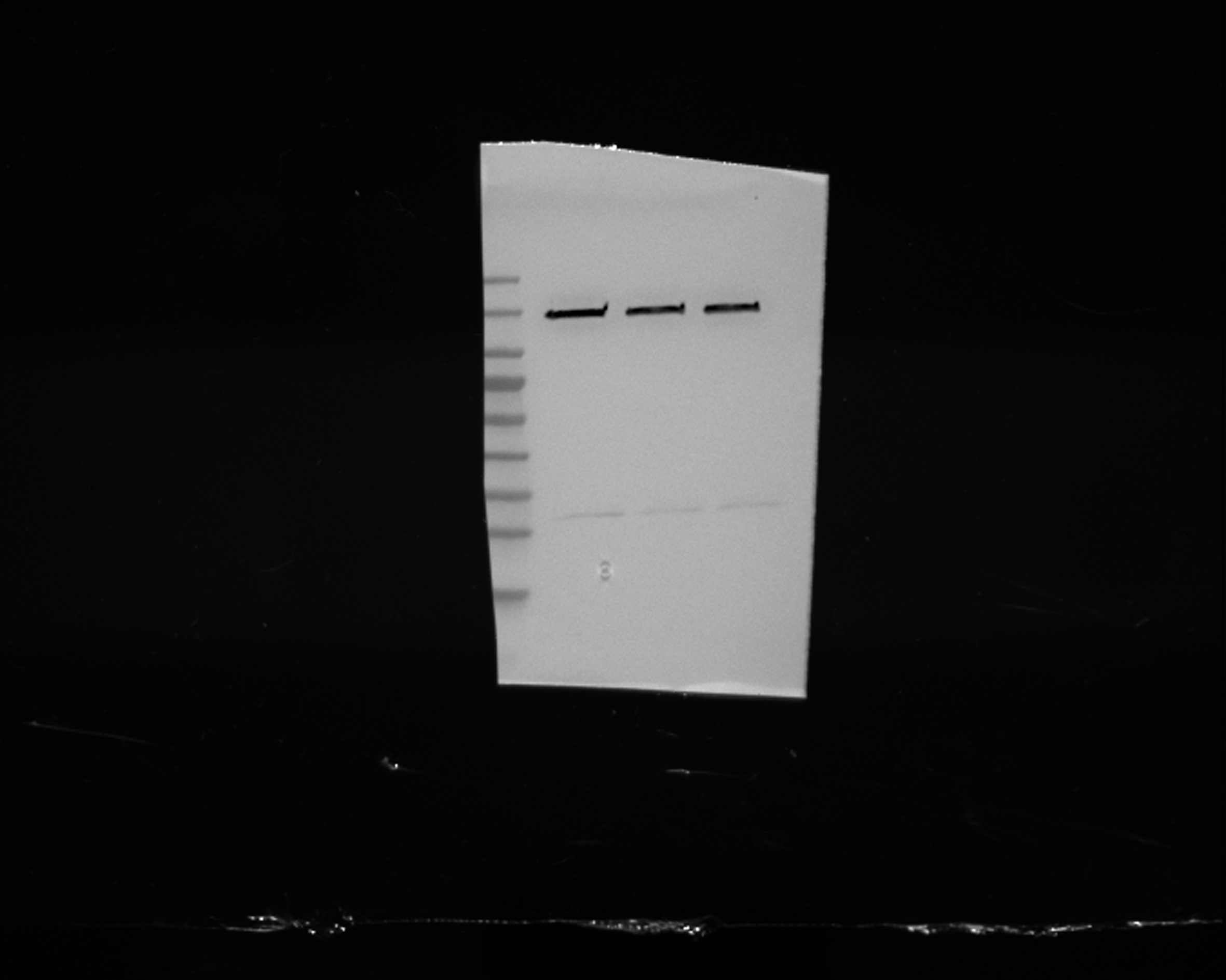

Supplement: Supplementary file 8 — Unprocessed western blots. [file 41477_2025_2135_MOESM8_ESM.zip › Source blots/Figure 1e/Figure 1e GFP input(Overlay).tif]

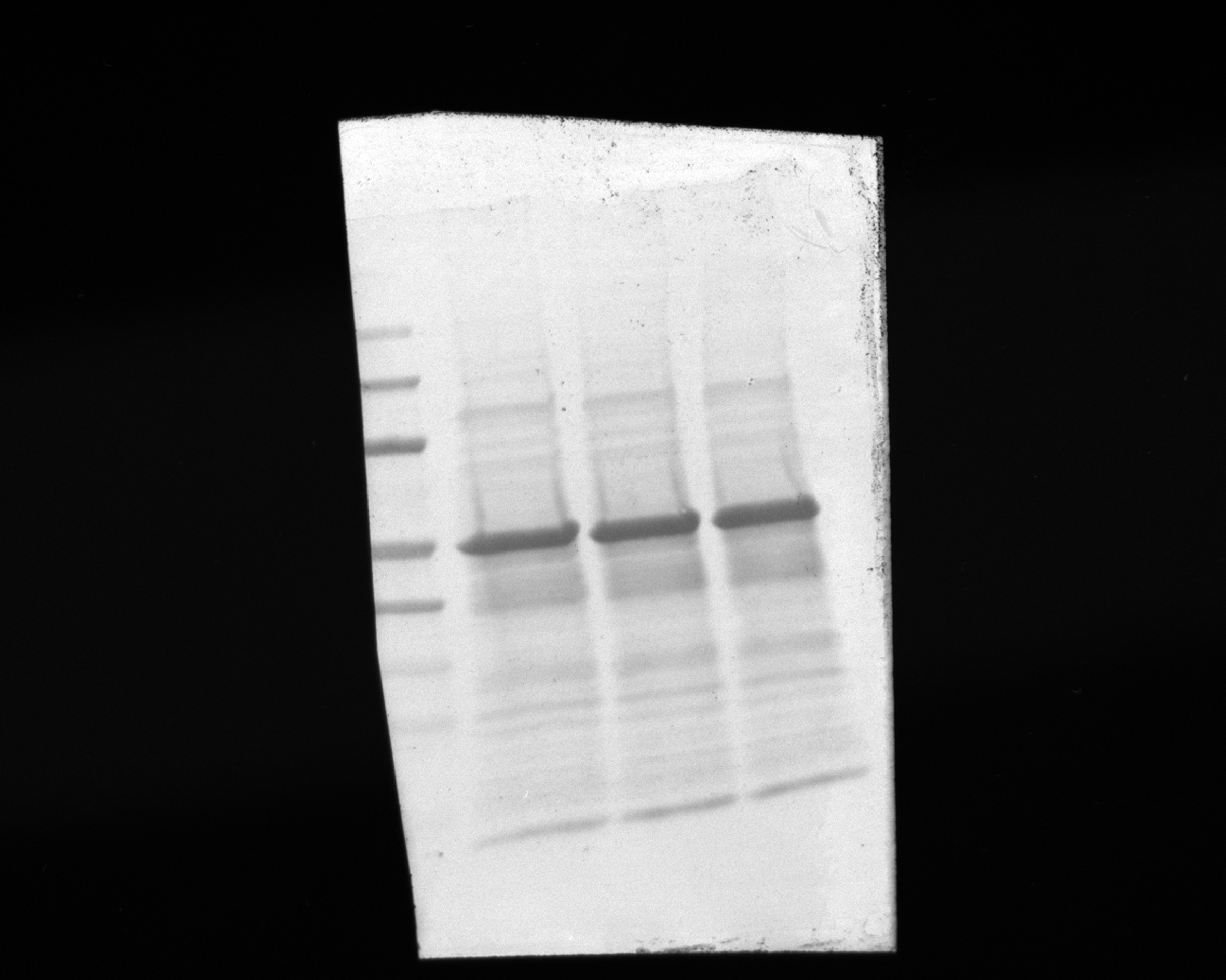

Supplement: Supplementary file 8 — Unprocessed western blots. [file 41477_2025_2135_MOESM8_ESM.zip › Source blots/Figure 1e/Figure 1e Ponceau.tif]

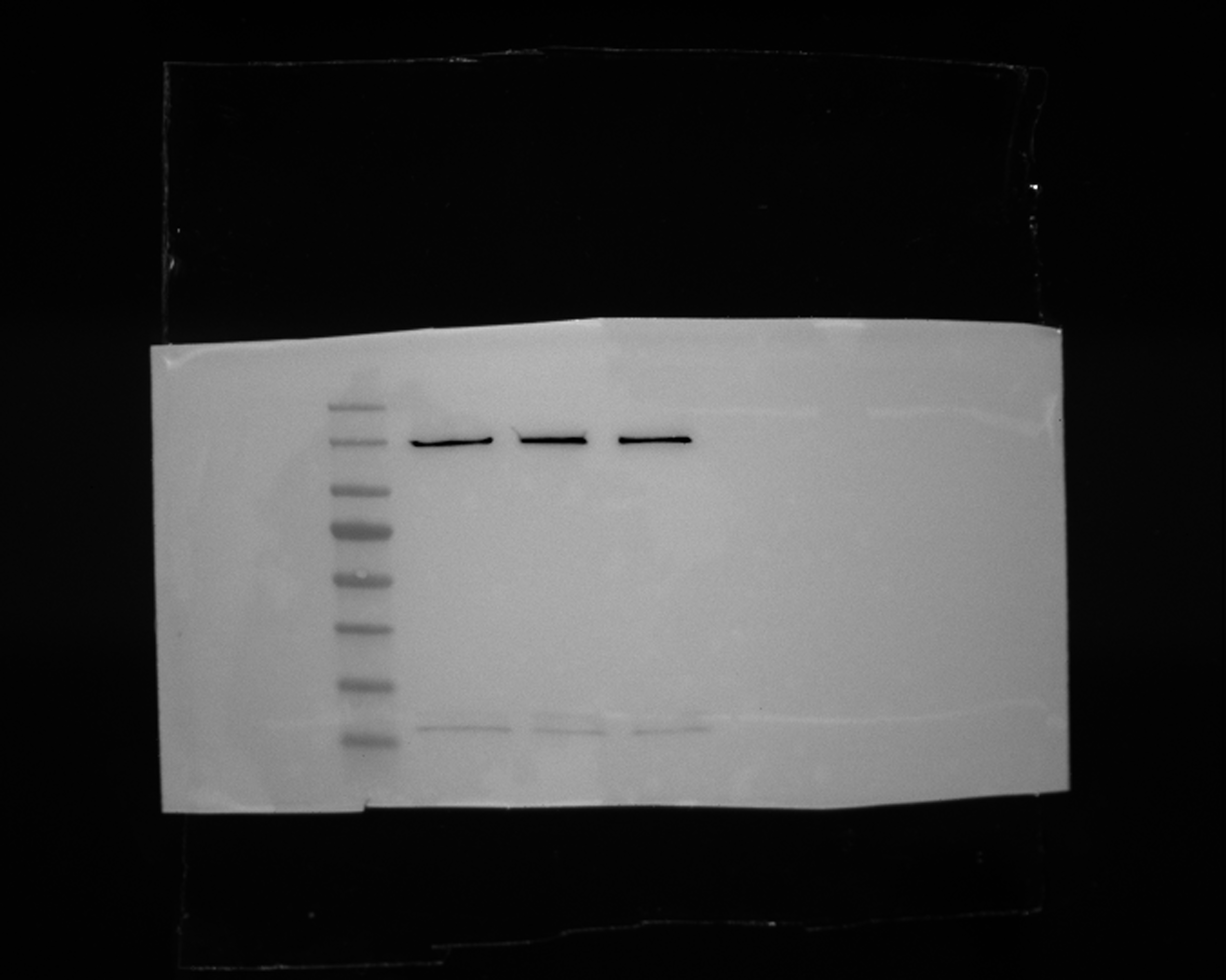

Supplement: Supplementary file 8 — Unprocessed western blots. [file 41477_2025_2135_MOESM8_ESM.zip › Source blots/Figure 1e/Figure 1e GFP IP(Overlay).tif]

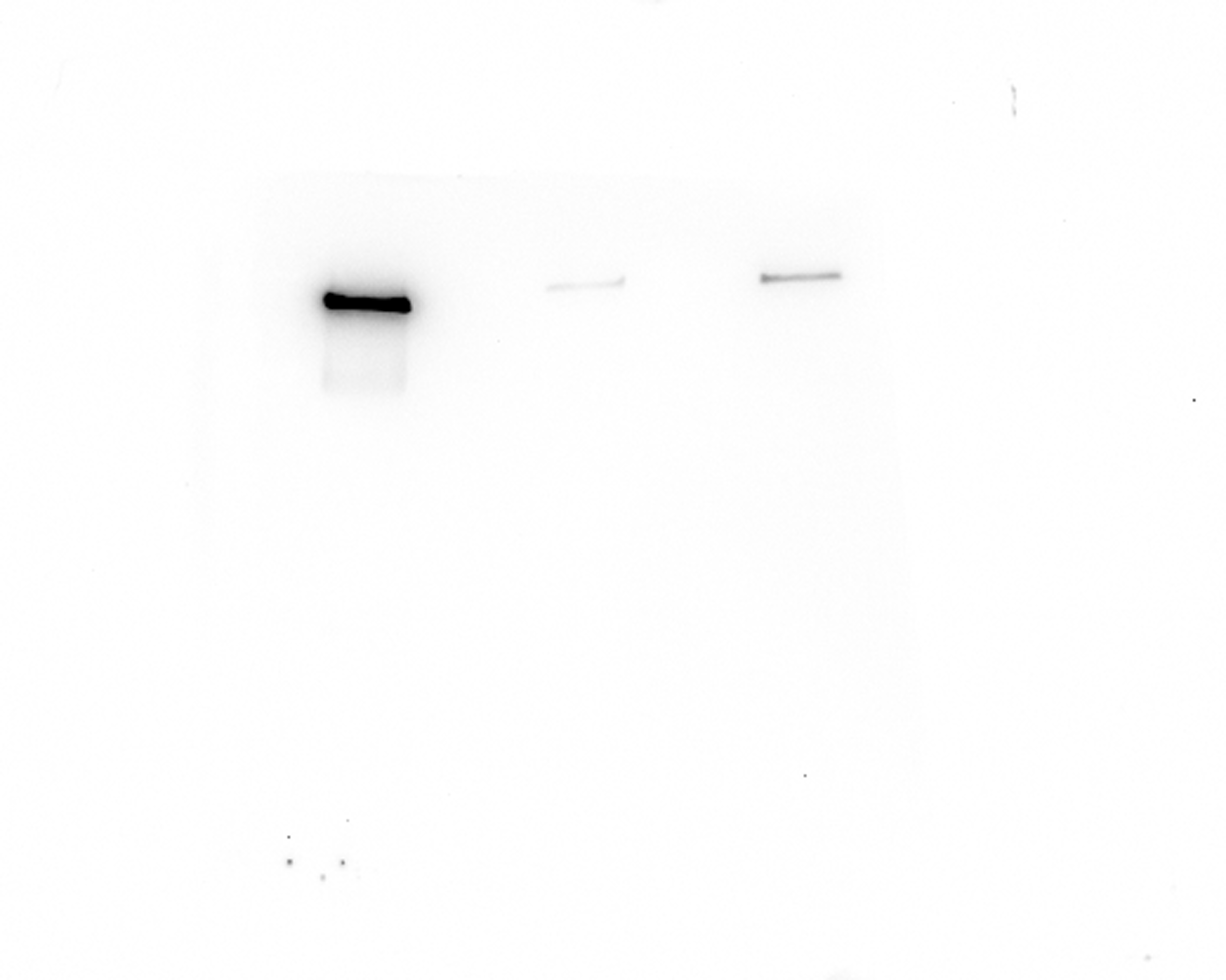

Supplement: Supplementary file 8 — Unprocessed western blots. [file 41477_2025_2135_MOESM8_ESM.zip › Source blots/Figure 1e/Figure 1e RFP IP.tif]

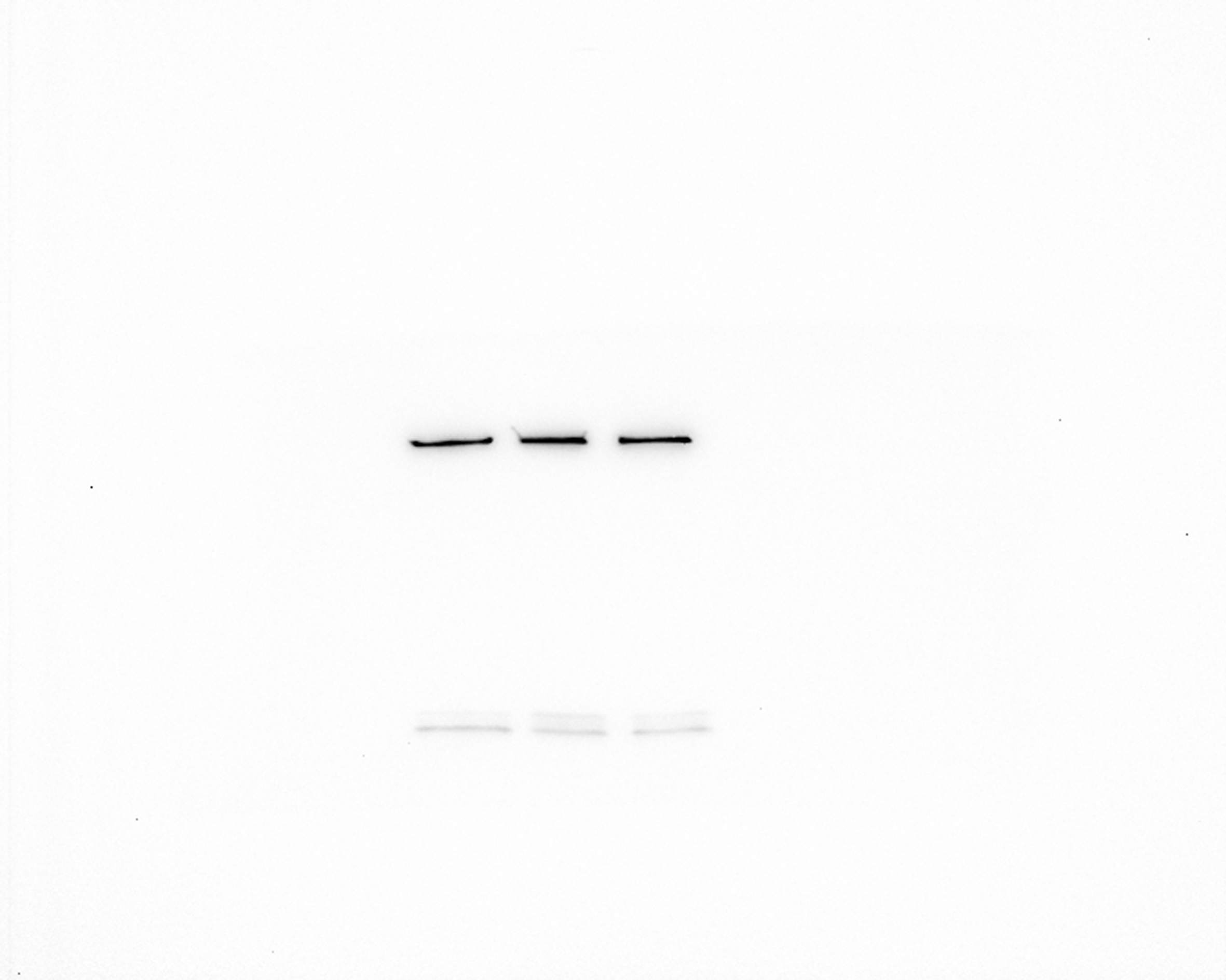

Supplement: Supplementary file 8 — Unprocessed western blots. [file 41477_2025_2135_MOESM8_ESM.zip › Source blots/Figure 1e/Figure 1e GFP IP.tif]

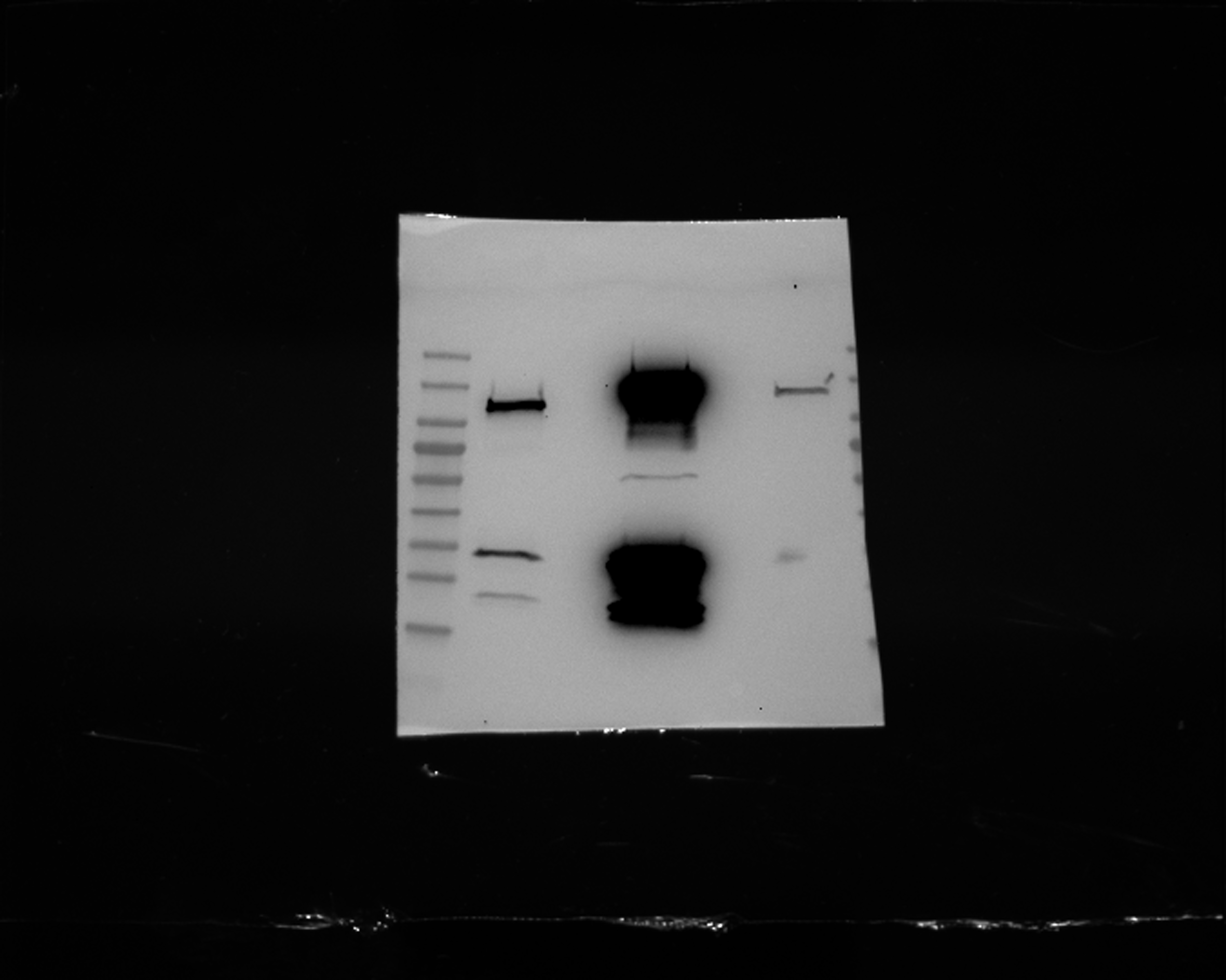

Supplement: Supplementary file 8 — Unprocessed western blots. [file 41477_2025_2135_MOESM8_ESM.zip › Source blots/Figure 1e/Figure 1e RFP Input(Overlay).tif]

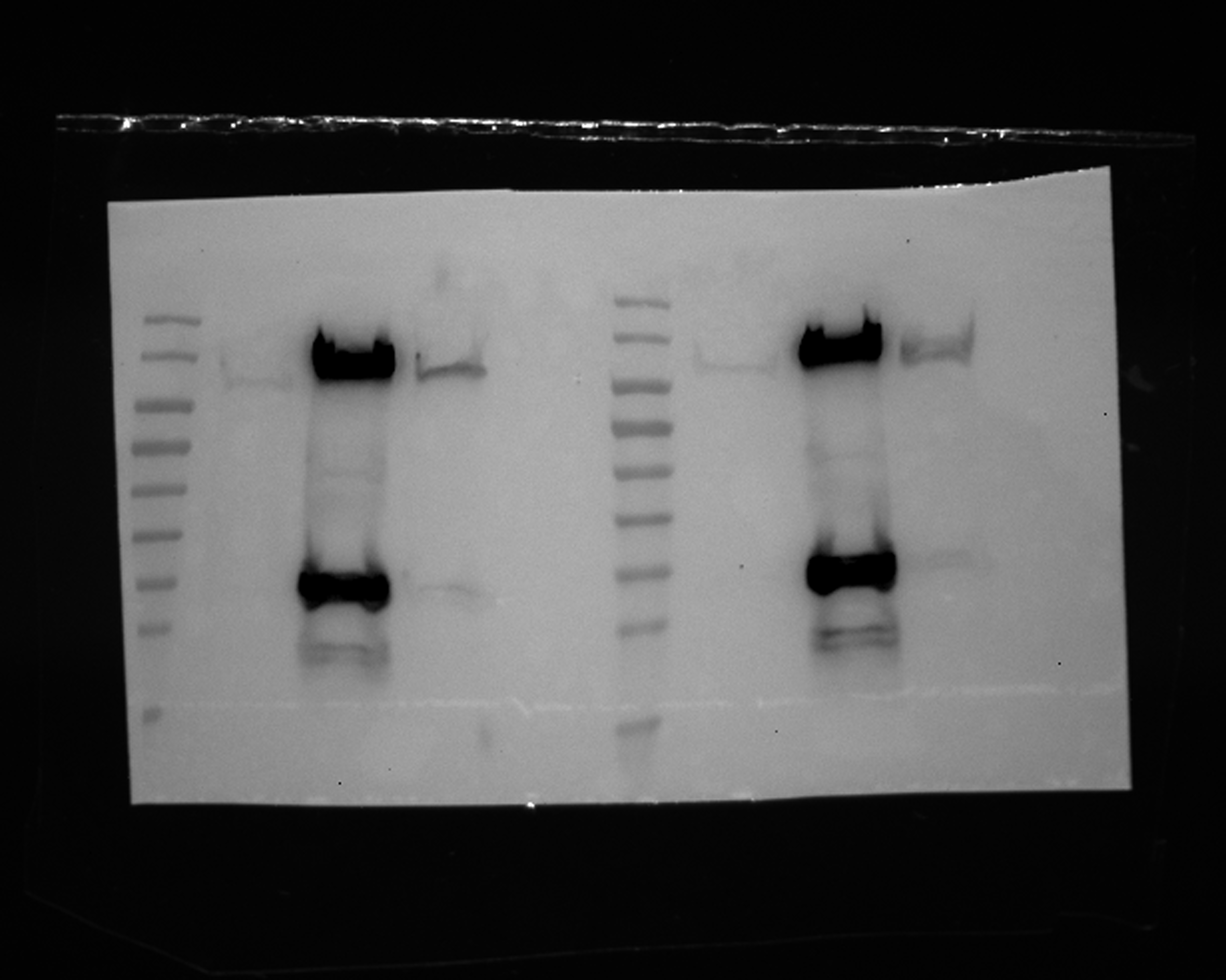

Supplement: Supplementary file 8 — Unprocessed western blots. [file 41477_2025_2135_MOESM8_ESM.zip › Source blots/Figure 6b/Figure 6b RFP input (Overlay).tif]

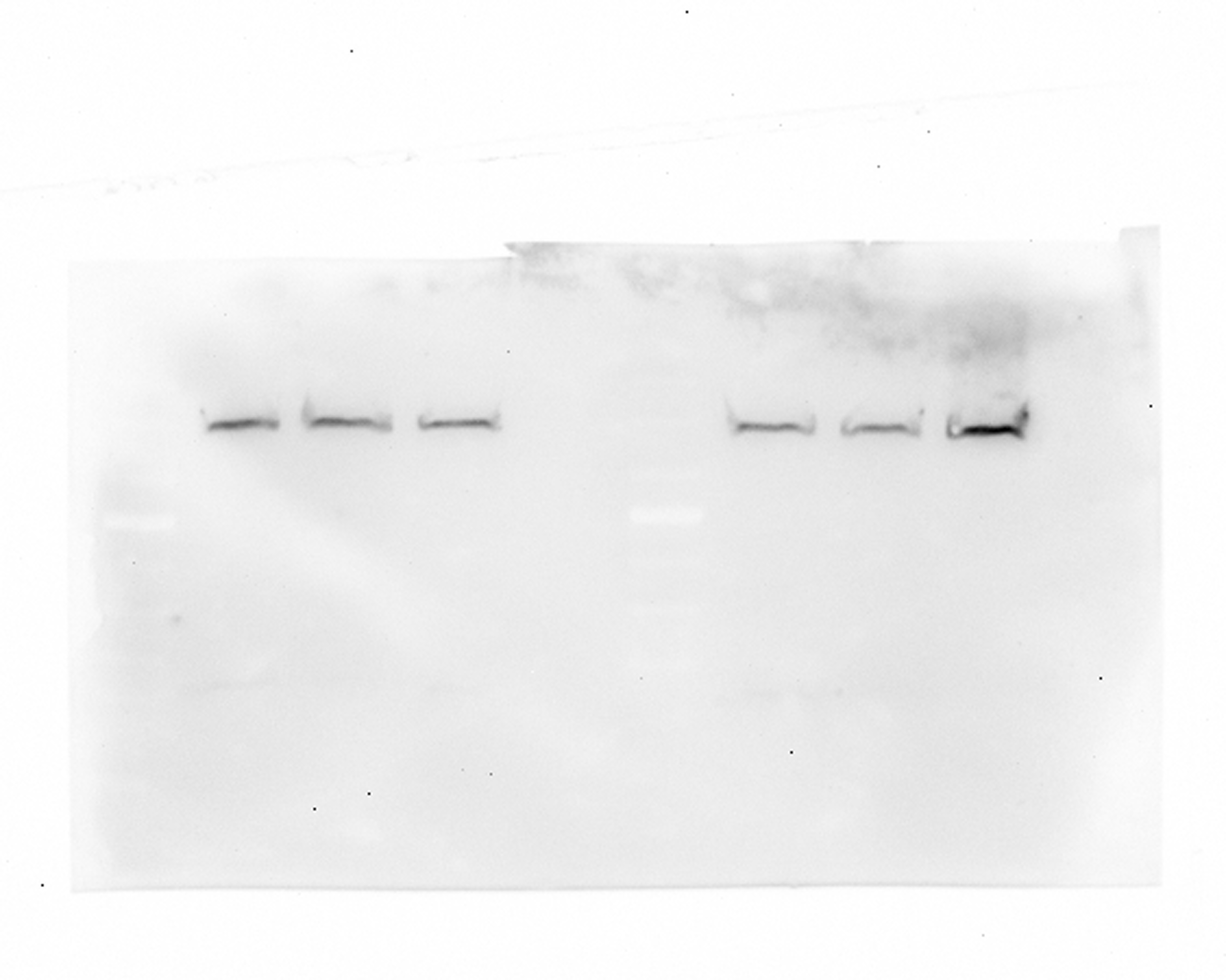

Supplement: Supplementary file 8 — Unprocessed western blots. [file 41477_2025_2135_MOESM8_ESM.zip › Source blots/Figure 6b/Figure 6b GFP Input.tif]

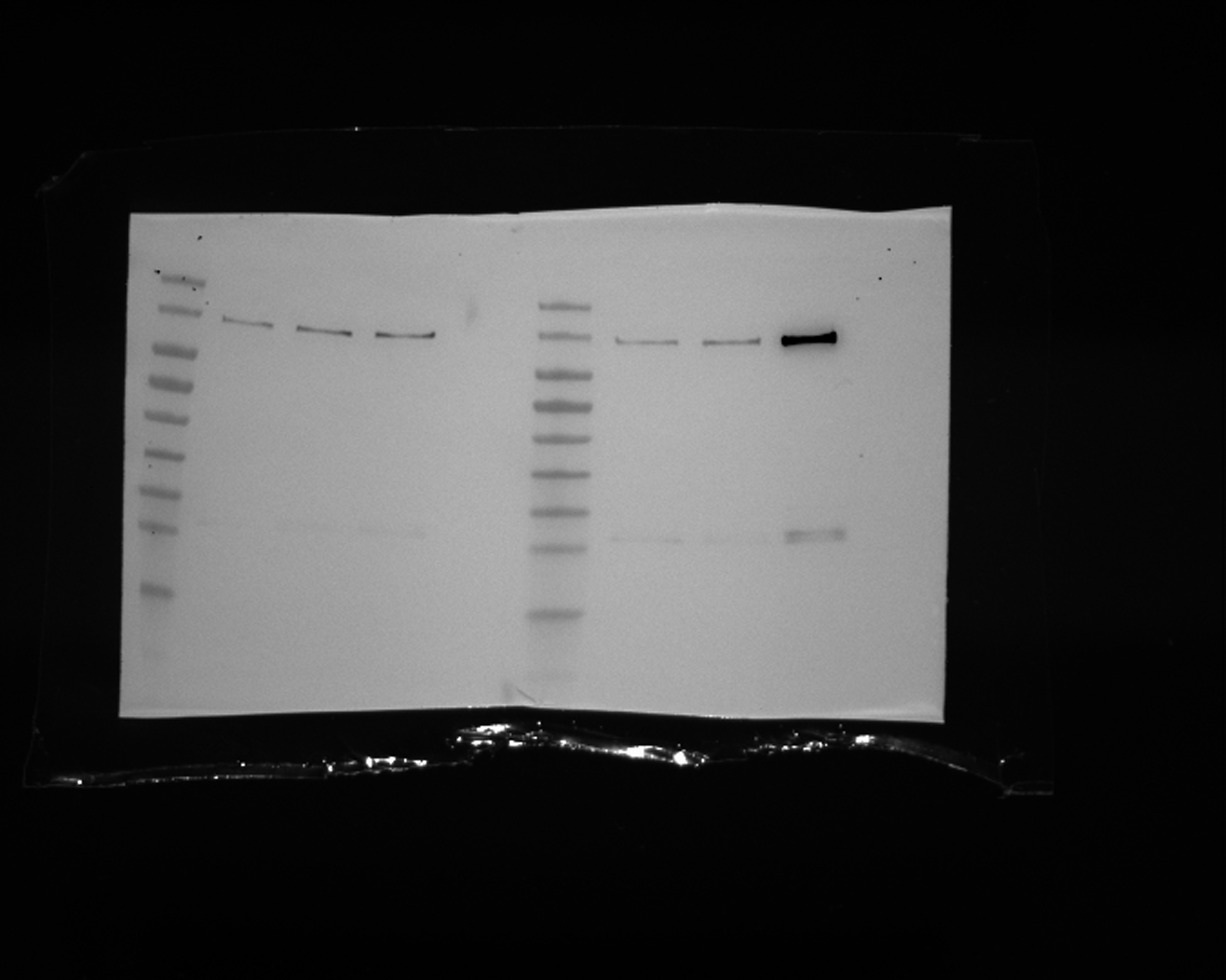

Supplement: Supplementary file 8 — Unprocessed western blots. [file 41477_2025_2135_MOESM8_ESM.zip › Source blots/Figure 6b/Figure 6b GFP IP (Overlay).tif]

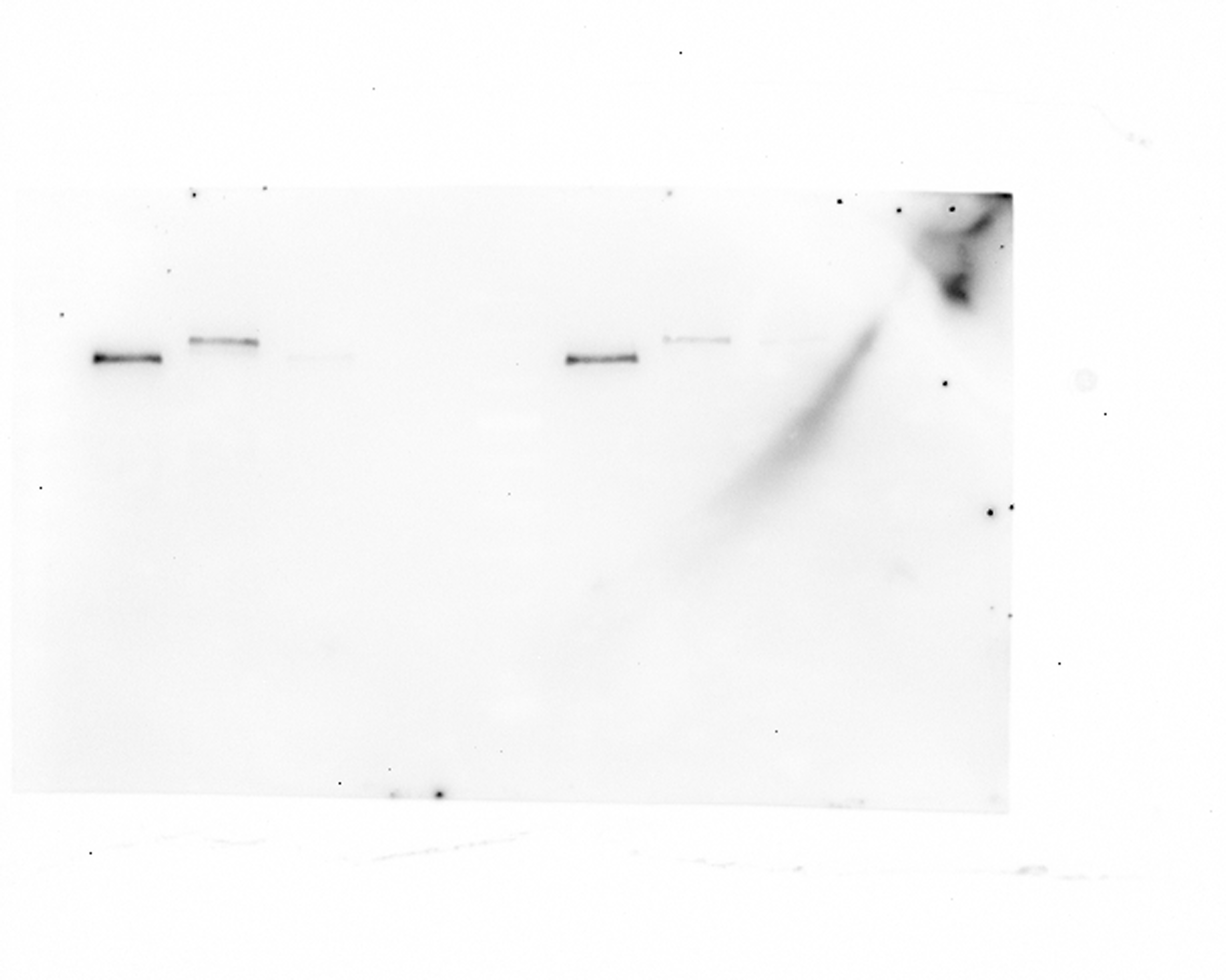

Supplement: Supplementary file 8 — Unprocessed western blots. [file 41477_2025_2135_MOESM8_ESM.zip › Source blots/Figure 6b/Figure 6b RFP IP.tif]

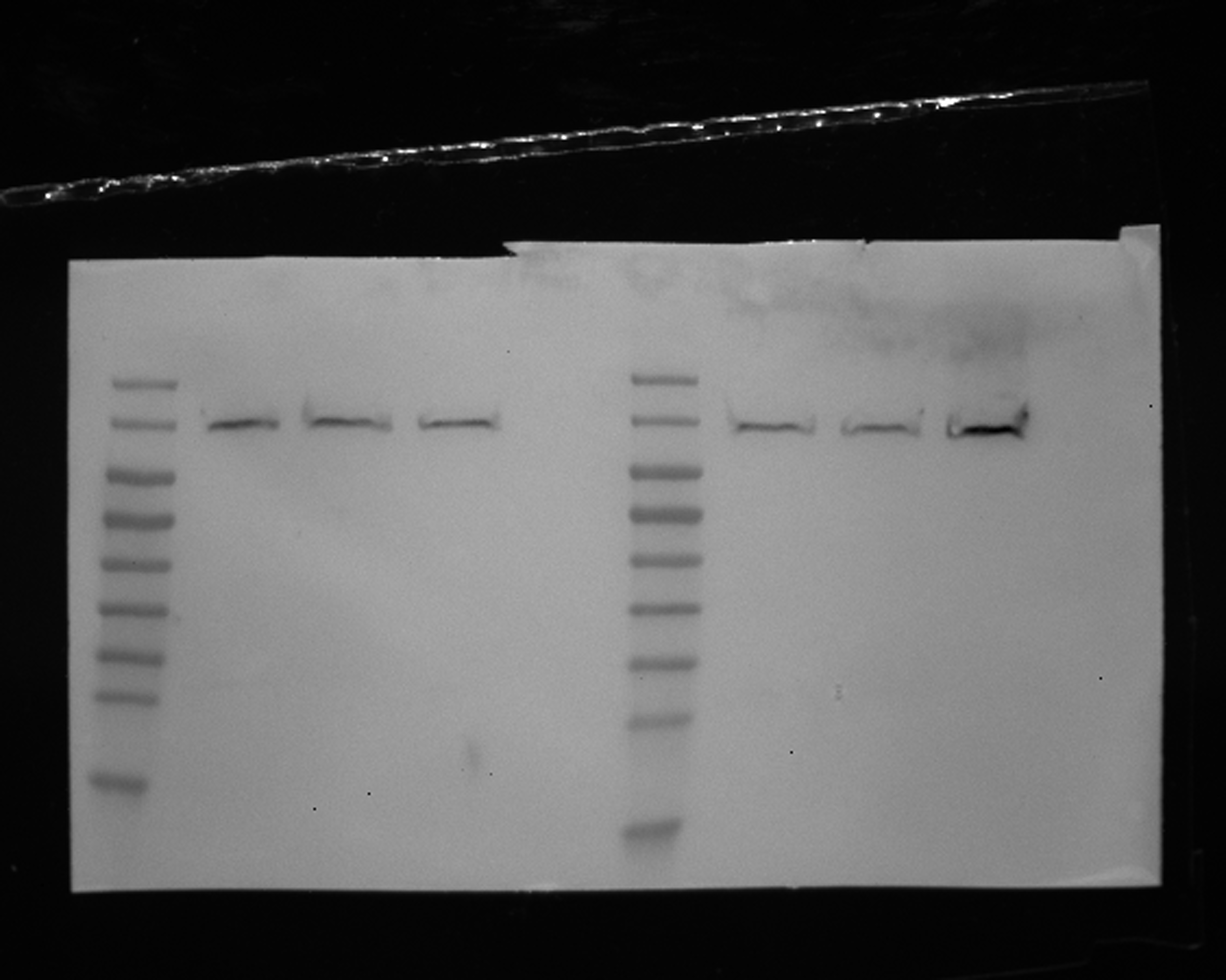

Supplement: Supplementary file 8 — Unprocessed western blots. [file 41477_2025_2135_MOESM8_ESM.zip › Source blots/Figure 6b/Figure 6b GFP Input (Overlay).tif]

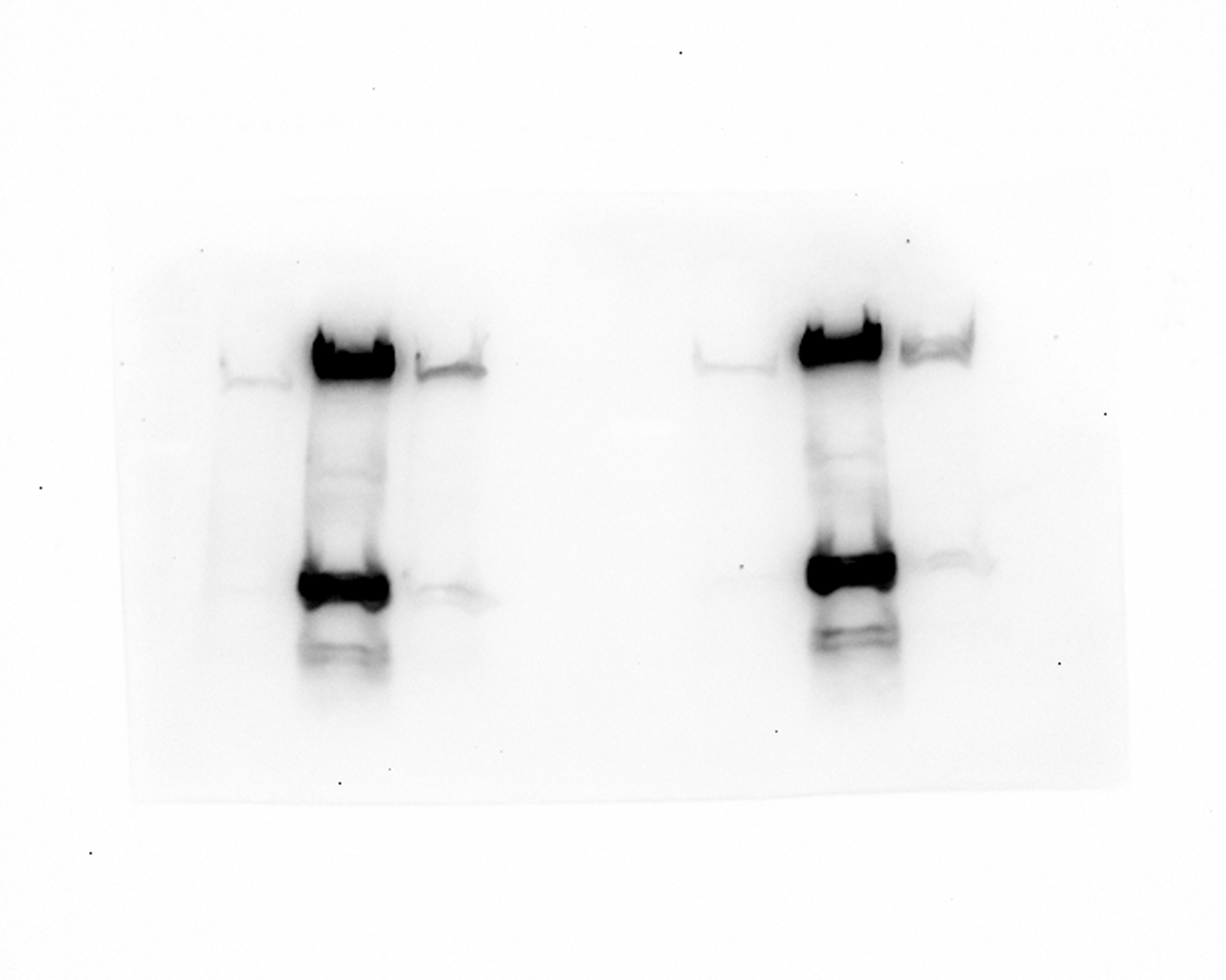

Supplement: Supplementary file 8 — Unprocessed western blots. [file 41477_2025_2135_MOESM8_ESM.zip › Source blots/Figure 6b/Figure 6b RFP input.tif]

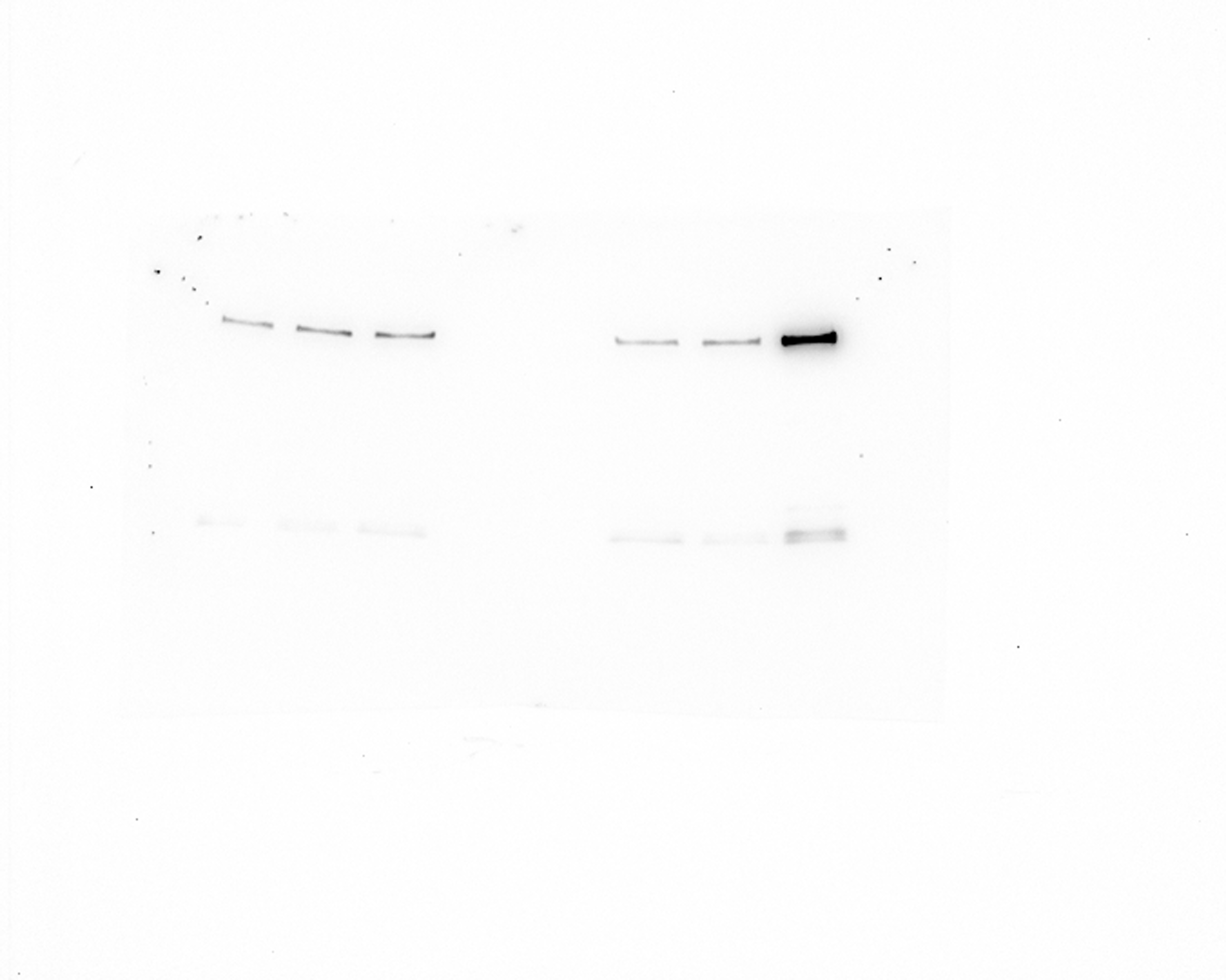

Supplement: Supplementary file 8 — Unprocessed western blots. [file 41477_2025_2135_MOESM8_ESM.zip › Source blots/Figure 6b/Figure 6b GFP IP.tif]

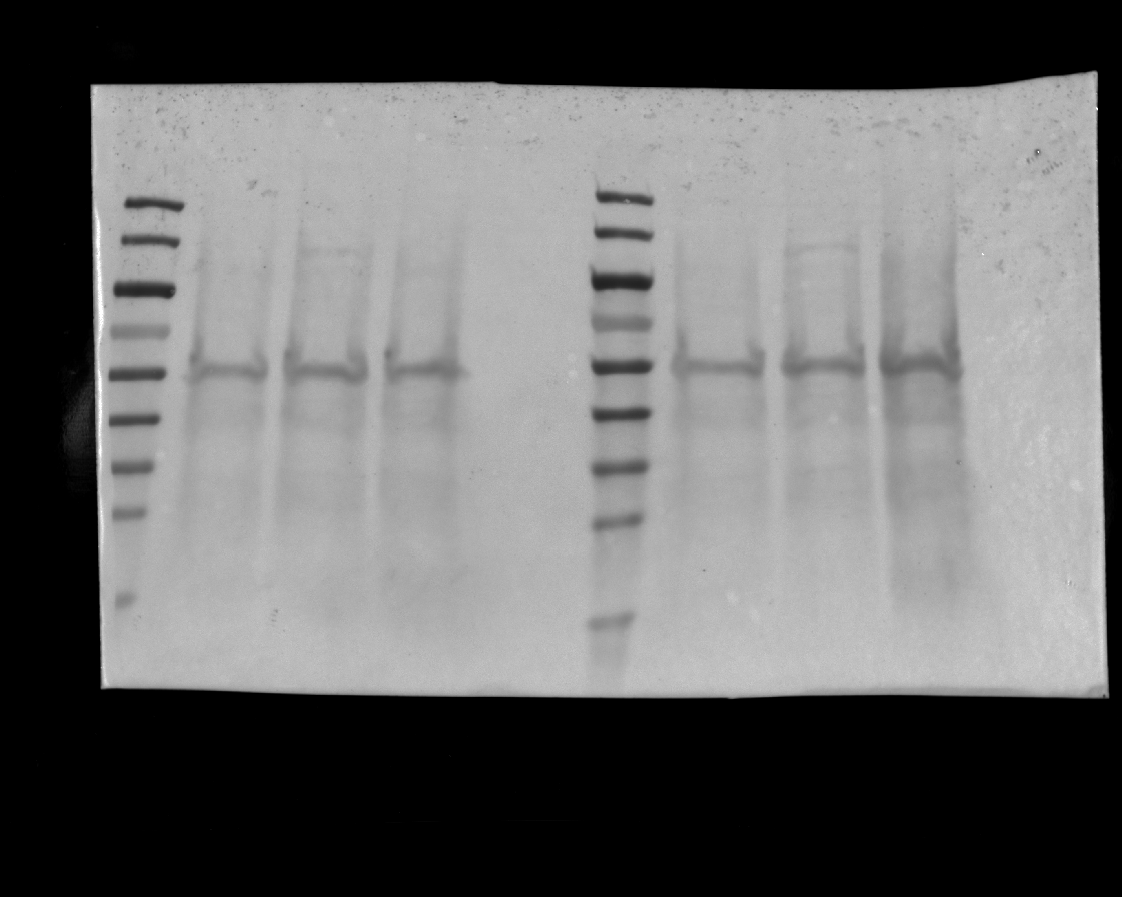

Supplement: Supplementary file 8 — Unprocessed western blots. [file 41477_2025_2135_MOESM8_ESM.zip › Source blots/Figure 6b/Figure 6b Ponceau.tif]

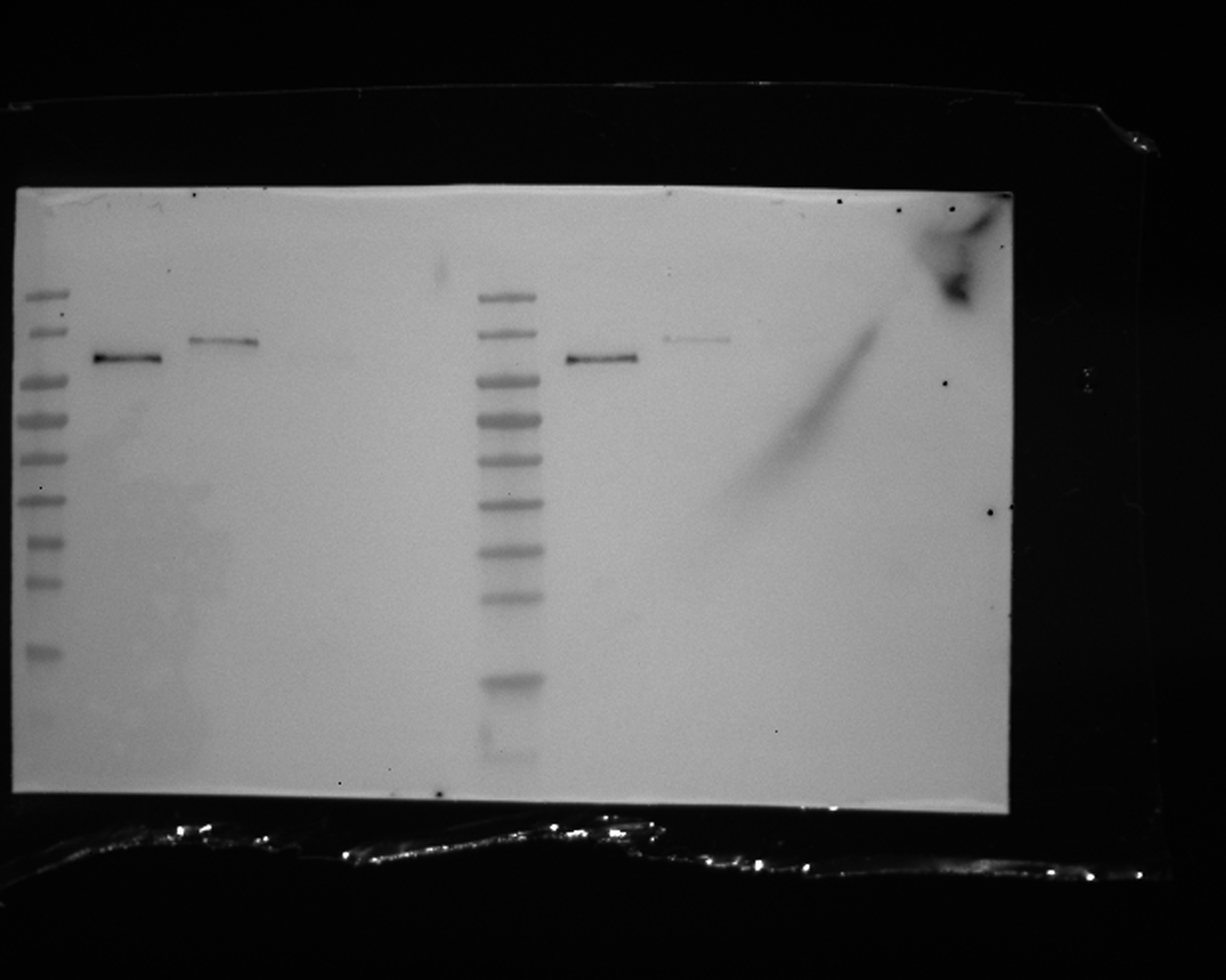

Supplement: Supplementary file 8 — Unprocessed western blots. [file 41477_2025_2135_MOESM8_ESM.zip › Source blots/Figure 6b/Figure 6b RFP IP(Overlay).tif]

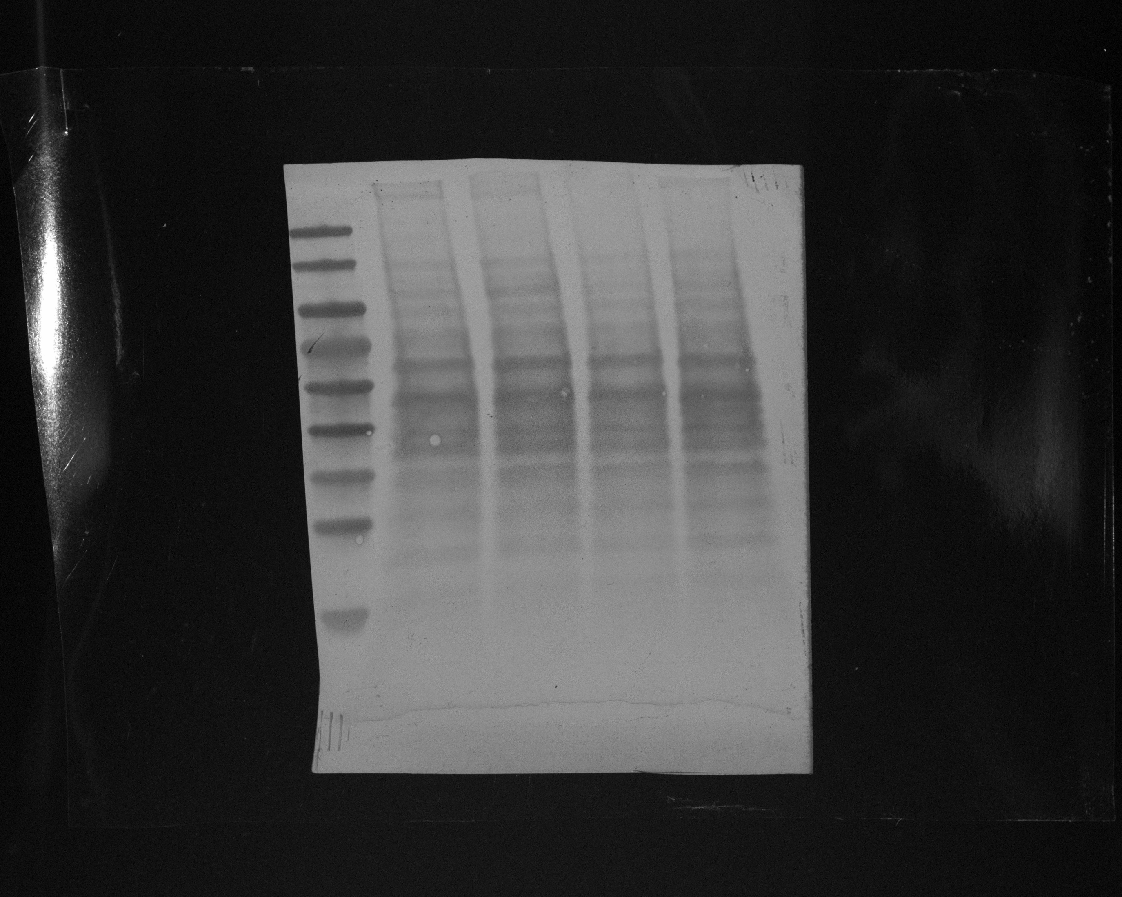

Supplement: Supplementary file 8 — Unprocessed western blots. [file 41477_2025_2135_MOESM8_ESM.zip › Source blots/Extended Data Figure 10b/CBS/Ext Fig 10b CBS.tif]

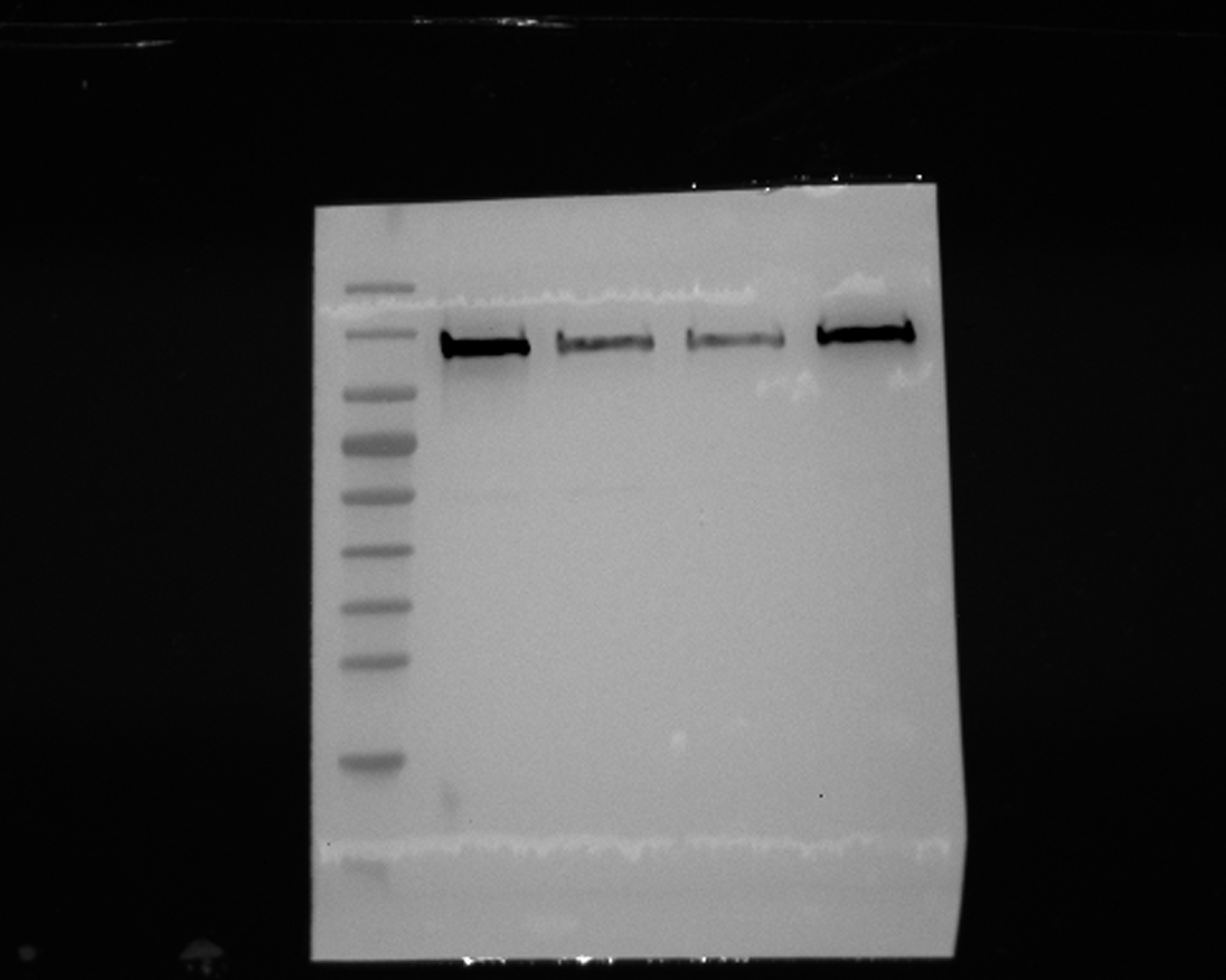

Supplement: Supplementary file 8 — Unprocessed western blots. [file 41477_2025_2135_MOESM8_ESM.zip › Source blots/Extended Data Figure 10b/AD/Ext Fig 10b AD.tif]

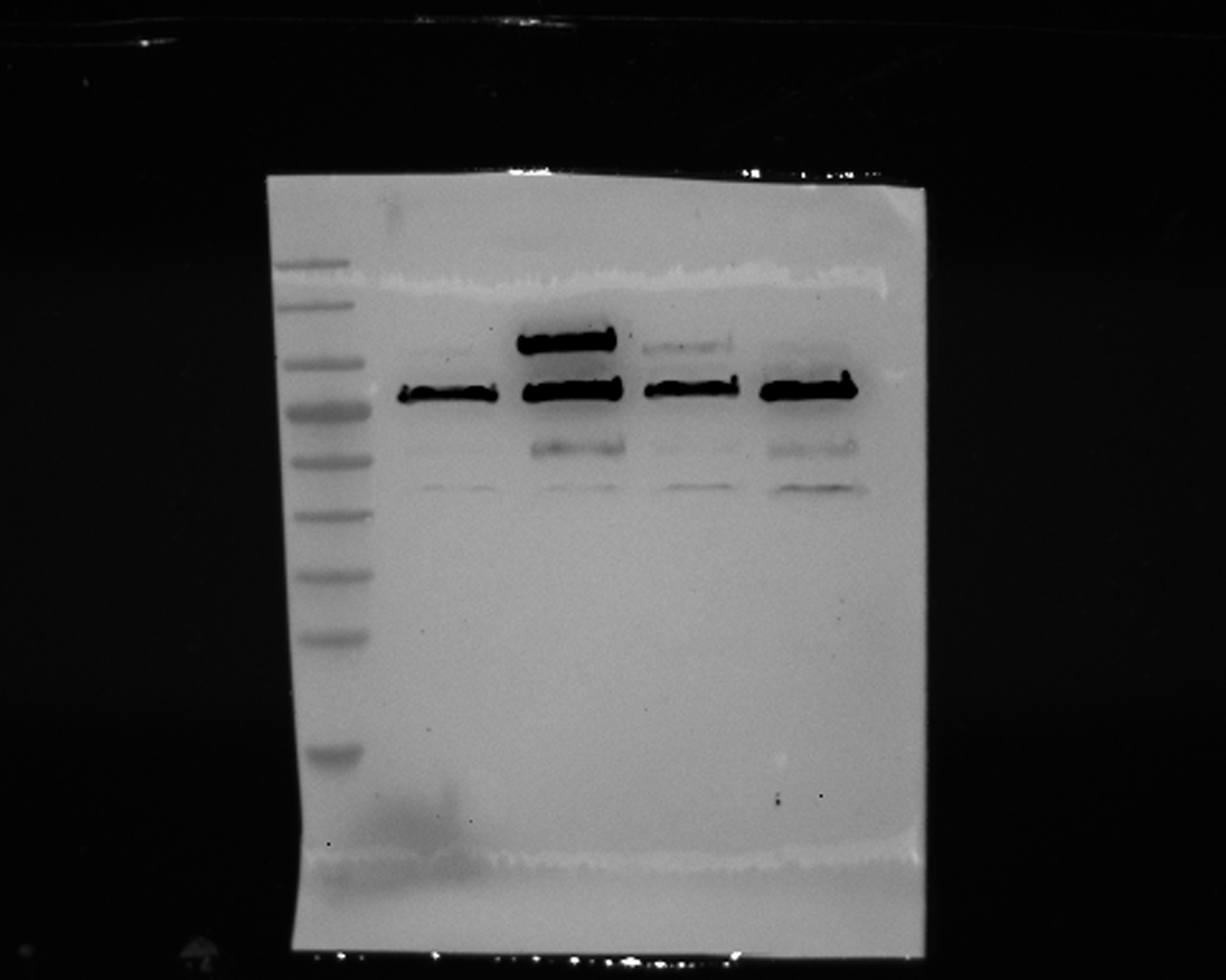

Supplement: Supplementary file 8 — Unprocessed western blots. [file 41477_2025_2135_MOESM8_ESM.zip › Source blots/Extended Data Figure 10b/BD/Ext Fig 10b BD.tif]

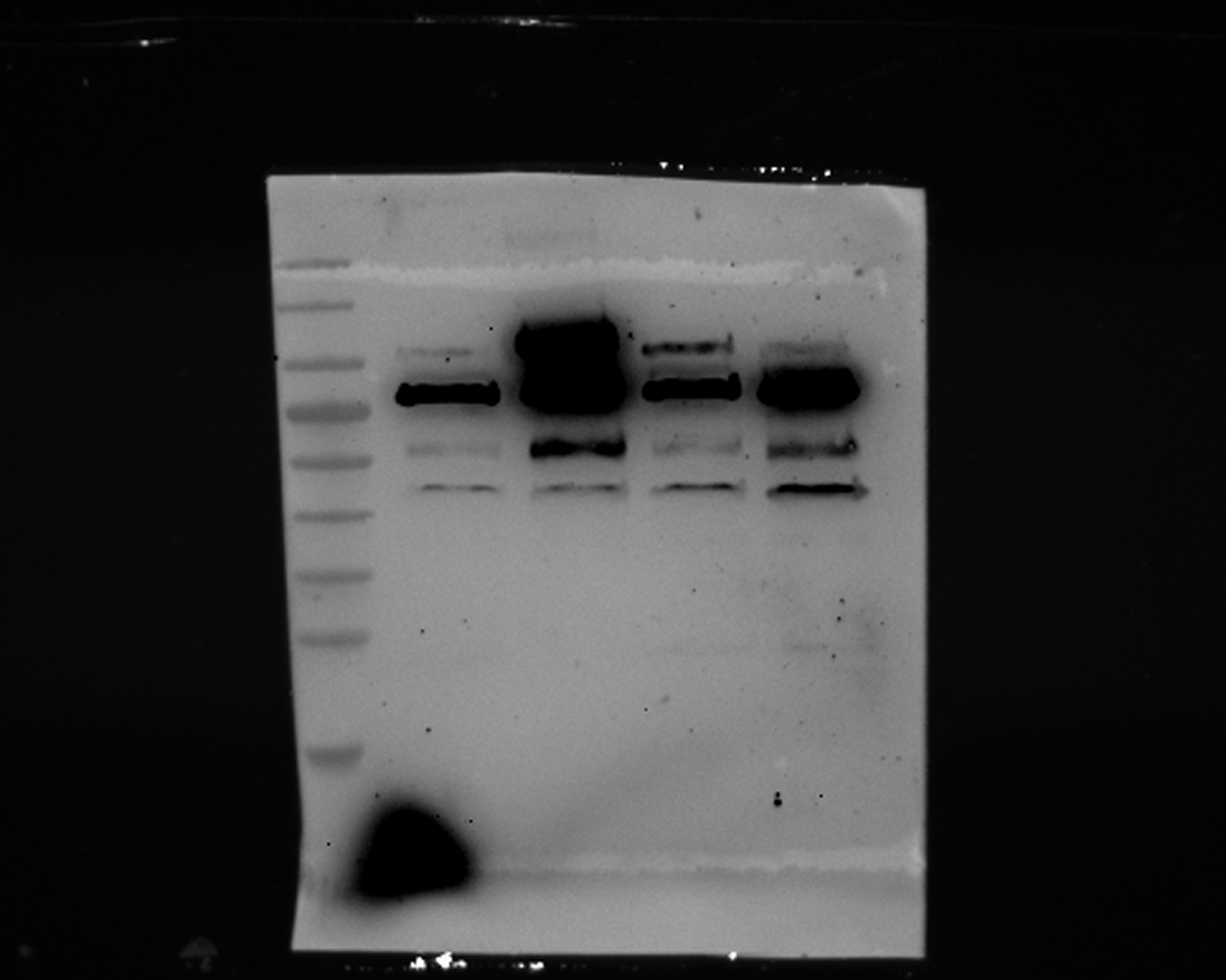

Supplement: Supplementary file 8 — Unprocessed western blots. [file 41477_2025_2135_MOESM8_ESM.zip › Source blots/Extended Data Figure 10b/BD long/Ext Fig 10b BD long.tif]
